# Supplementary material for: Phase II multicentre, double-blind, randomised trial of ustekinumab in adolescents with new-onset type 1 diabetes (USTEK1D): trial protocol
Source: BMJ Open. 2021 Oct 18;11(10):e049595. doi: 10.1136/bmjopen-2021-049595 (PMC8524290; doi:10.1136/bmjopen-2021-049595)
Supplement: Supplementary data [file bmjopen-2021-049595supp001.pdf]

[Insert local headers]

A research study to see if the medicine Ustekinumab can make diabetes easier to manage

CONTACT DETAILS FOR STUDY TEAM:

NURSE:

DOCTOR:

EMERGENCIES:

FOR YOUNG PEOPLE AGED 12-15 YEARS OLD

We would like you to help us with our research study. Please read this information carefully and talk to your parent or carer about the study. Ask us if there is anything that is not clear or if you want to know more. Take time to decide if you want to take part. It is up to you if you want to do this. If you decide not to take part, then that is fine, you will be looked after by your doctors just the same.

Please look at our video explaining the trial at www.type1diabetesresearch.org.uk/current-trials. The blue box below and the video contains the key points about the study. If you would like to know more, please read the rest of this leaflet.

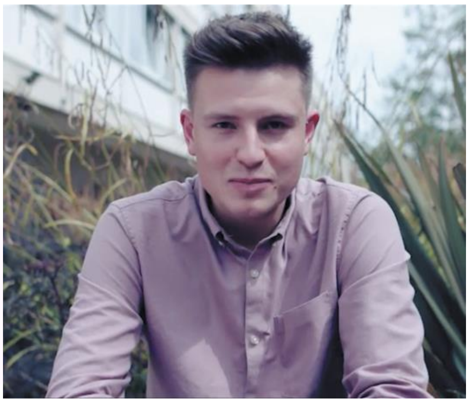

KEY POINTS ABOUT THE STUDY:

- We want to see if the study medicine, **Ustekinumab**, can help people with Type 1 Diabetes (the type you have). The medicine works by "protecting" some of the cells in the body that still produce insulin to help make diabetes easier to manage.
- The study will involve injections of the study medicine or a placebo (a "dummy" medicine that has no effects) every 1-3 months. These injections are given under the skin like insulin injections and will be done by the study doctor or nurse. Neither you nor the research team will know if you are receiving the study medicine or the placebo.
- The medicine is already being used to treat other illnesses quite safely.
- You will be asked to come into your local hospital or research centre for 10 study visits over a 15 month period, but where possible this will be on the same day as your routine hospital visits and 3 of the visits can sometimes be done by a research nurse at your home.
- We will ask you to provide extra blood and urine (wee) samples to check that it is OK for you to take part in the study and to check your health during the study. We will also use them to do some special tests in laboratories.
- You will receive a small gift voucher for each study visit you come to.
- You will wear a flash glucose monitor ("Freestyle Libre") on your arm to check your blood glucose levels for 2 weeks before each study visit. You can keep this for use at home for the rest of the time you are in the study if you want to.
- You can stop taking part in the study at any time and do not have to give a reason why.

Gregory JW, *et al. BMJ Open* 2021; 11:e049595. doi: 10.1136/bmjopen-2021-049595

WHY IS THIS STUDY BEING DONE?

This study is being done to see if a medicine called **Ustekinumab** can help to "protect" the cells in the body that produce insulin in young people recently diagnosed with Type 1 Diabetes (or T1D). At the time of diagnosis, most people your age with diabetes have some of their insulin-producing cells still working. It usually takes between 1 and 5 years before they stop working completely due to damage by the immune system. Sometimes these last few working cells can make enough insulin to make blood glucose levels stable and easier to control — this is called the "Honeymoon period". This period is only temporary and doesn't last. Ustekinumab, the study medicine, may make this period last longer by reducing the effects of the immune system on the insulin-producing cells in the pancreas.

WHY HAVE I BEEN ASKED TO TAKE PART?

You have been chosen because you are aged 12 - 18 years old and have recently been diagnosed with T1D.

DO I HAVE TO TAKE PART?

No. It is completely up to you whether or not you take part and you can always change your mind at any time. If you decide you don't want to take part, that's OK. Nobody will be upset and the medical care you get from your diabetes health care team will not be affected. You do not need to decide if you want to take part straight away. You can take your time and talk about the study with your family, friends and the study team if you want to. You need to let us know within 6 weeks of being diagnosed with diabetes, so that we can start the treatment early enough.

If you are interested in this study then you and your parent/carer should let your doctor or nurse know. Someone from the research team will get in touch to explain more about the study and answer any questions you have. You will be asked to sign a form to say that you are happy to take part. Your parent/carer will also sign a form to say that they agree too.

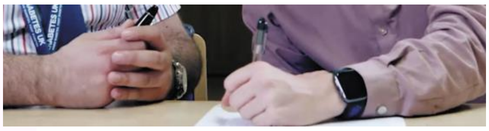

WHAT HAPPENS ONCE I HAVE AGREED TO TAKE PART?

SCREENING VISITS

Once the forms have been signed by you and your parent/carer we will do checks - "screening" - to see if you are suitable for the study. There are 2 screening visits during which we will do some tests to tell us about your health and your diabetes.

The first screening visit involves:

- A general health check by a doctor to make sure you can take part.
- We will take some blood samples (this will range from half a tablespoon to two tablespoons, depending on how much the hospital laboratory needs) from your arm.
- We will check whether you have an infection called TB (tuberculosis) or any viral infections which would stop you from taking part.
- We will also ask you to wee into a container so that we have a urine sample to test your kidneys and for girls, to make sure you're not pregnant.

The second screening visit involves:

- A **mixed meal tolerance test** (the Milkshake test) to test how much insulin your body still makes (see www.type1diabetesresearch.org.uk/current-trials). This involves coming to the hospital in the morning having not had breakfast. A small thin plastic tube will be put into a vein in your arm to take blood samples (with local anaesthetic ("numbing") cream/spray if you want it). This means that you will not need to have a needle in your arm for every blood sample we take during the test. We will need to take almost 3 tablespoons of blood at this visit in total. You then drink a flavoured drink that makes your body release insulin. Blood samples will be taken regularly through the tube over a period of 2 hours and your blood glucose is checked. During this time you can rest on a bed and can play on the internet or do other activities. At the end, the tube is removed. You then take an insulin injection depending on your blood glucose level and can leave the hospital when ready.

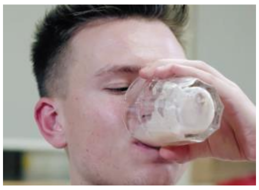

- We will ask you to complete a short questionnaire about your diabetes and how you feel. Your parent/carer will complete a similar one to you. You can ask the study nurse if you are unsure how to answer any questions.
- We will give you a free Freestyle Libre blood glucose monitor to wear at least two weeks before every visit (you can keep it on all the time if you want to).

If one of the screening tests tells us that you cannot take part, we will let you know as soon as possible.

If you and your parent/carer decide that it is easier to do both screening visits in one go, we can do this.

If the results of the screening tests are OK, then a computer will decide by chance whether you will receive the study medicine (Ustekinumab) or the placebo ("dummy" medicine).

Two thirds of young people taking part will receive the study medicine, and one third the placebo.

Neither, your family or the doctors and nurses will know which treatment you received until the end of the study.

STUDY VISITS

Once you enter the main study, you will be asked to come to 8 study visits over 52 weeks. Some of these visits may be on the same day as a routine hospital visit and 3 of the visits may be done by a nurse visiting you at home.

Activities at the visits may include:

- Check up by a nurse or doctor
- Urine sample tests
- Blood sample tests (between 3 and 4 tablespoons). If you feel unwell while this is being done, or you do not want to give the whole amount, the doctor or nurse can stop taking blood at any time.
- An injection of either the study medicine or the placebo. You will be asked to stay in the hospital for 1 hour after you receive the first injection so that the study team can make sure that you are OK.
- We will also download data that has been collected by a blood glucose monitoring device we will give you.
- Two study visits will involve mixed meal tolerance (Milkshake) tests.

FOLLOW UP

After the final study visit at week 52, we will check your hospital records for the next 12 months to find out how you are doing. You do not need to come into the hospital for a study visit. We may need to call you or your parent/carer up at home to check that you are OK and in good health.

WHAT ELSE WILL I BE ASKED TO DO?

You will be asked to complete a diary to record:

a) How much insulin you take in the two weeks before each study visit.
b) If you feel unwell or have to take any medicines during the study.
c) If you have any hypoglycaemic reactions (low blood glucose levels) that make you feel unwell during the study.

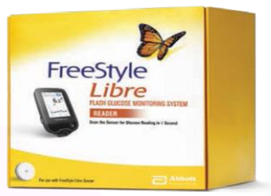

- We will ask you to test your blood glucose levels at home for at least 2 weeks before each visit using our FREE Abbott Freestyle Libre flash glucose monitoring system so you don't need to do extra finger prick tests for the trial. You will need to wear a sensor on your arm for the two weeks before the study visit if you want to take part and you can wear it every day if you want to. We will give you enough sensors to allow this for a year. We will also show you how it works.
- We will ask you to give a finger prick blood spot sample (just like a normal finger prick glucose test) before and 1 hour after the first meal of the day. This should be done once a week up for the first 28 weeks, then once a month for the next 6 months. You should do these at home with help from your parent/carer. We will show you how to do this - you prick your finger and then drop a spot of blood onto a special card.
- You will be asked to complete 3 questionnaires about your diabetes and how you are feeling at the start, middle and end of the study. Your parent/carer will also complete similar questionnaires for us.
- You will be asked not to have certain vaccinations before, during and immediately after the study.
- We will ask for permission to see your hospital records for a year after you finish your study visits to look at any changes to your insulin doses and glucose levels after stopping the treatment.
- A urine pregnancy test will be routinely done for all girls at each study visit.
- You need to be careful that you do not end up getting pregnant or making someone pregnant while you are in the study. We are asking everyone taking part to agree to use contraceptives before we can consider them for the study. Your parents/carers will be told about this requirement. Your GP or a pharmacist can advise on contraception if you don't want to discuss this with your parents/carers.

WILL THE STUDY HELP ME?

If you are in the group that receives the study medicine, it is possible that it will help your pancreas make insulin for longer. However, we cannot say this for certain until we have completed this study. During the study your diabetes will be very closely monitored. This will include regular check-ups with your local diabetes team including routine blood testing. You will have more time with the research team to discuss your diabetes and ask questions than at a normal clinic appointment.

You will be given an Abbott Freestyle Libre blood glucose monitoring system that you can use for the whole year to monitor your blood glucose levels without extra finger prick tests. You must wear this for the two weeks before the study visit if you want to take part. The treatments will stop at week 44 and you will not receive any further injections of the study drug / placebo during the trial.

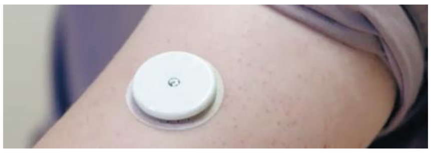

Gregory JW, *et al. BMJ Open* 2021; 11:e049595. doi: 10.1136/bmjopen-2021-049595

WHAT IF I DO NOT WANT TO TAKE PART ANYMORE?

Just tell your parents/carers to let us know. Nobody will be cross with you. You will still receive the same care from your doctors. We will still test the blood samples you have given so far unless you ask us not to.

WHAT IF THERE IS A PROBLEM OR SOMETHING GOES WRONG?

Tell your parents/carers as soon as possible if there is a problem so that they can let the study doctor or research team know and they will try to sort it out straight away. We will tell your parent/carer more about what to do if there is a problem when we talk to them about the study.

WHAT ARE THE POSSIBLE SIDE EFFECTS FOR ME IN PARTICIPATING IN THE STUDY?

You may get a bruise or a little discomfort where the needle goes in for the blood tests. We can use a cream or spray to numb the area from where we take blood if you like.

The FreeStyle Libre sensor may cause a slight rash for some people who might be allergic to the sticky part of the sensor. Please let the research team know if this happens.

During the Milkshake test, your blood glucose levels may be higher than usual because you will not have taken insulin immediately beforehand. The nurses and doctors will be available to help you make any changes to your usual insulin doses after this test.

The study medicine has not been tested in people with Type 1 Diabetes before so there may be some effects that we do not yet know about. Because the medicine acts on the immune system, there is a possibility that it could increase the risk of infections and cancer, but so far this has not been the case in people treated with this medicine for others diseases. It is also possible that you may get an allergic reaction to the treatment injection. We will ask you to stay for one hour after your first injection to check for any reactions.

We will need to do a chest x-ray. X-rays can cause damage to the body when you are older but we are only asking for one, which will hardly affect you.

If you feel ill at any time during the trial and go to your GP or the hospital, please show them the membership card you will be given so that they can contact the research team to ask about possible side effects.

WILL I RECEIVE ANY PAYMENT FOR TAKING PART?

As a thank you for you helping us with our research you will receive a £10 gift voucher for each treatment visit you come to and we'll give you a £30 gift voucher if you come to the final visit (visit 8) (that's £100 in total if you come to all visits). We will pay your parent/carer for any travel costs for attending study visits.

WHAT INFORMATION WILL YOU COLLECT AND HOW WILL IT BE KEPT PRIVATE?

We will ask for your name and contact details as well as information about your health which we get from your tests, your blood glucose monitor and your medical records. We will also ask you to complete a questionnaire at three time points. All of this information will be kept safely and nobody else will know that it is about you because your name will be removed and replaced with a study number. The people in our research team and diabetes care staff at the local hospital or research centre will know that you are taking part. We will also tell your family doctor (GP) that you are in the study if your parent/carer agrees to this.

People at the research laboratories will not know who you are when they test your samples. Your sample will be given a study number to replace your name so any study samples and data related to you will be anonymised. Cardiff University is responsible for the data we collect about you and will keep it secure for as long as is necessary.

WHAT WILL HAPPEN TO ANY SAMPLES I GIVE?

We want to test your blood and urine to better understand your diabetes and how the study medicine affects your diabetes. Samples will be tested in different research laboratories in the UK and may also be sent to special laboratories in Europe, America or Canada. These samples will not have your name on, only a study number so your identity will not be revealed.

We would like to keep any leftover blood samples in storage permanently for future research - we will ask you and your parent/carer to decide if they want you to do this.

WHAT SHOULD I DO NOW?

If you are interested in taking part, let your parents/carers know and they will get in touch with the study nurse/doctor.

Thank you for taking the time to read this information sheet and for considering taking part in this research study
