# Supplementary material for: Phase II multicentre, double-blind, randomised trial of ustekinumab in adolescents with new-onset type 1 diabetes (USTEK1D): trial protocol
Source: BMJ Open. 2021 Oct 18;11(10):e049595. doi: 10.1136/bmjopen-2021-049595 (PMC8524290; doi:10.1136/bmjopen-2021-049595)
Supplement: Supplementary data [file bmjopen-2021-049595supp002.pdf]

[Insert local headers]

USTEKID

National Institute for Health Research

A research study to see if the medicine Ustekinumab can make diabetes easier to manage

CONTACT DETAILS FOR STUDY TEAM:

NURSE:

DOCTOR:

EMERGENCIES:

FOR PARENTS OF YOUNG PEOPLE AGED 12-15 YEARS OLD

We would like you to help us with our research study. Please read this information carefully and talk to your parent or carer about the study. Ask us if there is anything that is not clear or if you want to know more. Take time to decide if you want to take part. It is up to you if you want to do this. If you do not, then that is fine, you will be looked after by your doctors just the same.

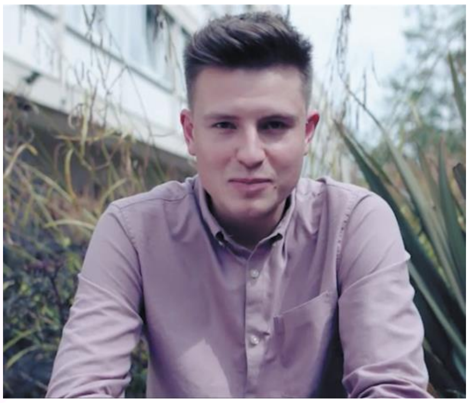

Please look at our video explaining the trial at www.type1diabetesresearch.org.uk/current-trials. The blue box below and the video contains the key points about the study. If you would like to know more, please read the rest of this leaflet.

KEY POINTS ABOUT THE STUDY:

- We want to see if the study medicine, **Ustekinumab**, can make Type 1 Diabetes (the type your child has) easier to manage. The medicine works by "protecting" some of the cells in the pancreas that still produce insulin from attack by the immune system.
- The study will involve your child having an injection every 1-3 months with either the study medicine, Ustekinumab or a placebo (a "dummy medicine"). These injections are given under the skin just like insulin injections and will be done by the study doctor or nurse. Neither you nor the research team will know if your child receives the study medicine or the placebo.
- The medicine is already being used to treat other illnesses quite safely. We will give you information about possible side effects before you decide if your child will take part.
- You will be asked to bring your child to your local hospital or research centre for 10 study visits over a 15 month period, but where possible this will be on the same day as your routine hospital visits. The first two will check if you are eligible to take part. Three of these visits can sometimes be done by a research nurse at home.
- We will ask your child to provide extra blood and urine samples to check that it is safe for them to take part in the study and to check their health, their blood glucose levels and how their immune system is working during the study.
- At three visits, they will have blood tests over a 2 hour period to see how much insulin their body is making.
- We will provide them with a Flash Glucose Monitor (Freestyle Libre) to wear for 2 weeks before each visit. They can keep the monitor for use at home for the whole time of the study.
- Your child will be offered a small gift voucher for each visit and your travel expenses will be paid.
- Your child can stop taking part in the study at any time and they do not have to give a reason why.

Gregory JW, *et al. BMJ Open* 2021; 11:e049595. doi: 10.1136/bmjopen-2021-049595

WHY IS THIS STUDY BEING DONE?

This study is being done to see if a medicine called **Ustekinumab** can help to "protect" the cells in the body that produce insulin in young people recently diagnosed with Type 1 Diabetes. It is caused by the body's own immune system damaging the cells in the pancreas that make insulin. Our aim is to develop a treatment that can slow this process by targeting the immune cells causing the damage.

At the time of diagnosis, most children have 10-20% of their insulin-producing cells still working. It usually takes between 1 and 5 years before they stop working completely. Sometimes these last few working cells can make enough insulin to make blood glucose levels stable and easier to control – this is called the "Honeymoon period". This period is only temporary and doesn't last. Ustekinumab, the study medicine, may make this period last longer by reducing the damaging effects of the immune system on the remaining insulin-producing cells in the pancreas.

Ustekinumab is currently given to adults and teenagers with particular skin and bowel problems and it is known to be safe to use and effective at treating those conditions.

WHY HAS MY CHILD BEEN ASKED TO TAKE PART?

Your child has been chosen because they are aged 12 - 18 years old and have recently been diagnosed with Type 1 diabetes.

DOES MY CHILD HAVE TO TAKE PART?

No. It is completely up to both your child and you whether or not they should take part and you or your child can always change your minds at any time. If you and your child are interested in this study then:

- Let us know by calling one of the people listed at the end of this information sheet.
- A member of the research team will contact you to explain more about the study and answer any questions you have.
- If your child wants to take part, they will be asked to agree to sign a form.
- As your child is under 16 years of age, you will need to sign a consent form to say that you agree for your child to take part. The consent form will also ask you to agree to complete three short questionnaires about your child's health. For this reason, it is preferable if the person most likely to attend the study visits with the child is the person who signs the consent form.
- You will be given this information sheet and a copy of your signed consent form to keep.

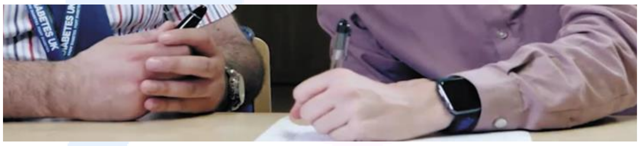

If you make a decision to allow your child to take part, ***you are still free to withdraw them from the study at any time without giving a reason***. This will not affect the medical care they get from their diabetes doctor in any way.

You do not need to decide if you want to allow your child to take part straight away. You can take your time and talk about the study with your child, family, friends and the study team if you want to. You need to let us know within 6 weeks of your child being diagnosed with diabetes, so that we can start the treatment early enough.

WHAT HAPPENS ONCE MY CHILD HAS AGREED TO TAKE PART?

VISIT	WHAT WILL HAPPEN	WHERE AND HOW LONG WILL IT TAKE?
Screening visits	Before we can start the treatment, we need to check that your child is eligible to take part in the study - this is called "screening". You will be asked to bring your child to your local hospital or research centre to talk about the study. This is where you will be able to ask questions.<br>If you are happy for your child to take part you will both be asked to sign consent forms before we begin the screening tests which involve:<br>Screening visit 1:	Your local hospital or research centre.<br>Approximately 1 hour for the first visit and 3 hours

Gregory JW, *et al. BMJ Open* 2021; 11:e049595. doi: 10.1136/bmjopen-2021-049595

	• Doing a general health check (this includes a general examination and measuring their height, weight and blood pressure).<br>• Asking about any medicine they are taking and any illnesses they have had or still have.<br>• Taking some blood samples (between 0.5 - 2 tablespoons) from their arm to check their general health and diabetes, as well as testing for infections such as TB (tuberculosis), hepatitis and HIV. Blood volumes vary according to your hospital's local testing procedures.<br>• Taking a chest X-ray to test for TB. There will be one other test for TB which will either be a blood test or a Mantoux test (a skin reaction test), depending on what your hospital's local procedures are.<br>• Taking a urine sample to test for infection, kidney function and a routine pregnancy test for all girls.<br><br>If one of these tests tells us that it is not safe for your child to take part, we will let you know straight away. If the tests are OK, then we will proceed with a second screening visit for a few more tests.<br><br>Screening visit 2:<br>• Doing a general health check.<br>• Asking about any medicine they are taking and any illnesses they have had or still have since their last visit.<br>• Taking a urine sample to test for infection, kidney function and a routine pregnancy test for all girls.<br>• You and your child will be asked to complete a short questionnaire about their diabetes and general health.<br>• Your child will be given a free blood glucose monitor (FreeStyle Libre) and sensors to use for the study. The sensor should be worn two weeks prior to every study visit but can be worn constantly if your child finds it helpful.<br>• A Mixed Meal Tolerance Test (or Milkshake test). This test tells us how much insulin their body is still making. During this test we also take additional blood samples to test the immune system and pancreas function.<br><br>On the day of the Mixed Meal Tolerance Test, you will need to make sure that your child has not eaten or drunk except water anything from midnight the night before onwards. They will also be asked to not take their early morning short acting insulin because they will not be eating breakfast. You will need to tell us your child's blood glucose levels on waking so that we can make sure it's OK for them to be tested. They will need to be between 4.0 and 11.1mmol/L for the screening visit to happen. If their blood glucose level runs low before coming to the hospital and needs to be treated, the test will be rescheduled but if they are higher, your child may be advised to take short acting insulin so that the visit can go ahead.<br><br>At the hospital, your child will have blood taken through a small plastic tube (cannula) which we will insert in their arm (using local anaesthetic cream/spray if they want it). This will stay in their arm during the test so that we can take blood samples more easily.<br><br>Then they will be given a milkshake to drink (various flavours available). The research doctor or nurse will take blood at fixed times over the next 2 hours. Over this time, less than 1 tablespoon (10ml) of blood will be taken from them in total. During this time, they can relax on a bed, play games, read or study. Once the test is completed we will give them something to eat and drink and they will receive insulin in whatever dose is needed. 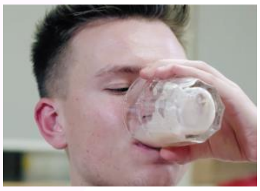<br><br>An additional 40mls (less than 3 tablespoons) will be taken at the same visit for testing in our laboratories.<br><br>The blood samples will be analysed within 2 weeks and if the test shows that they are still making some of their own insulin, you will be contacted by the research team to tell you that everything is OK for your child to be part of the study and to arrange the first injection.	for the second visit.

	If it is not convenient to have two separate visits for testing, we can arrange to combine the two sets of tests if you let us know beforehand. This is because the combined screening visit needs your child to be fasted on arrival.	
**Study Visit 1**	If your child is eligible to take part in this study, they will be randomly allocated to either the study medicine group or the placebo (a "dummy" medicine that has no effect) group. This is decided by chance using a computer programme before the study visit and neither you nor the research team will know until the end of the study what treatment they received.<br><br>2 out of every 3 people taking part will receive the study medicine compared with only 1 out of 3 getting the placebo. This is to give people a better chance of getting the study medicine.<br><br>Treatment visits will be booked in so that your child will receive injections at the required intervals. The second dose will be four weeks after the first. All other doses afterwards will be eight weeks apart. These will be booked in advance so that any issues with attendance can be identified as soon as possible (e.g. holidays, exams). Postponing a treatment visit may result in the treatment being stopped if too much time has passed because the levels of the study medicine in your child's body may go too low and won't work anymore.Your child will have a physical examination and tests will be done on their urine and blood samples (57.5ml which is about 3 and a half tablespoons) at this visit.<br><br>Then they will receive an injection of either the study medicine, Ustekinumab, or the placebo. Injections are given under the skin using a very small needle similar to the one that they already use for daily insulin injections.<br><br>They will be asked to stay in the hospital for 1 hour after they receive the injection so that the study team can make sure that there are no side effects and that your child are safe to leave.	Your local hospital or research centre.<br><br>Approximately 2 hours.
**Study Visit 2**<br><br>*4 weeks after the 1st visit*	Your child will have a physical examination and be asked questions about your health. We will also need a urine sample and a blood sample (up to 50.5ml which is just under 3 tablespoons). Then your child will receive an injection of either the study medicine, Ustekinumab or placebo, depending on which treatment group they are in.<br><br>We will also download the data stored on their blood glucose monitor.	Your local hospital or research centre.<br><br>Approximately 1 hour
**Study Visit 3**<br><br>*12 weeks into the study*	Your child will have a physical exam and be asked questions about your health. We will also need a urine sample and a blood sample (up to 59.5ml which is nearly 3 and a half tablespoons).<br><br>Next your child will receive an injection of either the study medicine, Ustekinumab or placebo, depending on which treatment group they are in.<br><br>We will also download the data stored on their blood glucose monitor.	Your local hospital or research centre.<br><br>Approximately 1 hour
**Study Visit 4**<br><br>*20 weeks into the study*	At this visit your child will receive an injection of either the study medicine or placebo. They will also have a urine test but no blood sample will be needed.<br><br>This appointment may be done at your home.	Your local hospital or research centre or your home.<br><br>Approximately 1 hour
**Study Visit 5**<br><br>*28 weeks into the study*	Your child will be asked to do a second milkshake test in exactly the same way as described earlier and will involve taking less than 1 tablespoon (10ml) of blood over a 2 hour period. This means that they have to arrive fasted for this study visit.<br><br>Your child will also have a physical examination, and be asked questions about their health. We will also need a urine sample and another blood sample (up to 61.5ml which is 3 and a half tablespoons).<br><br>Then your child will receive an injection of either the study medicine, Ustekinumab or placebo, depending on which treatment group they are in.<br><br>This visit will also include both you and your child completing a short questionnaire exactly like the one you did at the screening visit.<br><br>We will also download the data stored on their blood glucose monitor.	Your local hospital or research centre.<br><br>Approximately 3 hours

Gregory JW, *et al. BMJ Open* 2021; 11:e049595. doi: 10.1136/bmjopen-2021-049595

**Study Visit 6**<br>36 weeks into the study	At this visit your child will receive an injection of either the study medicine or placebo. They will also have a urine test but no blood sample will be needed.<br>This appointment may be done at your home.	Your local hospital or research centre or your home.<br>Approximately 1 hour
**Study Visit 7**<br>44 weeks into the study	At this visit your child will receive the FINAL injection of either the study medicine or placebo. They will also have a urine test but no blood sample will be needed.<br>This appointment may be done at your home.<br>We will also download the data stored on their blood glucose monitor.	Your local hospital or research centre or your home.<br>Approximately 1 hour
**Study Visit 8 – follow up**<br>52 weeks into the study	At this final visit your child will have a physical examination and be asked questions about their health. They will be asked to do a final milkshake test in exactly the same way as described earlier and will involve taking half a tablespoon (10ml) of blood over a 2 hour period.<br>We will also need a urine sample and another blood sample (up to 61.5ml which is 3 and a half tablespoons).<br>This visit will also include both you and your child completing a short questionnaire exactly like the one you did at the screening visit.<br>We will also download the data stored on their blood glucose monitor.	Your local hospital or research centre.<br>Approximately 3 hours
**Remote follow up**<br>Weeks 78 & 104	We will check your child's hospital records to find out how they are doing. They do not need to come into the hospital for a visit. We may need to call you up at home to check that they are OK and in good health.	No visit needed

WHAT ELSE WILL MY CHILD AND I BE ASKED TO DO?

As well as attending with your child to your local hospital or research centre for study visits there are a few other things we will ask your child to do during the study which may involve your help:

- Your child will be asked to complete a diary between study visits to record:
  a) How much insulin they take during the study (for the two weeks before every study visit).
  b) If they feel or have been unwell or have to take any other medicines during the study.
  c) If they have any hypoglycaemic (low blood glucose levels) episodes that need treating.

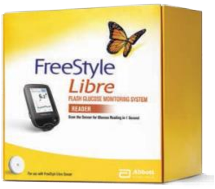

- We will ask them to test their blood glucose levels at home for at least 2 weeks before each visit using our FREE Abbott Freestyle Libre glucose monitoring system so they don't need to do extra finger prick tests for the trial. They will need to wear a sensor on their arm for the two weeks before our study visit if they want to be in the trial. We will show you both how it works. They are free to use the monitor at home for the rest of the time of study if they want to. We will give them enough sensors to allow this for a year.

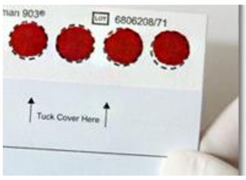

- We will ask them to give a blood spot sample which they can do at home. They will need to do this once a week for 28 weekly, then every month for the next 6 months. The test involves pricking their finger like a normal finger prick blood glucose test and dabbing the blood spot into a special card. They will need to do this before the first meal of the day and then 1 hour later. The paper should be posted to a special laboratory for testing. We will show you both how to do this and will provide envelopes and pay for the postage.

You will be asked to make sure that your child does not have certain vaccinations before, during and immediately after the study. If they need a vaccination, for example if they are travelling abroad, you must tell the study doctor or nurse immediately.

Please be aware that a urine pregnancy test will be done for all females at each study visit. We need to do this

Gregory JW, *et al. BMJ Open* 2021; 11:e049595. doi: 10.1136/bmjopen-2021-049595

because the law requires us to do this in clinical trials because the effects of the study medicine are not known in pregnancy and we want the mother and baby to be safe. Any confirmed pregnancies will be monitored closely with your permission (including the female partners of male participants). Rather than asking if your child is engaged in actions that may lead to pregnancy, we will ask everybody (males and females) to agree to use adequate contraception (hormonal based contraception, barrier contraception, abstinence) until 4 months following the date of their final treatment. You will need to agree to this on behalf of your child if you want them to take part. Your GP or a pharmacist can advise on suitable contraception for your child.

Finally, we would like you to complete a short questionnaire about your child's health and diabetes. These questionnaires will be done at the second screening visit and study visits 5 and 8. It is important that the person who consents will complete all three questionnaires.

WILL THE STUDY HELP MY CHILD?

If your child has been allocated to the group that receives the study medicine, Ustekinumab, it is possible that it will help their pancreas make insulin for longer. However we cannot say this for certain until we have completed this study. During the study your child's diabetes will be very closely monitored. This will include regular check-ups with your local diabetes team including routine blood testing. You and your child will have more time with the research team to discuss their diabetes and ask questions than at a normal clinic appointment.

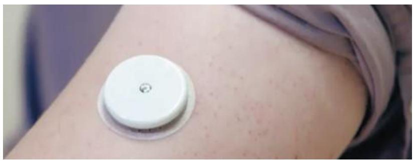

Your child will be provided with a FREE Abbott Freestyle Libre blood glucose monitoring system. They can use this to check their blood glucose levels while they are in the study, although they will still need to do some finger prick tests. The treatments will stop at week 44 and your child will not receive any further injections of the study drug / placebo during the trial.

WHAT HAPPENS WHEN THE STUDY STOPS?

We will collect all the information together and we will decide if the study medicine can help people with Type 1 Diabetes make their own insulin for longer. If it does then we will carry out a bigger version of this study. You and your child will be informed which treatment they were given and their medical records will be updated with the treatment information.

WHAT IF NEW INFORMATION COMES ALONG?

Sometimes during research, we get new information about the treatment being studied. If this happens, we will tell you about it and discuss whether you and your child want to continue in the study.

WHAT IF I MY CHILD DOES NOT WANT TO TAKE PART ANYMORE?

Just let your study doctor or research nurse know about your decision. You and your child will be asked whether you wish to withdraw from just having the study treatment or from the whole study (including the study visits and data collection). You can withdraw from treatment but still come to study visits for sample and data collection. If they want to withdraw completely, we will make a note of this and we will make sure that your child are transferred back to normal care as quickly as possible.

WHAT IF THERE IS A PROBLEM OR SOMETHING GOES WRONG?

If your child feels unwell or suffers any unusual discomfort during the study it is important to inform the study doctor or nurse as soon as possible. If it is because of something in the study, we need to consider stopping your child's treatment.

If you or your child feel overwhelmed by their recent diagnosis, you can call the local [title] on [tel number / email] and they can talk through any concerns with you both.

If you are unhappy about the conduct of the study and wish to complain, you can do this through:

(name and contact details of appropriate organisations – site specific).

WHAT ARE THE POSSIBLE SIDE EFFECTS FOR MY CHILD IN PARTICIPATING IN THE STUDY?

They may get a bruise or a little discomfort at the site of the blood tests.

The FreeStyle Libre sensor may cause a slight rash for some people who might be allergic to the adhesive on the

Gregory JW, et al. BMJ Open 2021; 11:e049595. doi: 10.1136/bmjopen-2021-049595

sensor. The manufacturer are always improving their sensors to stop this happening but your child may experience some discomfort from wearing the sensor. Please let the research team know if this happens.

During the Milkshake test, they may experience changes in blood glucose level because they will not have taken insulin immediately beforehand. The study nurses and doctors will be available to help your child make any changes to their usual insulin doses after this test.

The medicine (Ustekinumab) being used in this study is currently used in patients with skin and bowel conditions. Because the medicine acts on the immune system, there is a possibility that it will increase the risk of infections and cancer, but so far, this has not been found to be a problem with people treated with this medicine for others diseases. It is also possible that they may get an allergic reaction to the treatment injection. We will ask them to stay for one hour after their first injection to check for any reactions.

It is routine to check people who receive this medicine for tuberculosis (TB) as, if your child have this infection, the study medicine may make it worse. We will check your child and if there is evidence of TB infection, they will not be allowed to take part.

If your child takes part in this study, they will have a chest X-ray to rule out TB which is additional to standard care. Chest x-rays involve using ionising radiation to form images of the body. Ionising radiation can cause cell damage in the longer term which can sometimes lead to cancer developing. However, we only ask for one x-ray so taking part in this study will add only a very small chance of this happening to them when they are older. The risk is not much greater than that found with natural background radiation.

If your child feels ill at any time during the trial and you go to your GP or the hospital, please show them the membership card your child will be given so that they can contact the research team to ask about possible side effects.

The research team will carefully monitor your child throughout the study to check their health and to ensure that they are not experiencing any side effects. You must tell someone straight away if they complain that they feel unwell.

WILL MY CHILD RECEIVE ANY PAYMENT FOR TAKING PART?

Your child will receive a £10.00 gift voucher for each treatment visit and we will give them £30.00 gift if they come to the final visit (visit 8) (that's £100 in total if they come to all visits).

You will be able to claim back your travel expenses for getting your child to the local hospital or research centre for all screening, treatment and follow up visits.

WHAT INFORMATION WILL YOU COLLECT AND HOW WILL IT BE KEPT PRIVATE?

We will ask for your name and contact details as well as your child's name so that the research nurse can keep in touch and manage your visits. We will also need to collect data about your child's health which we get from their tests, their blood glucose monitor and their medical records. We will also ask you and your child to complete a questionnaire each at three time points.

The people in our research team at the local hospital or research centre will know that your child is taking part. The doctors looking after your child when you come to hospital will also know that they are in the study. Their medical notes may be looked at by staff from Swansea and Cardiff Universities or NHS and regulatory auditors who will be checking that the study is being done correctly. If you agree, we will also tell your family doctor (GP) that they are in the study.

People at the research laboratories will not know who your child is when they test their samples. Your child will be given a study number to replace their name so any study samples and data related to them will be anonymised. The questionnaires and the data from your flash glucose monitor will also use study numbers instead of names.

All information collected about your child during the study will be kept by the research nurse in a locked cabinet and entered onto a secure database. Only people with the password can open up the database.

Cardiff University is the sponsor for this study based in the United Kingdom. Cardiff University will be using information from your child and their medical records in order to undertake this study and will act as the data controller for this study. This means that we are responsible for looking after their information and using it properly. Cardiff University will keep identifiable information about them for 25 years after the study has finished. Your rights to access, change or move the information about your child are limited, as we need to manage their information in specific ways in order for the research to be reliable and accurate. If you/they withdraw from the study, we will keep

Gregory JW, *et al. BMJ Open* 2021; 11:e049595. doi: 10.1136/bmjopen-2021-049595

the information about them that we have already obtained. To safeguard their rights, we will use the minimum personally-identifiable information possible. You can find out more about how we use your child's information by contacting inforequest@cardiff.ac.uk.

WHAT WILL HAPPEN TO ANY SAMPLES THEY GIVE?

We want to test your child's blood and urine to better understand your diabetes and how the study medicine or placebo affects their diabetes. The blood samples will be used to test:

1. The amount of insulin their body still makes before and after taking part in the study.
2. Their average blood glucose levels
3. The antibodies to the insulin making cells
4. General health checks – anaemia, kidneys, liver etc
5. How much of the study medication is in their blood.
6. How their body's immune system is reacting to the study medication.

Scientists in laboratories around the UK will look at their anonymised blood samples. These samples will be stored in a safe place. Some of their blood samples will be sent to a laboratory in Europe and either America or Canada for special testing to find out how much of the study medicine is present.

We would like to keep any leftover blood samples in a special tissue repository permanently - we will ask for your permission to do this. The samples will only be accessed by scientists who have special permission to do so. The samples might be sent outside the UK to other research teams in Europe or countries such as America and Canada if you agree to this. These other teams must have permission from us to use your sample before we send it. If you do not want to agree to this, their samples will be destroyed after they have been analysed.

WILL ANY GENETIC TESTS BE DONE?

We will use their blood samples to help us study the genes involved with diabetes and the immune system. These samples will not have your name on them and will not be used for any other reason without your permission.

WHAT WILL HAPPEN TO THE RESULTS OF THIS STUDY?

The full results of this study will not be known until the last patient has completed their tests, which may take more than 5 years. The research results will be reported in scientific publications and meetings but you will not be identified by name at all. If you are interested in receiving a summary of the research results, we can arrange this.

WHO IS ORGANISING AND FUNDING THE STUDY?

CARDIFF UNIVERSITY PRIFYSGOL CAERDYDD

Swansea University Prifysgol Abertawe
Swansea Trials Unit Uned Dreialon Abertawe

The study is being organised by researchers at Cardiff University and Swansea University.

It is being funded by a grant from the National Institute for Health and Research (NIHR).

NHS National Institute for Health Research

WHERE IS THE STUDY BEING DONE?

The study is being done at hospitals and research centres across England, Wales and Scotland.

WHO HAS CHECKED THIS STUDY?

Before any research goes ahead it has to be checked by a Research Ethics Committee. This is a group of people who make sure that the research is OK to do and to make sure that the patient will be safe. This study has been looked at by Wales REC 3. As this study is looking at a medicine, it has also been approved by the government's Medicine and Healthcare products Regulatory Authority (MHRA) who check that the researchers carry out the study safely. It has also been checked by national and local NHS organisations to make sure that the study can be done using their site and staff.

WHAT SHOULD I DO NOW?

If you are interested in taking part, or have any questions please contact one of the following people:

Name:	Name:	Name: (only if needed)

**Role:** Principal Investigator<br>**Tel. No:**<br>**Email:**	**Role:** Research nurse<br>**Tel. No:**<br>**Email:**	**Role:**<br>**Tel. No:**<br>**Email:**

Alternatively, you may want to speak to someone at the USTEKID Trial Office who are managing the study, based at Swansea University. The Trial Manager's details are below:

Name: Dr Kym Thorne
Tel. No: 01792 606372 (direct) or 01792 606545 for Swansea Trials Unit
Email: ustekid@swansea.ac.uk
Address: Floor 2, Institute of Life Sciences 2, Swansea University Medical School, Singleton Park, Swansea SA2 8PP

Thank you for taking the time to read this information sheet and for considering taking part in this research study

Gregory JW, *et al. BMJ Open* 2021; 11:e049595. doi: 10.1136/bmjopen-2021-049595
