# Supplementary material for: Phase II multicentre, double-blind, randomised trial of ustekinumab in adolescents with new-onset type 1 diabetes (USTEK1D): trial protocol
Source: BMJ Open. 2021 Oct 18;11(10):e049595. doi: 10.1136/bmjopen-2021-049595 (PMC8524290; doi:10.1136/bmjopen-2021-049595)
Supplement: Supplementary data [file bmjopen-2021-049595supp003.pdf]

[Insert local headers]

USTEKID

NHS National Institute for Health Research

Cardiff University Prifysgol Caerdydd

Swansea University Prifysgol Abertawe Swansea Trials Unit Uned Dreialon Abertawe

A research study to see if the medicine Ustekinumab can make diabetes easier to manage

CONTACT DETAILS FOR STUDY TEAM:

NURSE:

DOCTOR:

EMERGENCIES:

FOR YOUNG PEOPLE AGED 16-18 YEARS OLD

We would like you to help us with our research study. Please read this information carefully and talk to your parent or carer about the study. Ask us if there is anything that is not clear or if you want to know more. Take time to decide if you want to take part. It is up to you if you want to do this. If you do not, then that is fine, you will be looked after by your doctors just the same.

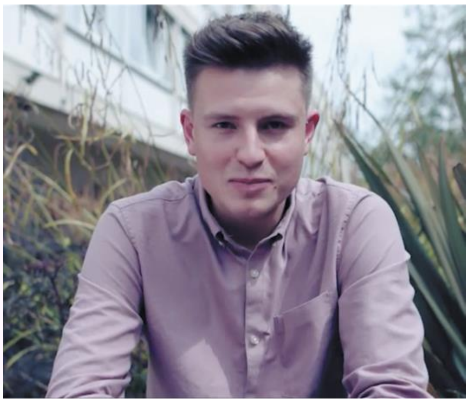

Please look at our video explaining the trial at www.type1diabetesresearch.org.uk/current-trials. The blue box below and the video contains the key points about the study. If you would like to know more, please read the rest of this leaflet.

KEY POINTS ABOUT THE STUDY:

- We want to see if the study medicine, **Ustekinumab**, can make Type 1 Diabetes (the type you have) easier to manage. The medicine works by "protecting" some of the cells in the pancreas that still produce insulin from attack by the immune system.
- The study will involve you having an injection every 1-3 months with either the study medicine, Ustekinumab or a placebo (a "dummy medicine"). These injections are given under the skin just like insulin injections and will be done by the study doctor or nurse. Neither you nor the research team will know if you receive the study medicine or the placebo.
- The medicine is already being used to treat other illnesses quite safely. We will give you information about possible side effects before you decide if you will take part.
- You will be asked to come into your local hospital or research centre for 10 study visits over a 15 month period, but where possible this will be on the same day as your routine hospital visits. The first two will check if you are eligible to take part. Three of these visits can sometimes be done by a research nurse at home.
- We will ask you to provide extra blood and urine samples to check that it is safe for you to take part in the study and to check your health, your blood glucose levels and how your immune system is working during the study.
- At three visits, you will have blood tests over a 2 hour period to see how much insulin your body is making.
- We will provide you with a glucose monitor (Freestyle Libre) to wear for 2 weeks before each visit. You can keep the monitor for use at home for the whole time of the study.
- You will be offered a small gift voucher for each visit you come to and your travel expenses will be paid.
- You can stop taking part in the study at any time and you do not have to give a reason why.

Gregory JW, *et al. BMJ Open* 2021; 11:e049595. doi: 10.1136/bmjopen-2021-049595

WHY IS THIS STUDY BEING DONE?

This study is being done to see if a medicine called **Ustekinumab** can help to "protect" the cells in the body that produce insulin in young people recently diagnosed with Type 1 Diabetes. It is caused by the body's own immune system damaging the cells in the pancreas that make insulin. Our aim is to develop a treatment that can slow this process by targeting the immune cells causing the damage.

At the time of diagnosis, most people your age have 10-20% of their insulin-producing cells still working. It usually takes between 1 and 5 years before they stop working completely. Sometimes these last few working cells can make enough insulin to make blood glucose levels stable and easier to control – this is called the "Honeymoon period". This period is only temporary and doesn't last. Ustekinumab, the study medicine, may make this period last longer by reducing the damaging effects of the immune system on the remaining insulin-producing cells in the pancreas.

Ustekinumab is currently given to adults and teenagers with particular skin and bowel problems and it is known to be safe to use and effective at treating those conditions.

WHY HAVE I BEEN ASKED TO TAKE PART?

You have been chosen because you are aged 12 - 18 years old and have recently been diagnosed with Type 1 diabetes.

DO I HAVE TO TAKE PART?

No. It is completely up to you whether or not you take part and you can always change your mind at any time. If you are interested in this study then:

- Let us know by calling one of the people listed at the end of this information sheet.
- A member of the research team will contact you to explain more about the study and answer any questions you have.
- If you agree, you will sign a consent form and be given a copy of your signed consent form and this information sheet to keep.

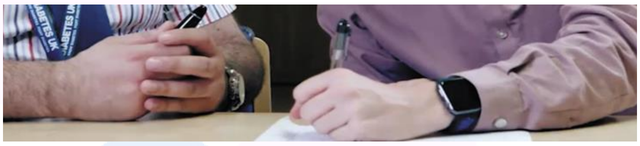

If you make a decision to take part, ***you are still free to withdraw from the study at any time without giving a reason***. This will not affect the medical care you get from your diabetes doctor in any way.

You do not need to decide if you want to take part straight away. You can take your time and talk about the study with your family, friends and the study team if you want to. You need to let us know within 6 weeks of being diagnosed with diabetes, so that we can start the treatment early enough.

WHAT HAPPENS ONCE I HAVE AGREED TO TAKE PART?

VISIT	WHAT WILL HAPPEN	WHERE AND HOW LONG WILL IT TAKE?
Screening visits	Before we can start the treatment, we need to check that you are eligible to take part in the study - this is called "screening". You will be asked to come to your local hospital or research centre to talk about the study. This is where you will be able to ask questions.<br>If you are happy to take part you will be asked to sign a consent form before we begin the screening tests which involve:<br>Screening visit 1:<br>• Doing a general health check (this includes a general examination and measuring your height, weight and blood pressure).<br>• Asking about any medicine you are taking and any illnesses you have had or still have.<br>• Taking some blood samples (between 0.5 - 2 tablespoons) from your arm to check your general health and diabetes, as well as testing for infections such as TB (tuberculosis), hepatitis and HIV. Blood volumes vary according to your hospital's local testing procedures.	Your local hospital or research centre.<br>Approximately 1 hour for the first visit and 3 hours for the second visit.

<table>
<tr>
<td></td>
<td>

Taking a chest X-ray to test for TB. There will be one other test for TB which will either be a blood test or a Mantoux test (a skin reaction test), depending on what your hospital's local procedures are.
Taking a urine sample to test for infection, kidney function and a routine pregnancy test for all girls.

If one of these tests tells us that it is not safe for you to take part, we will let you know straight away. If the tests are OK, then we will proceed with a second screening visit for a few more tests.

Screening visit 2:

Doing a general health check.
Asking about any medicine you are taking and any illnesses you have had or still have since your last visit.
Taking a urine sample to test for infection, kidney function and a routine pregnancy test for all girls.
You and your parent/carer will be asked to complete a short questionnaire about your diabetes and general health.
You will be given a free blood glucose monitor (FreeStyle Libre) and sensors to use for the study. The sensor should be worn two weeks prior to every study visit but can be worn constantly if you find it helpful.
A Mixed Meal Tolerance Test (or Milkshake test). This test tells us how much insulin your body is still making. During this test we also take additional blood samples to test the immune system and pancreas function.

On the day of the Mixed Meal Tolerance Test, you will need to make sure that you have not eaten or drunk anything except water from midnight the night before onwards. You will also be asked to not take your early morning short acting insulin because you will not be eating breakfast. You will need to tell us your blood glucose levels on waking so that we can make sure it's OK for you to be tested. It will need to be between 4.0 and 11.1mmol/L for the screening visit to happen. If it is lower, the test will be rescheduled but if it is higher, you may be advised to take short acting insulin so that the visit can go ahead.

.

At the hospital, you will have blood taken through a small plastic tube (cannula) which we will insert in your arm (using local anaesthetic cream/spray if you want it). This will stay in your arm during the test so that we can take blood samples more easily.

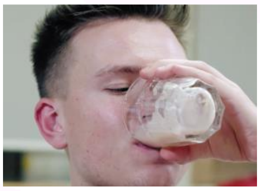

Then you will be given a milkshake to drink (various flavours available). The research doctor or nurse will take blood at fixed times over the next 2 hours. Over this time, less than 1 tablespoon (10ml) of blood will be taken from you in total. During this time, you can relax on a bed, play games, read or study. Once the test is completed we will give you something to eat and drink and you will receive insulin in whatever dose is needed.

An additional 40mls (less than 3 tablespoons) will be taken at the same visit for testing in our laboratories.

Your blood samples will be analysed within 2 weeks and if the test shows that you are still making some of your own insulin, you will be contacted by the research team to tell you that everything is OK to be part of the study and to arrange the first injection.

If it is not convenient to have two separate visits for testing, we can arrange to combine the two sets of tests if you let us know beforehand. This is because the combined screening visit needs you to be fasted on arrival.
</td>
<td></td>
</tr>
<tr>
<td>Study Visit 1</td>
<td>If you take part in this study you will be randomly allocated to either the study medicine group or the placebo (a "dummy" medicine that has no effect) group. This is decided by chance using a computer programme before the study visit and neither you nor the research team will know until the end of the study what treatment you have received.

2 out of every 3 people taking part will receive the study medicine compared with only 1 out of 3 getting the placebo. This is to give people a better chance of getting the study medicine.</td>
<td>Your local hospital or research centre.

Approximately 2 hours.</td>
</tr>
</table>

	Treatment visits will be booked in so that you receive your injection at the required intervals. The second dose will be four weeks after the first. All other doses afterwards will be eight weeks apart. These will be booked in advance so that any issues with attendance can be identified as soon as possible (e.g. holidays, exams). Postponing a treatment visit may result in the treatment being stopped if too much time has passed because the levels of the study medicine in your body may go too low and won't work anymore.<br>You will have a physical examination and tests will be done on your urine and blood samples (up to 57.5ml which is about 3 and a half tablespoons) at this visit.<br>Then you will receive an injection of either the study medicine, Ustekinumab, or the placebo. Injections are given under the skin using a very small needle similar to the one that you already use for daily insulin injections.<br>You will be asked to stay in the hospital for 1 hour after you receive the injection so that the study team can make sure that there are no side effects and that you are safe to leave.	
**Study Visit 2**<br>*4 weeks after the 1st visit*	You will have a physical examination and be asked questions about your health. We will also need a urine sample and a blood sample (up to 50.5ml which is just under 3 tablespoons). Then you will receive an injection of either the study medicine, Ustekinumab or placebo, depending on which treatment group you are in.<br>We will also download the data stored on your blood glucose monitor.	Your local hospital or research centre.<br>Approximately 1 hour
**Study Visit 3**<br>*12 weeks into the study*	You will have a physical exam and be asked questions about your health. We will also need a urine sample and a blood sample (up to 59.5ml which is nearly 3 and a half tablespoons).<br>Next you will receive an injection of either the study medicine, Ustekinumab or placebo, depending on which treatment group you are in.<br>We will also download the data stored on your blood glucose monitor.	Your local hospital or research centre.<br>Approximately 1 hour
**Study Visit 4**<br>*20 weeks into the study*	At this visit you will receive an injection of either the study medicine or placebo. You will also have a urine test but no blood sample will be needed.<br>This appointment can be done at your home.	Your local hospital or research centre or your home.<br>Approximately 1 hour
**Study Visit 5**<br>*28 weeks into the study*	You will be asked to do a second milkshake test in exactly the same way as described earlier and will involve taking half a tablespoon (10ml) of blood over a 2 hour period. This means that you have to arrive fasted for this study visit.<br>You will also have a physical examination, and be asked questions about your health. We will also need a urine sample and another blood sample (up to 61.5ml which is 3 and a half tablespoons).<br>Then you will receive an injection of either the study medicine, Ustekinumab or placebo, depending on which treatment group you are in.<br>This visit will also include both you and your parent/carer completing a short questionnaire exactly like the one you did at the screening visit.<br>We will also download the data stored on your blood glucose monitor.	Your local hospital or research centre.<br>Approximately 3 hours
**Study Visit 6**<br>36 weeks into the study	At this visit you will receive an injection of either the study medicine or placebo. You will also have a urine test but no blood sample will be needed.<br>This appointment can be done at your home.	Your local hospital or research centre or your home.<br>Approximately 1 hour
**Study Visit 7**<br>44 weeks into the study	At this visit you will receive the <u>FINAL</u> injection of either the study medicine or placebo. You will also have a urine test but no blood sample will be needed.<br>This appointment can be done at your home.<br>We will also download the data stored on your blood glucose monitor.	Your local hospital or research centre or your home.<br>Approximately 1 hour
**Study Visit 8 – follow up**	At this final visit you will have a physical examination and be asked questions about your health. You will be asked to do a final milkshake test in exactly the same way as described earlier and will involve taking half a tablespoon (10ml) of blood over a 2 hour period.	Your local hospital or research centre.

52 weeks into the study	We will also need a urine sample and another blood sample (up to 61.5ml which is 3 and a half tablespoons).<br>This visit will also include both you and your parent/carer completing a short questionnaire exactly like the one you did at the screening visit.<br>We will also download the data stored on your blood glucose monitor.	Approximately 3 hours
**Remote follow up**<br>Weeks 78 & 104	We will check your hospital records to find out how you are doing. You do not need to come into the hospital for a visit. We may need to call you up at home to check that you are OK and in good health.	No visit needed

WHAT ELSE WILL I BE ASKED TO DO?

As well as coming to your local hospital or research centre for study visits there are a few other things we will ask you to do during the study:

- You will be asked to complete a diary between study visits to record:
  a) How much insulin you take during the study (for the <u>two weeks before every study visit</u>)
  b) If you feel or have been unwell or have to take any other medicines during the study.
  c) If you have any hypoglycaemic (low blood glucose levels) episodes that need treating.

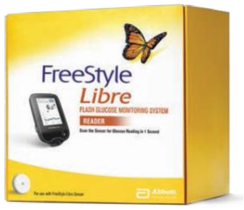

- We will ask you to test your blood glucose levels at home for at least 2 weeks before each visit using our FREE Abbott Freestyle Libre flash glucose monitoring system so you don't need to do extra finger prick tests for the trial. You will need to wear a sensor on your arm for the two weeks before our study visit if you want to be in the trial. We will show you how it works. You are free to use the monitor at home for the rest of the time of study if you want to. We will give you enough sensors to allow this for a year.

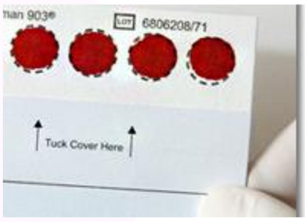

- We will ask you to give blood spot samples which you can do at home. You will need to do this once a week for 28 weeks, then every month for the next 6 months. The test involves pricking your finger like a normal finger prick blood glucose test and dabbing the blood spot onto a special card. You will need to do this before the first meal of the day and then 1 hour later. The card should be posted to our special laboratory for testing. We will show you how to do this and will provide envelopes and pay for the postage.
- You will be asked to make sure that you do not have certain vaccinations before, during and immediately after the study. If you need a vaccination, for example if you are travelling abroad, you must tell the study doctor or nurse immediately.
- Please be aware that a urine pregnancy test will be done for all females at each study visit. We need to do this because the law requires us to do this in clinical trials because the effects of the study medicine are not known in pregnancy and we would want the mother and baby to be safe. Any confirmed pregnancies will be monitored closely with your permission (including the female partners of male participants).
- Rather than asking if you are engaged in actions that may lead to pregnancy, we will ask <u>everybody</u> to agree to use adequate contraception (hormonal based contraception, barrier contraception, abstinence) until 4 months following the date of their final treatment. All participants will need to agree to this to take part. Your GP or a pharmacist can advise on suitable contraception if you don't want to discuss this with your parents/carers.
- Finally, we would like you to complete a short questionnaire about your health and diabetes. These questionnaires will be done at the second screening visit and study visits 5 and 8. Your parent/carer will also be asked to complete questionnaires at these time points so we will ask that they agree to attend those study visits with you.

WILL THE STUDY HELP ME?

If you have been allocated to the group that receives the study medicine, Ustekinumab, it is possible that it will help your pancreas make insulin for longer. However, we cannot say this for certain until we have completed this study.

Gregory JW, *et al. BMJ Open* 2021; 11:e049595. doi: 10.1136/bmjopen-2021-049595

During the study your diabetes will be very closely monitored. This will include regular check-ups with your local diabetes team including routine blood testing. You will have more time with the research team to discuss your diabetes and ask questions than at a normal clinic appointment.

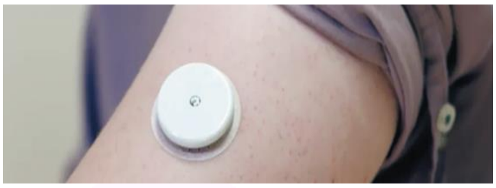

You will be provided with a FREE Abbott Freestyle Libre flash glucose monitoring system. You can use this to check your blood sugar levels while you are in the study, although you will still need to do some finger prick tests. The treatments will stop at week 44 and you will not receive any further injections of the study drug / placebo during the trial.

WHAT HAPPENS WHEN THE STUDY STOPS?

We will collect all the information together and we will decide if the study medicine can help people with Type 1 Diabetes make their own insulin for longer. If it does then we will carry out a bigger version of this study.

You will be informed which treatment you were given and your medical records will be updated with the treatment information.

WHAT IF NEW INFORMATION COMES ALONG?

Sometimes during research, we get new information about the treatment being studied. If this happens, we will tell you about it and discuss whether you want to continue in the study.

WHAT IF I DO NOT WANT TO TAKE PART ANYMORE?

Just let your study doctor or research nurse know about your decision. You will be asked whether you wish to withdraw from just having the study treatment or from the whole study (including the study visits and data collection).

You can withdraw from treatment but still come to study visits for sample and data collection. If you want to withdraw completely, we will make a note of this and we will make sure that you are transferred back to normal care as quickly as possible.

WHAT IF THERE IS A PROBLEM OR SOMETHING GOES WRONG?

If you feel unwell or suffer any unusual discomfort during the study it is important to inform the study doctor or nurse as soon as possible. If it is because of something in the study, we need to consider stopping your treatment. The diabetes care team will also be informed.

If you feel overwhelmed by your recent diagnosis, you can call the local [title] on [tel number / email] and they can talk through your concerns with you.

If you are unhappy about the conduct of the study and wish to complain, you can do this through:

(name and contact details of appropriate organisations – site specific).

WHAT ARE THE POSSIBLE SIDE EFFECTS FOR ME IN PARTICIPATING IN THE STUDY?

You may get a bruise or a little discomfort at the site of the blood tests.

The FreeStyle Libre sensor may cause a slight rash for some people who might be allergic to the adhesive on the sensor. The manufacturer are always improving their sensors to stop this happening but you may experience some discomfort from wearing the sensor. Please let the research team know if this happens.

During the Milkshake test, you may experience changes in blood glucose level because you will not have taken insulin immediately beforehand. The study nurses and doctors will be available to help you make any changes to your usual insulin doses after this test.

The medicine (Ustekinumab) being used in this study is currently used in patients with skin and bowel conditions safely. Because the medicine acts on the immune system, there is a possibility that it will increase the risk of infections and cancer, but so far this has not been found to be a problem with people treated with this medicine for others diseases. It is also possible that you may get an allergic reaction to the treatment injection. We will ask you to stay for one hour after your first injection to check for any reactions.

It is routine to check people who receive this medicine for tuberculosis (TB) as if you have this infection the study medicine may make it worse. If there is evidence of TB infection, you will not be allowed to take part.

If you take part in this study, you will have a chest X-ray to rule out TB which is additional to standard care. Chest x-rays involve using ionising radiation to form images of the body. Ionising radiation can cause cell damage in the

Gregory JW, et al. BMJ Open 2021; 11:e049595. doi: 10.1136/bmjopen-2021-049595

longer term which can sometimes lead to cancer developing. However, we only ask for one x-ray so taking part in this study will add only a very small chance of this happening to you when you are older. The risk is not much greater than that found with natural background radiation.

If you feel ill at any time during the trial and go to your GP or the hospital, please show them the membership card you will be given so that they can contact the research team to ask about possible side effects.

The research team will carefully monitor you throughout the study to check your health and to ensure that you are not experiencing any side effects. You must tell someone straight away if you feel unwell.

WILL I RECEIVE ANY PAYMENT FOR TAKING PART?

You will receive a £10.00 gift voucher for each treatment visit and we will give you £30.00 gift if you come to the final visit (visit 8) (that's £100 in total if you come to all visits).

You will be able to claim back your travel expenses for getting to the local hospital or research centre for all screening, treatment and follow up visits.

WHAT INFORMATION WILL YOU COLLECT AND HOW WILL IT BE KEPT PRIVATE?

We will ask for your name and contact details so that the research nurse can keep in touch and manage your visits. We will also need to collect data about your health that we get from your tests, your flash glucose monitor and your medical records. We will also ask to complete a questionnaire at three time points.

The people in our research team at the local hospital or research centre will know that you are taking part. The doctors looking after you when you come to hospital will also know that you are in the study. Your medical notes may be looked at by staff from Swansea and Cardiff Universities or NHS and regulatory auditors who will be checking that the study is being done correctly. If you agree, we will also tell your family doctor (GP) that you are in the study.

People at the research laboratories will not know who you are when they test your samples. You will be given a study number to replace your name so any study samples and data related to you will be anonymised. The questionnaires and the data from your flash glucose monitor will also use a study number instead of your name.

All information collected about you during the study will be kept by the research nurse in a locked cabinet and entered onto a secure database. Only people with the password can open up the database.

Cardiff University is the sponsor for this study based in the United Kingdom. Cardiff University will be using information from you and your medical records in order to undertake this study and will act as the data controller for this study. This means that we are responsible for looking after your information and using it properly. Cardiff University will keep identifiable information about you for 25 years after the study has finished.

Your rights to access, change or move your information are limited, as we need to manage your information in specific ways in order for the research to be reliable and accurate. If you withdraw from the study, we will keep the information about you that we have already obtained. To safeguard your rights, we will use the minimum personally-identifiable information possible.

You can find out more about how we use your information by contacting inforequest@cardiff.ac.uk.

WHAT WILL HAPPEN TO ANY SAMPLES I GIVE?

We want to test your blood and urine to better understand your diabetes and how the study medicine or placebo affects your diabetes. The blood samples will be used to test:

1. The amount of insulin your body still makes before and after taking part in the study.
2. Your average blood glucose levels
3. The antibodies to the insulin making cells
4. General health checks – anaemia, kidneys, liver etc
5. How much of the study medication is in your blood.
6. How your body's immune system is reacting to the study medication.

Scientists in laboratories around the UK will look at your anonymised blood samples. These samples will be stored in a safe place. Some of your blood samples will be sent to a laboratory in Europe and either America or Canada for special testing to find out how much of the study medicine is present.

We would like to keep any leftover blood samples in a special tissue repository permanently - we will ask for your permission to do this. The samples will only be accessed by scientists who have special permission to do so. The samples might be sent outside the UK to other research teams in Europe or countries such as America and Canada if you agree to this. These other teams must have permission from us to use your sample before we send it. If you

Gregory JW, et al. BMJ Open 2021; 11:e049595. doi: 10.1136/bmjopen-2021-049595

do not want to agree to this, your samples will be destroyed after they have been analysed.

WILL ANY GENETIC TESTS BE DONE?

We will use your blood samples to help us study the genes involved with diabetes and the immune system. These samples will not have your name on them and will not be used for any other reason without your permission.

WHAT WILL HAPPEN TO THE RESULTS OF THIS STUDY?

The full results of this study will not be known until the last patient has completed their tests, which may take more than 5 years. The research results will be reported in scientific publications and meetings but you will not be identified by name at all. If you are interested in receiving a summary of the research results, we can arrange this.

WHO IS ORGANISING AND FUNDING THE STUDY?

The study is being organised by researchers at Cardiff University and Swansea University.

It is being funded by a grant from the National Institute for Health and Research (NIHR).

WHERE IS THE STUDY BEING DONE?

The study is being done at hospitals and research centres across England, Wales and Scotland.

WHO HAS CHECKED THIS STUDY?

Before any research goes ahead it has to be checked by a Research Ethics Committee. This is a group of people who make sure that the research is OK to do and to make sure that the patient will be safe. This study has been looked at by Wales REC 3. As this study is looking at a medicine, it has also been approved by the government's Medicine and Healthcare products Regulatory Authority (MHRA) who check that the researchers carry out the study safely. It has also been checked by national and local NHS organisations to make sure that the study can be done using their site and staff.

WHAT SHOULD I DO NOW?

If you are interested in taking part, or have any questions please contact one of the following people:

**Name:**	**Name:**	**Name:** (only if needed)
**Role:** Principal Investigator	**Role:** Research nurse	**Role:**
**Tel. No:**	**Tel. No:**	**Tel. No:**
**Email:**	**Email:**	**Email:**

Alternatively, you may want to speak to someone at the USTEKID Trial Office who are managing the study, based at Swansea University. The Trial Manager's details are below:

Name: Dr Kym Thorne
Tel. No: 01792 606372 (direct) or 01792 606545 for Swansea Trials Unit
Email: ustekid@swansea.ac.uk
Address: Floor 2, Institute of Life Sciences 2, Swansea University Medical School, Singleton Park, Swansea SA2 8PP

Thank you for taking the time to read this information sheet and for considering taking part in this research study
