# Supplementary material for: Phase II multicentre, double-blind, randomised trial of ustekinumab in adolescents with new-onset type 1 diabetes (USTEK1D): trial protocol
Source: BMJ Open. 2021 Oct 18;11(10):e049595. doi: 10.1136/bmjopen-2021-049595 (PMC8524290; doi:10.1136/bmjopen-2021-049595)
Supplement: Supplementary data [file bmjopen-2021-049595supp004.pdf]

[Insert local headers]

USTEKID

NHS National Institute for Health Research

CARDIFF UNIVERSITY PRIFYSGOL CAERDYDD

Swansea University Prifysgol Abertawe
Swansea Trials Unit Uned Dreialon Abertawe

A research study to see if the medicine Ustekinumab can make diabetes easier to manage

<u>CONTACT DETAILS FOR STUDY TEAM</u>:

NURSE:

DOCTOR:

EMERGENCIES:

FOR PARENTS OF YOUNG PEOPLE AGED 16-18 YEARS OLD

We would like to invite your child to help us with our research study. It is important for you to understand why the research is being done and what it will involve as we also need some information from you. Please take time to read the following information carefully and discuss it with friends and relatives if you wish. If anything is unclear or you need to know more, please ask us.

Please look at our video explaining the trial at www.type1diabetesresearch.org.uk/current-trials. The blue box below and the video contains the key points about the study. If you would like to know more, please read the rest of this leaflet.

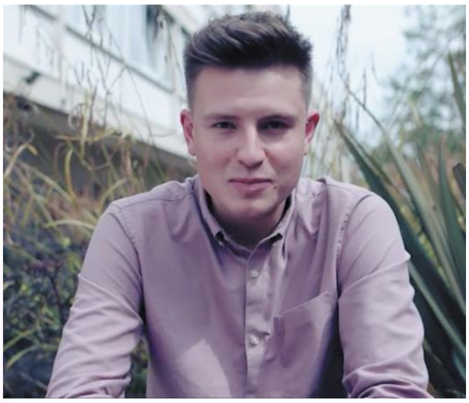

<u>KEY POINTS ABOUT THE STUDY</u>:

USTEKID

- We want to see if the study medicine, **Ustekinumab**, can make Type 1 diabetes (the type your child has) easier to manage. The medicine works by "protecting" some of the cells in the pancreas that still produce insulin from attack by the immune system.
- The study will involve your child having an injection every 1-3 months with either the study medicine, Ustekinumab or a placebo (a "dummy medicine"). These injections are given under the skin just like insulin injections and will be done by the study doctor or nurse. Neither you nor the research team will know if your child is receiving the study medicine or the placebo.
- The medicine is already being used to treat other illnesses quite safely.
- Your child will come to your local hospital or research centre for 10 study visits over a 15 month period, but where possible this will be on the same day as their routine hospital visits. The first two will check if you are eligible to take part. Three of the visits can be sometimes done by a research nurse at home. You will be asked to come with them for three of these study visits to complete a questionnaire.
- We will ask your child to provide extra blood and urine samples for the study.
- We will provide your child with a flash blood glucose monitor (Freestyle Libre) to wear for 2 weeks before each visit. They can keep the monitor for use at home for the whole time of the study.
- Your child will be offered a small gift voucher for each visit.
- You can stop taking part in the study at any time and you do not have to give a reason why. It will not affect your child taking part in the study.

Gregory JW, *et al. BMJ Open* 2021; 11:e049595. doi: 10.1136/bmjopen-2021-049595

WHY IS THIS STUDY BEING DONE?

This study is being done to see if a medicine called **Ustekinumab** can help to "protect" the cells in the body that produce insulin in young people recently diagnosed with Type 1 Diabetes . It is caused by the body's own immune system damaging the cells in the pancreas that make insulin. Our aim is to develop a treatment that can slow this process by targeting the immune cells causing the damage.

At the time of diagnosis, most children have 10-20% of their insulin-producing cells still working. It usually takes between 1 and 5 years before they stop working completely. Sometimes these last few working cells can make enough insulin to make blood sugar levels stable and easier to control—this is called the "Honeymoon period". This period is only temporary and doesn't last. Ustekinumab, the study medicine, may make this period last longer by reducing the damaging effects of the immune system on the remaining insulin-producing cells in the pancreas.

Ustekinumab is currently given to adults and teenagers with particular skin and bowel problems and it is known to be safe and effective at treating those conditions.

WHY HAS MY CHILD BEEN ASKED TO TAKE PART?

Your child has been chosen because they are aged 12 - 18 years old and has recently been diagnosed with Type 1 diabetes. You are being told about the study because if they agree to take part, we need a parent/carer to complete three questionnaires for us.

DO I HAVE TO TAKE PART?

No. It is completely up to you whether or not you take part. It will not affect your child taking part in the study. Both you and your child are free to change your minds at any time.

If your child is interested in this study then they can let us know by calling one of the people listed at the end of their information sheet and a member of the research team will contact them and you to explain more about the study and answer any questions you may both have.

WHAT HAPPENS ONCE MY CHILD AGREEDS TO TAKE PART?

Before we can start the treatment, we need to check that your child is eligible to take part in the study - this is called "screening". Your child will be asked to come to your local hospital or research centre to talk about the study. This is where you will both be able to ask questions.

You will be invited to attend the screening visits and asked to consider whether you would be happy to consent to completing three questionnaires, one at the second screening visit, one 28 weeks after their first study treatment and one 52 weeks after their first study treatment. The person consenting should be the person who will complete all three study questionnaires. If you are happy to do so, we will ask you to sign a consent form and begin completing the first questionnaire at the second screening visit.

If you would like more details on your child's role in the study, please ask to see their information sheet which details the visits and tests being done.

WHAT WILL I BE ASKED TO DO?

As well as attending with your child to your local hospital or research centre for study visits that require you to complete questionnaires, there are a few things we will ask your child to do during the study which may involve your help:

- Your child will be asked to complete a diary between study visits to record:
    a) How much insulin they take during the study (for the two weeks before every study visit)
    b) If they feel unwell or have to take any medicines during the study.
    c) If they have any hypoglycaemic (low blood glucose level) episodes that need treating.
- We will ask them to test their blood glucose levels at home for at least 2 weeks before each visit using our FREE Abbott Freestyle Libre glucose monitoring system so they don't need to do extra finger prick tests for the trial. They will need to wear a sensor on their arm. We will show you both how it works. They are free to use the monitor at home for the rest of the time of study if they want to. We will give them enough sensors to allow this for a year.

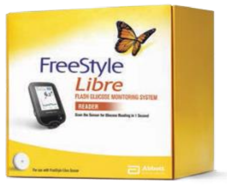

Gregory JW, et al. BMJ Open 2021; 11:e049595. doi: 10.1136/bmjopen-2021-049595

- We will ask them to give a blood spot sample which they can do at home. They will need to do this once a week for 28 weekly then every month for the next 6 months. We will show you both how to do this and will provide envelopes and pay for the postage.
- You will be asked to make sure that your child does not have certain vaccinations before, during and immediately after the study. If they need a vaccination, for example if they are travelling abroad, you must tell the study doctor or nurse immediately.

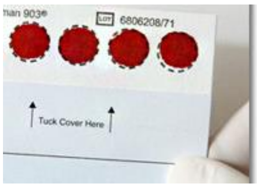

WILL THE STUDY HELP MY CHILD?

Your child has been allocated to the group that receives the study medicine Ustekinumab, it is possible that it will help their pancreas make insulin for longer. However we cannot say this for certain until we have completed this study. During the study your child's diabetes will be very closely monitored. This will include regular check-ups with your local diabetes team including routine blood testing. You and your child will have more time with the research team to discuss their diabetes and ask questions than at a normal clinic appointment.

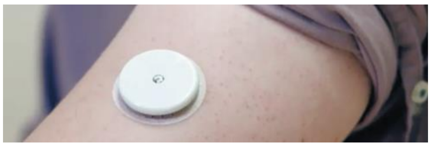

Your child will be provided with a FREE Abbott Freestyle Libre blood glucose monitoring system. They can use this to check their blood glucose levels while they are in the study.

Injections with the study medicine or the placebo will be done at weeks 0, 4, 12, 20, 28, 36 and 44. After this, no more treatments will be given to your child.

WHAT HAPPENS WHEN THE STUDY STOPS?

We will collect all the information together and we will decide if the study medicine can help people with Type 1 Diabetes make their own insulin for longer. If it does then we will carry out a bigger version of this study.

WHAT IF NEW INFORMATION COMES ALONG?

Sometimes during research, we get new information about the treatment being studied. If this happens, we will tell you about it and discuss whether you want to continue in the study.

WHAT IF I DO NOT WANT TO TAKE PART ANYMORE?

Just let the study doctor or research nurse know about your decision and we will make sure that you are not asked to complete further questionnaires. If you withdraw, this will not affect your child taking part in the study.

WHAT IF THERE IS A PROBLEM OR SOMETHING GOES WRONG?

If there are any problems, please contact the study team using the details at the end of this form.

If you feel overwhelmed by your child's recent diagnosis, you can call the local [title] on [tel number / email] and they can talk through your concerns with you.

If you are unhappy about the conduct of the study and wish to complain, you can do this through: (name and contact details of appropriate organisations – site specific).

WHAT ARE THE POSSIBLE SIDE EFFECTS FOR ME IN PARTICIPATING IN THE STUDY?

You are only being asked to complete three short questionnaires. We do not anticipate any risk being involved.

The risks for your child for taking part in the study are fully explained in their information sheet.

WILL I RECEIVE ANY PAYMENT FOR TAKING PART?

You will be able to claim back your travel expenses getting your child to the local hospital or research centre.

WHAT INFORMATION WILL YOU COLLECT AND HOW WILL IT BE KEPT PRIVATE?

We will ask for your name and contact details as well as your child's name so that the research nurse can keep in touch and manage your visits. We will also collect the information you record on your questionnaire and enter it into a database.

The people in our research team at the local hospital or research centre will know that your child is taking part. The doctors looking after your child when you come to hospital will also know that they are in the study. Their medical

notes may be looked at by staff from Swansea and Cardiff Universities who will be checking that the study is being done correctly. If your child has agreed, we will also tell your family doctor (GP) that they are in the study.

You and your child will be given a study number to replace their name so any study samples and information related to you both will be anonymised.

All information collected about you and your child during the study will be kept by the research nurse in a locked cabinet and entered onto a secure database. Only people with the password can open up the database.

Cardiff University is the sponsor for this study based in the United Kingdom. Cardiff University will be using information from you and your medical records in order to undertake this study and will act as the data controller for this study. This means that we are responsible for looking after your information and using it properly. Cardiff University will keep identifiable information about you for 25 years after the study has finished.

Your rights to access, change or move your information are limited, as we need to manage your information in specific ways in order for the research to be reliable and accurate. If you withdraw from the study, we will keep the information about you that we have already obtained. To safeguard your rights, we will use the minimum personally-identifiable information possible. You can find out more about how we use your information by contacting inforequest@cardiff.ac.uk.

WHAT WILL HAPPEN TO THE RESULTS OF THIS STUDY?

The full results of this study will not be known until the last patient has completed their tests, which may take more than 5 years. The research results will be reported in scientific publications and meetings but you will not be identified by name at all. If you are interested in receiving a summary of the research results, we can arrange this.

WHO IS ORGANISING AND FUNDING THE STUDY?

The study is being organised by researchers at Cardiff University and Swansea University. It is being funded by a grant from the National Institute for Health and Research (NIHR).

WHERE IS THE STUDY BEING DONE?

The study is being done at hospitals and research centres across England, Wales and Scotland.

WHO HAS CHECKED THIS STUDY?

Before any research goes ahead it has to be checked by a Research Ethics Committee. This is a group of people who make sure that the research is OK to do and to make sure that the patient will be safe. This study has been looked at by Wales REC 3. As this study is looking at a medicine, it has also been approved by the government's Medicine and Healthcare products Regulatory Authority (MHRA) who check that the researchers carry out the study safely. It has also been checked by national and local NHS organisations to make sure that the study can be done using their site and staff.

WHAT SHOULD I DO NOW?

If you are interested in taking part, or have any questions please contact one of the following people:

**Name:**	**Name:**	**Name:** (only if needed)
**Role:** Principal Investigator	**Role:** Research nurse	**Role:**
**Tel. No:**	**Tel. No:**	**Tel. No:**
**Email:**	**Email:**	**Email:**

Alternatively, you may want to speak to someone at the USTEKID Trial Office who are managing the study, based at Swansea University. The Trial Manager's details are below:

Name: Dr Kym Thorne
Tel. No: 01792 606372 (direct) or 01792 606545 for Swansea Trials Unit
Email: ustekid@swansea.ac.uk
Address: Floor 2, Institute of Life Sciences 2, Swansea University Medical School, Singleton Park, Swansea SA2 8PP

Thank you for taking the time to read this information sheet and for considering taking part in this research study
