# Supplementary material for: Phase II multicentre, double-blind, randomised trial of ustekinumab in adolescents with new-onset type 1 diabetes (USTEK1D): trial protocol
Source: BMJ Open. 2021 Oct 18;11(10):e049595. doi: 10.1136/bmjopen-2021-049595 (PMC8524290; doi:10.1136/bmjopen-2021-049595)
Supplement: Supplementary data [file bmjopen-2021-049595supp005.pdf]

Insert logos

USTEKID

Phase II multi-centre, double-blind, randomised trial of Ustekinumab in adolescents with new-onset type 1 diabetes (USTEKID)

**Chief Investigator:**	Prof Colin Dayan	**Principal Investigator:**	
**Site ID:**		**Participant study number:**	
**Participant name (in capitals):**			

ASSENT FORM FOR YOUNG ADOLESCENTS (AGED 12-15y)

(to be completed by the child with help from and their parent/carer)

Circle either Yes or No to the following

Have you read the information sheet about the study (or has someone read it to you)?	YES	NO
Has somebody explained this study to you?	YES	NO
Do you understand what this study is about?	YES	NO
Have you asked all the questions you want?	YES	NO
Has someone answered your questions in a way that you understand?	YES	NO
Do you understand that it is OK to stop taking part at any time?	YES	NO
Are you happy to give samples of blood and wee (urine)?	YES	NO
Are you happy to take part?	YES	NO

If you do not want to take part, do not sign your name.
If you are not happy with what you have circled YES to, do not sign your name.

If you do want to take part, you should write your name clearly below.

Your name ____

Please write the date ______________________

The person who explained the study to you also needs to sign this form:

______________________ ______________________ ______________________
Researcher Name Signature Date

USTEKID IAF 12-15y v3.0 10Jun2019 IRAS ID: 230113 EudraCT Number: 2018-000015-24
