# Supplementary material for: Phase II multicentre, double-blind, randomised trial of ustekinumab in adolescents with new-onset type 1 diabetes (USTEK1D): trial protocol
Source: BMJ Open. 2021 Oct 18;11(10):e049595. doi: 10.1136/bmjopen-2021-049595 (PMC8524290; doi:10.1136/bmjopen-2021-049595)
Supplement: Supplementary data [file bmjopen-2021-049595supp006.pdf]

USTEKID

Insert logos

# Phase II multi-centre, double-blind, randomised trial of Ustekinumab in adolescents with new-onset type 1 diabetes (USTEKID)

| | | | |
|---|---|---|---|
| **Chief Investigator:** | Prof Colin Dayan | **Principal Investigator:** | |
| **Site ID:** | | **Participant study number:** | |

## CONSENT FORM FOR PARENTS/CARERS OF YOUNG ADOLESCENTS (AGED 12-15y)

**Please initial boxes**

1. I confirm that I have read and understand the 12-15y Parent Information Sheet dated (………………) (version......) for the above study. I have had the opportunity to consider the information, ask questions and have had these answered satisfactorily.
2. I understand that my child's participation is voluntary and that we are free to withdraw at any time without giving any reason, without his/her medical care or legal rights being affected.
3. I agree to my child attending screening and study visits and to being randomised to receive either the study medicine or the placebo.
4. I agree that my child can provide urine and blood samples for the study.
5. I agree that my child can do dried blood spot testing at home for the study.
6. I agree that my child can wear the FreeStyle Libre glucose monitor at least two weeks prior to each study visit.
7. I agree that my child can complete diaries and questionnaires for the study.
8. I agree to complete study questionnaires myself.
9. I understand that the information collected from me and my child in study questionnaires will be viewed by the research team and will be stored securely.
10. I agree that if my child is involved in actions which may lead to pregnancy, they will take adequate contraception (hormonal based contraception, barrier contraception, abstinence) until 4 months following the date of final treatment.
11. I give permission for relevant sections of my child's medical notes and data collected during the study to be looked at by responsible individuals from the USTEKID research team, from regulatory authorities or from Cardiff University (as Sponsor), where it is relevant to my child taking part in this research.
12. I understand that the information collected about my child may be used to support other research in the future, and may be shared anonymously with other researchers.
13. I understand that the information held and maintained in local hospital records and other central UK NHS bodies may be used to help contact myself or my child, or to provide information about my child's health status during the study follow up.
14. I understand and agree that my child's anonymised blood samples may be used for analysis by the study or other relevant studies if they obtain the relevant permissions.
15. I agree to my child's anonymised blood samples being stored in a Human Tissue Authority (HTA) repository for future ethically approved studies.
16. I agree that my child's anonymised blood samples may be transported within and outside the European Union for analysis in specialist laboratories.
17. I agree to my child's GP being notified of his/her involvement in the study, including any necessary exchange of information about my child between my GP and the research team.
18. I agree to my child taking part in the above study.

**For the participant's parent/carer**

NAME_________________________SIGNATURE______________________ DATE_______________

**For the person taking consent**

NAME_________________________SIGNATURE______________________ DATE_______________

USTEKID ICF parent 12-15y v3.0 10Jun2019 IRAS ID: 230113 EudraCT Number: 2018-000015-24
