# Supplementary material for: Phase II multicentre, double-blind, randomised trial of ustekinumab in adolescents with new-onset type 1 diabetes (USTEK1D): trial protocol
Source: BMJ Open. 2021 Oct 18;11(10):e049595. doi: 10.1136/bmjopen-2021-049595 (PMC8524290; doi:10.1136/bmjopen-2021-049595)
Supplement: Supplementary data [file bmjopen-2021-049595supp007.pdf]

USTEKID

Insert logos

Phase II multi-centre, double-blind, randomised trial of Ustekinumab in adolescents with new-onset type 1 diabetes (USTEKID)

**Chief Investigator:**	Prof Colin Dayan	**Principal Investigator:**	
**Site ID:**		**Participant study number:**	

CONSENT FORM FOR ADOLESCENTS (AGED 16-18y)

Please initial boxes

1. I confirm that I have read and understand the 16-18y Patient Information Sheet dated (………………) (version......) for the above study. I have had the opportunity to consider the information, ask questions and have had these answered satisfactorily.
2. I understand that my participation is voluntary and that I am free to withdraw at any time without giving any reason, without my medical care or legal rights being affected.
3. I agree to attend screening and study visits and to being randomised to receive either the study medicine or the placebo.
4. I agree to provide urine and blood samples for the study.
5. I agree to do dried blood spot testing at home for the study.
6. I agree to wear the FreeStyle Libre glucose monitor at least two weeks prior to each study visit.
7. I agree to complete diaries and questionnaires for the study.
8. I agree that if I am involved in actions that may lead to pregnancy, I will take adequate contraception (hormonal based contraception, barrier contraception, abstinence) until 4 months following the date of final treatment.
9. I give permission for relevant sections of my medical notes and data collected during the study to be looked at by responsible individuals from the USTEKID research team, from regulatory authorities or from Cardiff University (as Sponsor), where it is relevant to my taking part in this research.
10. I understand that the information collected about me may be used to support other research in the future, and may be shared anonymously with other researchers.
11. I understand that the information held and maintained in local hospital records and other central UK NHS bodies may be used to help contact me or provide information about my health status during the study follow up.
12. I understand and agree that my anonymised blood samples may be used for analysis by the study or other relevant studies if they obtain the relevant permissions.
13. I agree to my anonymised blood samples being stored in a Human Tissue Authority (HTA) repository for future ethically approved studies.
14. I agree that my anonymised blood samples may be transported within and outside the European Union for analysis in specialist laboratories.
15. I agree to my GP being notified of my involvement in the study, including any necessary exchange of information about me between my GP and the research team.
16. I agree to take part in the above study.

For the participant

NAME___________________________________SIGNATURE________________________ DATE__________________

For the person taking consent

NAME___________________________________SIGNATURE________________________ DATE__________________

USTEKID ICF 16-18y v3.0 10Jun2019 IRAS ID: 230113 EudraCT Number: 2018-000015-24
