# Supplementary material for: Phase II multicentre, double-blind, randomised trial of ustekinumab in adolescents with new-onset type 1 diabetes (USTEK1D): trial protocol
Source: BMJ Open. 2021 Oct 18;11(10):e049595. doi: 10.1136/bmjopen-2021-049595 (PMC8524290; doi:10.1136/bmjopen-2021-049595)
Supplement: Supplementary data [file bmjopen-2021-049595supp008.pdf]

Phase II multi-centre, double-blind, randomised trial of Ustekinumab in adolescents with new-onset type 1 diabetes (USTEKID)

**Chief Investigator:**	Prof Colin Dayan	**Principal Investigator:**	Prof John Gregory
**Site ID:**		**Participant study number:**	
**Participant name (in capitals):**			

CONSENT FORM FOR PARENT/CARER OF ADOLESCENTS (AGED 16-18y)

Please initial the boxes

1. I confirm that I have read and understand the 16-18y Parent Information Sheet dated 2nd July 2019, version 3 for the above study. I have had the opportunity to consider the information, ask questions and have had these answered satisfactorily.
2. I understand that my participation is voluntary and that I am free to withdraw at any time without giving any reason, without my child's medical care or legal rights being affected.
3. I agree to complete study questionnaires.
4. I understand that the information collected from me in study questionnaires will be viewed by the research team and will be stored securely.
5. I agree to take part in the above study.

For the participant's parent/carer

NAME____________________________SIGNATURE________________________ DATE________________

For the person taking consent

NAME____________________________SIGNATURE________________________ DATE________________

USTEKID ICF parent 16-18y v3.0 10Jun2019 IRAS ID: 230113 EudraCT Number: 2018-000015-24
