# Supplementary material for: Phase II multicentre, double-blind, randomised trial of ustekinumab in adolescents with new-onset type 1 diabetes (USTEK1D): trial protocol
Source: BMJ Open. 2021 Oct 18;11(10):e049595. doi: 10.1136/bmjopen-2021-049595 (PMC8524290; doi:10.1136/bmjopen-2021-049595)
Supplement: Author's manuscript [file bmjopen-2021-049595.draft_revisions.pdf]

BMJ Open

BMJ Open is committed to open peer review. As part of this commitment we make the peer review history of every article we publish publicly available.

When an article is published we post the peer reviewers' comments and the authors' responses online. We also post the versions of the paper that were used during peer review. These are the versions that the peer review comments apply to.

The versions of the paper that follow are the versions that were submitted during the peer review process. They are not the versions of record or the final published versions. They should not be cited or distributed as the published version of this manuscript.

BMJ Open is an open access journal and the full, final, typeset and author-corrected version of record of the manuscript is available on our site with no access controls, subscription charges or pay-per-view fees (http://bmjopen.bmj.com).

If you have any questions on BMJ Open's open peer review process please email info.bmjopen@bmj.com

Phase II multi-centre, double-blind, randomised trial of ustekinumab in adolescents with new-onset Type 1 Diabetes (USTEK1D): Trial Protocol

Journal:	*BMJ Open*
Manuscript ID	bmjopen-2021-049595
Article Type:	Protocol
Date Submitted by the Author:	25-Feb-2021

Complete List of Authors:	Gregory, John; Cardiff University, Division of Population Medicine<br>Carter, Kymberley; Swansea University, Swansea Trials Unit<br>Cheung, Ivy; Swansea University, Diabetes Research Group<br>Holland, Gail; Swansea University, Swansea Trials Unit;<br>Bowen-Morris, Jane; Cardiff University, Diabetes Research Group<br>Luzio, Stephen; Swansea University, Diabetes Research Group<br>Dunseath, Gareth; Swansea University, Diabetes Research Group<br>Tree, Timothy<br>Yang, Jennie; King's College London, Department of Immunobiology<br>Marwaha, Ashish; University of Toronto, Department of Molecular Genetics<br>Ali, Mohammad; Cardiff University, Diabetes Research Group<br>Bashir, Nadim; Swansea University, Trials Unit<br>Hutchings, Hayley; Swansea University, Swansea Trials Unit<br>Fegan, Greg; Swansea University, Swansea Trials Unit<br>Stenson, Rachel; Cardiff University, Diabetes Research Group<br>Hiles, Stephen; Swansea University, Swansea Trials Unit<br>Marques-Jones, Susie; Patient and Public Representative<br>Brown, Amy; Patient and Public Representative<br>Tatovic, Danijela; Cardiff University, Diabetes Research Group<br>Dayan, Colin; Cardiff University School of Medicine

Keywords:	Paediatric endocrinology < DIABETES & ENDOCRINOLOGY, STATISTICS & RESEARCH METHODS, Diabetes & endocrinology < INTERNAL MEDICINE

SCHOLARONE™
Manuscripts

I, the Submitting Author has the right to grant and does grant on behalf of all authors of the Work (as defined in the below author licence), an exclusive licence and/or a non-exclusive licence for contributions from authors who are: i) UK Crown employees; ii) where BMJ has agreed a CC-BY licence shall apply, and/or iii) in accordance with the terms applicable for US Federal Government officers or employees acting as part of their official duties; on a worldwide, perpetual, irrevocable, royalty-free basis to BMJ Publishing Group Ltd ("BMJ") its licensees and where the relevant Journal is co-owned by BMJ to the co-owners of the Journal, to publish the Work in this journal and any other BMJ products and to exploit all rights, as set out in our licence.

The Submitting Author accepts and understands that any supply made under these terms is made by BMJ to the Submitting Author unless you are acting as an employee on behalf of your employer or a postgraduate student of an affiliated institution which is paying any applicable article publishing charge ("APC") for Open Access articles. Where the Submitting Author wishes to make the Work available on an Open Access basis (and intends to pay the relevant APC), the terms of reuse of such Open Access shall be governed by a Creative Commons licence – details of these licences and which Creative Commons licence will apply to this Work are set out in our licence referred to above.

Other than as permitted in any relevant BMJ Author's Self Archiving Policies, I confirm this Work has not been accepted for publication elsewhere, is not being considered for publication elsewhere and does not duplicate material already published. I confirm all authors consent to publication of this Work and authorise the granting of this licence.

For peer review only

Phase II multi-centre, double-blind, randomised trial of ustekinumab in adolescents with new-onset Type 1 Diabetes (USTEK1D): Trial Protocol

Corresponding author: Dr Kym Thorne, Swansea Trials Unit, Floor 2, ILS2, Swansea University Medical School, Singleton Park, Swansea, UK, SA2 8PP k.thorne@swansea.ac.uk

John W. Gregory [1]

Kym Thorne [2]

Ivy Cheung [3]

Gail Holland [2]

Jane Bowen-Morris [4]

Steve Luzio [3]

Gareth Dunseath [3]

Tim Tree [5]

Jennie Yang [5]

Ashish Marwaha [6]

Mohammad Alhadj Ali [4]

Nadim M. Bashir [2]

Hayley A. Hutchings [2]

Greg W. Fegan [2]

Rachel Stenson [4]

Stephen Hiles [2]

Susie Marques Jones [7]

Amy Brown [7]

Danijela Tatovic [4]

Colin M. Dayan [4]

1 Division of Population Medicine, Cardiff University School of Medicine, Cardiff, UK

2 Swansea Trials Unit, Swansea University Medical School, Singleton Park, Swansea, UK

3 Diabetes Research Unit Cymru, Swansea University Medical School, Singleton Park, Swansea, UK

4 Diabetes Research Group, Cardiff University School of Medicine, Cardiff, UK

5 Department of Immunobiology, Faculty of Life Sciences and Medicine, King's College London, London, UK

6 Department of Molecular Genetics, University of Toronto, Toronto, Ontario, Canada

7 Patient / Parent representatives with no affiliation to an organisation

Word count, excluding title page, abstract, references, figures and tables: 4076

For peer review only - http://bmjopen.bmj.com/site/about/guidelines.xhtml

For peer review only

ABSTRACT

Introduction

Most individuals newly diagnosed with Type 1 Diabetes (T1D) have 10-20% of beta-cell function remaining at the time of diagnosis. Preservation of residual beta-cell function at diagnosis, may improve glycaemic control and reduce longer term complications.

Immunotherapy has the potential to preserve endogenous beta-cell function and thereby improve metabolic control even in poorly compliant individuals. We propose to test ustekinumab (STELARA®), a targeted and well-tolerated therapy that may halt T-cell and cytokine-mediated destruction of beta-cells in the pancreas at the time of diagnosis.

Methods and analysis

This is a double-blind Phase II study to assess the safety and efficacy of ustekinumab in 72 children and adolescents aged 12-18 with new-onset T1D.

Participants should have evidence of residual functioning beta-cells (serum C-peptide level >0.2nmol/L in the Mixed Meal Tolerance Test (MMTT) and be positive for at least one islet autoantibody (GAD, IA-2, ZnT8) to be eligible.

Participants will be given ustekinumab/placebo subcutaneously at weeks 0, 4 and 12, 20, 28, 36 and 44 in a dose depending on the body weight and will be followed for 12 months after dose 1.

MMTTs will be used to measure the efficacy of ustekinumab for preserving C-peptide area under the curve at week 52 compared with placebo. Secondary objectives include further investigations into the efficacy and safety of ustekinumab, patient and parent questionnaires, alternative methods for measuring insulin production and exploratory mechanistic work.

Ethics and dissemination.

This trial received research ethics approval from Wales REC 3 in September 2018 and began recruiting in December 2018.

The results will be disseminated using highly accessed, peer-reviewed medical journals and presented at conferences.

Trial ISRCTN ID: 14274380.

Protocol v4.0 dated 4th May 2020.

STRENGTHS AND LIMITATIONS OF THIS STUDY

- This trial is being undertaken in 16 sites across the UK (England = 12, Scotland = 2 and Wales = 2).
- The trial will provide evidence towards the efficacy and safety of treating new onset Type 1 diabetes in 12-18 year olds with ustekinumab (Stelara ®).
- We have included an extensive range of secondary and exploratory outcomes to investigate the efficacy and safety of ustekinumab, and to expand existing methodological designs in this field.
- Recruitment period is two years due to low numbers of diagnoses at sites and number subsequently agreeing to consent.

For peer review only

For peer review only - http://bmjopen.bmj.com/site/about/guidelines.xhtml

INTRODUCTION

Nearly 100 years after the discovery of insulin, over 70% of patients with Type 1 diabetes (T1D) continue to have unsatisfactory glycaemic control putting them at risk of long-term complications.(1) Tragically, death rates amongst adolescents have not improved in the last few decades (1968-2009).(2, 3) Despite major advances in closed loop insulin pump therapy, much of the morbidity arises from young people failing to engage with complex therapies.

Most individuals have 10-20% of beta-cell function remaining at the time of diagnosis of T1D.(4) Preservation of even 5% of beta-cell function has been shown to: lower blood glucose levels (as measured by HbA1c tests) by 1%; permit over 50% of people to reach target glycaemic levels; reduce hypoglycaemic risk by >50%; and reduce long-term complications by 50%.(5, 6) Immunotherapy has the potential to preserve endogenous beta-cell function and thereby improve metabolic control even in poorly compliant individuals.(7-9)

Novel low-risk targeted biologic therapies are widely used in other autoimmune diseases such as rheumatoid arthritis, psoriasis and inflammatory bowel disease, but no treatment was licensed for use in T1D. Ustekinumab is licenced in the UK for the treatment of psoriasis in children and adults, psoriatic arthritis in adults and Crohn's disease in adults.

Extensive evidence exists to implicate two major autoimmune cytokine pathways, IL-12/IFN-γ and IL-23/IL-17, in beta cell destruction. Ustekinumab (STELARA®), binds and inhibits the p40 molecular subunits of both IL-12 and IL-23 thus blocking their action in inducing pathogenic CD4 Th1 and Th17 T cell subsets.(10) Our overarching hypothesis is that interrupting the IL-17 and IFN-γ axes in individuals with recent-onset T1D will halt or slow the autoimmune destruction of beta cells sufficiently to permit beta cell preservation and maintain residual physiological insulin secretion. Given the therapeutic success of biologics that target immune molecules in other autoimmune and inflammatory diseases, and the evidence that IL-17 and IFN-γ producing cells are pathogenic to beta cells, we propose that ustekinumab may be beneficial for the treatment of T1D.

This paper presents the protocol for a double blind, multicentre, randomised phase II trial to evaluate the effect of ustekinumab in patients aged 12-18 years with new onset T1D.

METHODS

Overview

This is a multicentre, double blind, randomised, controlled trial comparing ustekinumab with placebo (2:1 ratio). Doses of ustekinumab will be 2mg/Kg body weight if the child is ≤40Kg and 90mg if >40Kg. Doses will be administered at week 0, 4, 12, 20, 28, 36 and 44 and with follow up at week 52 (see Figure 1). The study will be carried out in 12 to 18-year-olds within 100 days of diagnosis of T1D in 16 sites across mainland UK.

The primary objective is to determine the efficacy of ustekinumab for preserving mixed meal tolerance test (MMTT) stimulated 2-hour plasma C-peptide area under the curve (AUC) at week 52 as compared to placebo. This follows the rationale published by Greenbaum. (11)

Objectives

Table 1 details the trial objectives, outcome measures and time points for data analysis.

Objectives	Outcome Measures
**Primary Objective**	

For peer review only - http://bmjopen.bmj.com/site/about/guidelines.xhtml

Objectives	Outcome Measures
To determine the efficacy of ustekinumab (dose: 2mg/kg (≤40kg); 90mg (>40kg)) for preserving MMTT stimulated 2-hour C-peptide area under the curve (AUC) at week 52 as compared to placebo in children and adolescents with new-onset T1D.	MMTT C-peptide AUC values at week 52 at week 52
**Secondary Objectives**	
1. To determine the efficacy of the ustekinumab dosing to elicit response to treatment.	Number of responders (defined as participant who has HbA1c ≤ 48mmol/mol and mean daily insulin use <0.5 IU/kg/day) measured over 7 consecutive days during the 2 weeks preceding the visit in treatment and placebo group at week 52
2. To investigate additional efficacy (metabolic) endpoints including MMTT C-peptide AUC at week 28, HbA1c and insulin use measurements at week 52.	MMTT C-peptide AUC values at Week 28
	HbA1c at weeks 0, 12, 28 and 52
	Exogenous insulin requirement as reflected in mean daily insulin usage over 7 consecutive days (IU units/kg body weight/day) as recorded in diaries prior to study visits at weeks 12, 28 and 52
	Insulin dose adjusted HbA1c (IDAAC) at week 52
3. To compare alternative metabolic endpoint assays to MMTT: including glycaemic variability in glucose monitoring systems –Freestyle Libre) and hypoglycaemia rates.	Glycaemic variability parameters downloaded from glucose monitoring at each study visit, e.g.<br>• Blood glucose level at 1,2,3 hours before and after each meal<br>• Number of episodes and length of time within the following glucose level: below 4.0 mmol/L, >10 mmol/L and >15 mmol/L<br>• % Time hypoglycaemic (<3.0 mmol/ and <4.0 mmol)
	Clinical hypoglycaemic events determined by patient diary reports and AE reports at week 52
4. To determine the safety of ustekinumab dose in adolescents with new-onset T1D.	Frequency and severity of all adverse events at week 52
5. To compare between treatment arms and across the course of treatment the age appropriate PROMs scores completed by participants and parents/carers.	HYPOFEAR, DTSQ, and PedsQL questionnaires completed by participants and their parent/carer at weeks -2, 28 and 52
**Exploratory objectives**	
1. To investigate alternative ways of measuring insulin production other than MMTT C-peptide.	Proinsulin, Glucagon, somatostatin levels, Dried blood spot (DBS) C-peptide at weeks 28 and 52
	DBS C-peptide vs MMTT C-peptide at weeks -2, 28 and 52

For peer review only - http://bmjopen.bmj.com/site/about/guidelines.xhtml

Objectives	Outcome Measures
2. To investigate changes in relevant immune mechanistic parameters include flow cytometry immune phenotyping of all IL-17 and IFN-gamma secreting T cell subsets, fluorospot analysis for IL-17 and IFN-gamma secretion in response to antigens for CD4+ T cells.	Changes at weeks 12, 28 and 52 in:<br>• immune phenotype of all IL-17, IFN-g secreting immune subsets<br>• T cell responses to antigens or peptides derived from islet antigens (including proinsulin, GAD and IA-2) measured by cytokine FLUOROSPOT (IFN-g and IL-17)<br>• T cell responses to antigens or peptides derived from islet antigens (including proinsulin, GAD, IA-2) measured by the level of IFNg, IL-17, IL-12 and IL-23 production in supernatants (Luminex)<br>• additional immunological biomarkers (e.g. flow cytometry profiles, T cell responsiveness measured by activation profiles, T reg assays, autoantibodies)
3. To investigate ustekinumab pharmacokinetics (PK) and compliance with therapy	Ustekinumab drug levels in serum at weeks 4, 12, 28 and 52
4. To explore association of C-peptide changes with age-appropriate PROMs.	C-peptide AUC and HYPOFEAR, DTSQ, and PedsQL questionnaires at weeks -2, 28 and 52
5. To compare participant and parent/carer proxy completed PROMs.	HYPOFEAR, DTSQ, and PedsQL questionnaires completed by participants and their parent/carer at weeks -2, 28 and 52
6. To investigate the longer term effect of ustekinumab on glycaemic control.	• Severe hypoglycaemic events<br>• Insulin use<br>• HbA1c<br>• C-peptide using DBS samples<br>• CGM data<br>At weeks 78 and 104

Table 1 – Objectives and outcome measures

Table key: -2 refers to the second screening visit ~ 2 weeks prior to dose 1.

For peer review only

Consent

Potential participants identified from health records, clinical contacts, patient registries and self-referrals through the T1DUK consortium and ADDRESS-2 website will be asked to view our short recruitment video (https://www.youtube.com/watch?v=8kuCefuBSW4) followed by a more detailed information sheet relevant to their age (see Appendices 1-4).

Written informed consent will be obtained for all participants at the first screening visit (see Appendices 5-8). For participants under 16, written assent will be obtained in addition to written consent from a parent/carer. Reconsent will be requested when participants turn 16.

Eligibility criteria

Consented participants will have eligibility checks (see Table 2), including auto-antibody screening and a MMTT. Tuberculosis must be ruled out using a chest x-ray and either a Mantoux test or a blood-based TB test. All blood and urine tests must be within clinically normal parameters.

For peer review only - http://bmjopen.bmj.com/site/about/guidelines.xhtml

The first dose of Investigational Medical Prduct (IMP) must be given within 100 days from clinical diagnosis. The screening MMTT must be within 37 days of the first dose of IMP.

INCLUSION CRITERIA	EXCLUSION CRITERIA
Clinical diagnosis of immune-mediated Type 1 diabetes mellitus as defined by the American Diabetes Association (ADA) (12, 13).	Breastfeeding, pregnancy or unwillingness to comply with contraceptive advice and regular pregnancy testing throughout the trial.
Commenced on insulin within 1 month of **clinical** diagnosis (defined as confirmed raised blood sugar (ADA criteria), not symptoms alone).	Prior exposure to ustekinumab within 3 months of the first dose of IMP.
An interval of ≤100 days between the **confirmed** diagnosis (defined as date of first insulin dose) and the first planned dose of the IMP.	Use of more than 10mg prednisolone daily (or equivalent) for >5 days within 3 months of the first dose of IMP.
Written and witnessed informed consent/assent to participate.	Prior exposure to any anti-lymphocyte monoclonal antibody, such as anti-CD20, anti-thymocyte globulin (ATG), Rituximab (Rituxan®), or Alemtuzumab (Campath®).
Male or female, aged 12-18 years inclusive at the time of randomisation.	Use of immunosuppressive or immunomodulatory therapies, including systemic steroids within 30 days prior to receiving the first dose and/or intent on using any monoclonal antibody therapy given for any indication for the duration (including follow up) of the trial.
Evidence of residual functioning beta-cells (peak serum C-peptide level > 0.2nmol/L in the MMTT test).	Use of any hypoglycaemia agents other than insulin, for more than 6 weeks, at any time prior to trial entry, including SGLT2 inhibitors.
Positive for at least one islet autoantibody (GAD, IA-2, ZnT8).	Use of inhaled insulin.
Body weight < 100kg.	Known alcohol abuse, drug abuse.
Willing to record all insulin doses and blood glucose levels required for monitoring during the study, including reporting any hypoglycaemic events.	Evidence of active Hepatitis B, Hepatitis C, HIV or considered by the investigator to be at high risk for HIV infection.
Willing to provide dried blood spot (DBS) samples.	Significant systemic infection during the 6 weeks before the first dose of the IMP (e.g. infection requiring hospitalisation, major surgery, requiring IV antibiotic treatment). Other infections e.g. glandular fever, bronchitis, sinusitis, cellulitis, or urinary tract infections must be assessed on a case-by-case basis by the investigator to assess whether they are serious enough to warrant exclusion or delay to inclusion.
Willing to wear the FreeStyle Libre Flash Glucose Monitor (FGM) device at least two weeks prior to a study visit.	History of current or past active tuberculosis (TB) infection and no latent tuberculosis. Active TB will be assessed using a **mandatory** chest x-ray and one of the following: a) blood-based test; b) the Mantoux skin test.
Willing to complete a diary and quality of life questionnaires.	Any live immunisations for 1 month prior to trial entry.

For peer review only - http://bmjopen.bmj.com/site/about/guidelines.xhtml

Willing to consent to remote follow up via health records and telephone contact.	Previous use of any other investigational drug within the 3 months prior to the first dose and/or intent on using any investigational drug for the duration (including follow up) of the trial.
Female participants have a negative urine test for pregnancy; all participants must agree to use adequate contraception if they become/are sexually active (hormonal based contraception, double barrier contraception, abstinence) until 4 months following the date of their final treatment of IMP.	Recent (within 3 months) involvement in other research studies, which, in the opinion of investigators, may adversely affect the safety of the participants or the results of the study.
	Significantly abnormal laboratory results during the screening period, other than those due to T1D.
	Prior allergic reaction, including anaphylaxis, to any component of the IMP product.
	Prior allergic reaction, including anaphylaxis, to any human, humanised, chimeric or rodent antibody treatment.
	Any major planned surgery scheduled within the 30 day period prior to the first drug dose or anticipating requiring major surgery during the study period.
	Any other medical condition or treatment that, in the opinion of investigators, could affect the safety of the participant's participation or outcomes of the study, including malignancy, immunocompromised states and autoimmune conditions.
	Participants or parents/carers who lack the capacity to comply with trial requirements.

Table 2 – Trial eligibility criteria

For peer review only

Randomisation and blinding

Minimisation by age (12-15 vs 16-18 years respectively) and screened peak C-peptide levels (0.2 - 0.7 vs > 0.7 nmol/L) will be used to ensure balance between treatment groups. These variables are important prognostic factors and need to be evenly distributed between the groups. The baseline c-peptide cut off of 0.7nmol/L was selected to correspond with Lachin 2001. (14)

The treatment:placebo ratio will be 2:1 to promote recruitment and to provide additional data on drug safety. The minimisation algorithm and randomisation list will be provided by Sealed Envelope Ltd (https://sealedenvelope.com) and accessed by sites using an online randomisation system which was validated prior to use by statisticians in Swansea Trials Unit (STU). The system will email a randomisation code to designated site personnel including Pharmacy who will cross-reference it with a code break list to determine the allocation.

Dosage and regimen of placebo and ustekinumab will be matched. Only staff preparing the blinded syringe will be unblinded at sites. Participants, research staff and the trial office remain blinded, with only limited independent researchers at (STU managing the code break list and any IMP-related queries from Pharmacies.

Emergency unblinding will be managed by Sealed Envelope Ltd. If emergency unblinding is delayed, the treating clinician should treat the patient as if ustekinumab has been given.

Trial assessments

An overview of the trial procedures are listed in Table 3.

For peer review only - http://bmjopen.bmj.com/site/about/guidelines.xhtml

	Screening (SC) [1]		Dose							Follow up
			1	2	3	4	5	6	7	
**Visit**	**SC1**	**SC2**	1	2	3	4	5	6	7	8
**Week**	Approx. -2		0	4	12	20	28	36	44	52
**Window allowed**			≤100d of clinical diagnosis and ≤37d of SC2	+/-1 week						
Consent	X									
Medical History	X									
Physical exam	X		X		X		X			X
Concomitant medication (D)	X	X	X	X	X	X	X	X	X	X
Weight	X	X	X		X		X			X
Height	X		X		X		X			X
Vital signs	X	X	X		X		X			X
TB tests [2]	X									
Adverse events (including hypoglycaemia) (D)		X	X	X	X	X	X	X	X	X
Blood draw [3, 4]	X	X	X	X	X		X			X
Urine collection [5]	X	X	X	X	X	X	X	X	X	X
Dried blood spot review		X	X	X	X	X	X	X	X	X
Download blood glucose monitoring data			(X)	X	X	(X)	X	(X)	X	X
Glycaemic control (as part of routine care)			X	X	X	X	X	X	X	X
Insulin dose usage (D)		X	X	X	X	X	X	X	X	X
PROMs (adolescent & parent)		X					X			X

Table 3: Schedule of events at sites

Table key: (X) = optional data download; (D) = data from diary

[1] *Screening visits may be combined*

[2] *Chest x-ray AND either a i) blood test (T spot / quantiferon) or ii) Mantoux test*

[3] *Safety bloods - Full blood count; urea, electrolytes and creatinine; liver function tests (total bilirubin, total protein, albumin, AST (SGOT), SGPT (ALT), alkaline phosphatase; thyroid stimulating hormone; immunoglobulins (G, A, M); calcium; magnesium, phosphate, lipid profile (total cholesterol, LDL, HDL, triglyceride), HbA1c. For screening only, we also request HIV and Hepatitis B and C and TB testing.*

[4] *Bloods for Research laboratories:* ***Diabetes Research Unit Cymru (DRUC)*** *= MMTT, Islet autoantibodies, HbA1c, exocrine enzymes, proinsulin;* ***Kings College London (KCL)*** *= T cell assays, Flow-cytometry profiles of leucocyte populations, Cytokine production by CD4 and CD8 T cells;* ***Royal Devon & Exeter Hospital*** *= glucagon and somatostatin levels;* ***University of Bristol*** *= cell free DNA;* ***commercial company*** *= pharmacokinetics analysis.*

[5] *Urine samples are collected for pregnancy testing (females), Urinalysis for pH, blood and protein by dipstick urinalysis and laboratory analysis for albumin/creatinine ratio. We also collect a sample as part of the MMTT for DRUC.*

Further details are provided below for each assessment contributing towards the objectives.

Mixed-meal tolerance test (MMTT)

Secretion of C-peptide will be assessed for the primary outcome measure of the trial using a MMTT at screening and week 52. We also conduct an MMTT at week 28 to address a secondary objective.

For peer review only - http://bmjopen.bmj.com/site/about/guidelines.xhtml

For peer review only

Participants will fast from midnight and the MMTT will be started between 7-11AM if their blood glucose prior to arriving is between 4.0 and 11.1mmol/L (inclusive). If it is <4mmol/L, the test will be postponed to a different day. If the value is >11.1 mmol/L the participant will be advised to take an appropriate correction bolus of very short acting insulin. The test may be postponed if the blood glucose is not in range after 2 hours.

The participant will be asked to drink a standardised liquid meal provided by the trial - Ensure Plus ™ 6 ml/kg (Maximum 360ml). This must be ingested within 5 minutes.

Blood glucose measurements will be taken prior to, and at the end of, the MMTT. The participant will void their bladder and urine will be collected at the end of the MMTT (at 120 minutes). Venous blood samples will be collected for measurement of C-peptide at time 0, 15, 30, 60, 90 and 120 minutes. Blood samples for mechanistic work will be taken at time 0.

Glucose monitoring

All participants will be provided with an Abbott FreeStyle Libre™ blood glucose monitoring system. Participants are expected to wear a sensor for at least 2 weeks prior to each study visit and will be advised to read their measurements at least 4-7 times a day. Anonymised data will be sent electronically to the Trial Office.

Hypoglycaemia

Participants will be advised by the research staff to record in a trial diary any hypoglycaemia symptoms between each study visit. This will be compared with glucose monitoring data. Participants will be asked to record a finger-prick blood glucose in the diary any time hypoglycaemic symptoms occur, even if the glucose monitor sensor is also being worn. A medic will categorise all hypoglycaemic events recorded in the diary according to ADA Guidelines.(15, 16)

Dried blood spot (DBS) measurements

DBS sampling will be carried out at home by the participant weekly from screening until week 28 and then monthly up to month 12 for the measurement of C-peptide. Blood samples will be obtained by finger prick and placed onto filter paper cards (Perkin Elmer). Samples will be provided before the first meal of the day, and one 60 minutes afterwards. Patients will be asked to withhold their pre-meal insulin until after the second DBS samples have been taken.

Insulin dose

Mean daily insulin use will be calculated over 7 consecutive days during the 2 weeks preceding all visits and participants will be asked to record all insulin usage in their diary during those 2 weeks. This value will be calculated in units of IU/kg/day. Where data from consecutive days are not available, the three days closest together will be used.

Body weight and BMI (clinical care measurement)

Body weight and height will be recorded at site visits and the most recent weight recorded will be used to calculate drug dosages for forthcoming treatment visits. Body mass index will be calculated as standard: weight (kg)/ [height (m)]^2.

Patient and parent reported outcome measures (PROMS)

Quality of Life for participants and their parent/carer will be assessed at screening, and weeks 28 and 52 by validated questionnaires: the Hypoglycaemia Fear Survey – HYPOFEAR;(17, 18) Diabetes Treatment Satisfaction Questionnaire for inpatients – DTSQ;(19) Paediatric Quality of Life inventory – PedsQL ™ Copyright 1998 JW Varni, PhD (generic core scale (20, 21) and diabetes-specific (22, 23) modules).

The questionnaires will be completed during the latter stages of the MMTT whilst the participant and parent are waiting for the end of the test. Participant and parent will be encouraged not to discuss their responses with each other.

For peer review only - http://bmjopen.bmj.com/site/about/guidelines.xhtml

Glycaemic control

Glycaemic control will be maintained according to clinical guidelines with the support of the participant's local diabetes clinical care team. HbA1c will be measured as per the study schedule based on the local laboratory results with a target value set according to 2015 NICE guidelines (24) in agreement with the participant and their clinical care team. Where this target is not met, advice will be given as clinically required.

Urine C-peptide/creatinine ratio (UCPCR)

Urine C-peptide/creatinine ratio will be measured from the 120 minute urine sample taken during the MMTT at screening, weeks 28 and 52. We selected this to determine whether it could be used as an alternative non-invasive test for future trials based on successes in other trials. (25, 26)

HbA1c

HbA1c will be tested in the local NHS laboratories of the study sites to guide clinical care. A blood sample will also be taken at weeks 0, 12, 28 and 52 for measurement of HbA1c using an HPLC method.

Immunological changes (mechanistic study)

Changes in immune mechanistic parameters including IL-17 and IFN-gamma production, phenotypes and function of CD4+ and CD8+ T cells will be assessed by flow cytometry immunophenotyping, Fluorospot and other immune assays, such as Luminex, at screening or week 0 (as baseline), and week 12, 28 and 52, using primarily overnight blood samples and also cryopreserved peripheral blood mononuclear cells (PBMC).

Changes in IL-17 and IFN-gamma production will be measured in both agnostic and antigen-specific manner, where for the latter T cell responses will be determined in response to antigens or peptides derived from islet antigens.

Long term follow-up assessments

We will record weight and height, insulin doses over a two week period, severe hypoglycaemia events and HbA1c levels at time points closest to weeks 78 and 104 which also coincide with a routine clinic visit. The data will be sourced from the medical records where possible and from the participant using a short questionnaire.

We also seek consent to have two additional DBS cards completed at the corresponding time points and an anonymised copy of the glucose monitor data for the two weeks prior to the time points matching the clinic visits.

TRIAL TREATMENTS

Ustekinumab (Stelara®)

Ustekinumab is a fully human IgG1k monoclonal antibody (mAb) supplied by the marketing authorisation holder Janssen-Cilag Ltd (EU/1/08/494/002). It is supplied as sterile single use 2ml glass vials containing 0.5 ml of solution with 45mg of ustekinumab for injection. Section 4.8 of the Summary of Product Characteristics (SmPC) for STELARA® (https://www.medicines.org.uk/emc/product/4413/smpc) dated 22 March 2018 will be used as the Reference Safety Information (RSI) for pharmacovigilance purposes. It was assessed by the MHRA as part of the original approvals process.

The SmPC has been updated three times so far. However, there were no significant change to the safety parameters of the trial so the original version continues to be used.

For peer review only - http://bmjopen.bmj.com/site/about/guidelines.xhtml

Placebo

Saline in the form of Sodium Chloride 0.9% w/v solution for injection will be used as the placebo. Any brand of saline with a marketing authorisation in the UK can be used for this trial. A representative SmPC will be used to represent all saline (marketing authorisation number PL 02848/0157).

Discontinuation / modification of drug dosing

Drug dosing will only be altered in response to a change in body weight as per the protocol which states 2mg/Kg ≤ 40Kg or 90mg if >40Kg.

WITHDRAWALS

The Principal Investigator or participant (or parent/carer if the participant is <16y) can opt to discontinue treatment for any reason. The participant (and parent if <16y) will be asked to remain in the trial for sample and data collection purposes only. They have the right to withdraw completely without giving a reason.

Oversight committees and the Sponsor can request the withdrawal of a participant(s) or to terminate the trial.

Exceeding the timeframe for receiving medication may also result in withdrawal from treatment.

SAFETY REPORTING

The risk of major adverse unexpected events is anticipated to be low. Ustekinumab has a marketing authorisation in the age group being studied for other indications. The available SmPC describes all essential information for the use of the medicine, and the qualitative and quantitative information on benefits and risks. Participants being exposed to ustekinumab are a different disease population from those described in the SmPC. In addition, the dose used in this trial is higher than that currently licensed for psoriasis in adolescents, although it (and higher doses) have been used in adults with both psoriasis and Crohn's disease.

Hypoglycaemic events are common in this population and may not necessarily be IMP-related. Hypoglycaemia rates are an important secondary outcome, as it is anticipated that these should be reduced by ustekinumab if it is effective. Hypoglycaemic events are recorded specifically for this trial separately from other adverse events (AEs) because they require medical assessment according to ADA Guidelines (15, 16).

A review of AEs will be performed at all visits (participant-reported) and using blood and urine samples at screening and 0, 12, 28 and 52 weeks. A urine pregnancy test will be completed on all females at all trial visits. Principal Investigators will be expected to assess any values outside the laboratory reference range for clinical significance.

Hypoglycaemia and diabetic ketoacidosis are considered expected for newly diagnosed T1D patients. If the event leads to death, this will be considered unexpected.

Any pregnancies for female participants or the pregnant partners of male participants must be reported immediately. Pregnant participants will be withdrawn from treatment and asked to provide consent to follow up the pregnancy until the child is 12 months old.

POST TRIAL CARE

Following completion of their trial participation, participants will be kept informed of ongoing trial developments including final outcomes following statistical analyses. Should participants be concerned about implications arising from their trial participation, they will be asked to discuss these

For peer review only

For peer review only - http://bmjopen.bmj.com/site/about/guidelines.xhtml

with their local clinicians. Senior members of the trial team will be available for further advice should the local clinician require.

Once the trial is complete, following unblinding, individual participants and their local clinicians will be informed by letter on request as to which arm of the trial they were randomised to. After completing the trial, clinical care and follow-up will be provided by the participant's local diabetes care team. Ustekinumab will not be available for ongoing therapy.

STATISTICS AND DATA ANALYSIS

Sample size considerations

The power calculation closely follows Lachin (14) based on data for children and young adolescents aged 13-17 years as well as the T1DAL study in 12-35y olds (27). A sample size of 66 apportioned in a 2:1 ratio has a greater than 85% power to detect a 0.2nmol/L difference between the 2-hour MMTT mean AUC C-peptide values of the intervention and placebo arms which are assumed to be 0.5 and 0.3 (nmol/L) respectively at 12 months. Seventy-two participants (48 ustekinumab :24 placebo) will be recruited to allow for approximately 10% loss to follow-up.

Data analysis

Data cleaning and preparation processes will be carried out prior to final analysis. A statistical analysis plan (SAP) approved by the Data Safety Monitoring Board (DSMB) will be followed.

All participants enrolled will be followed up and included unless they withdraw from the study before the administration of the first dose. An intention to treat (ITT) analysis will be carried out. Per protocol analysis of the primary outcome will also be carried out alongside the ITT analysis if deemed necessary by the Trial Steering Committee (TSC).

The primary data analysis will be the application of analysis of covariance to the 12-month recorded AUC mean values of C-peptide taking into account the baseline values of these measures and using transformations as suggested by Lachin (14). The analysis will be adjusted by important covariates such as gender, age at recruitment, baseline insulin use and glycaemic control.

For the secondary outcomes including the mechanistic and questionnaire studies we will evaluate the various outcomes using the most appropriate statistical approach i.e. binomial or logistic regression for binary outcomes, Poisson or related count outcome models for number of events/objects and linear models for continuous outcomes. Where necessary, mixed or multilevel models will be used to account for correlation within observations.

No interim analysis is planned. No subgroup analysis is planned. Should there be substantial non-fidelity to allocated treatment, a per-protocol analysis for the primary outcome will be considered after approval by the TSC.

Efficacy analyses will be adjusted by gender, age and baseline test values. Safety analysis will not be adjusted.

Interim analysis on safety data only will be conducted if requested by TSC/ DSMB. Decision criteria based on safety as part of a guideline for early stopping or other adaptations will be set by TSC with input from DSMB.

Every attempt will be made to minimise missing data, encouraging participants to provide week 52 data even if they are no longer taking the interventional medication. Patterns and level of missing data will be examined. Multiple imputation will be considered if required, if there are more than 5% and less than 10% (>3 and <7 participant) missing.

For peer review only

For peer review only - http://bmjopen.bmj.com/site/about/guidelines.xhtml

DATA MANAGEMENT

Source documents produced for this trial will be filed with the participant's medical records. Source data will be entered into trial-specific database of electronic Case Report Forms (eCRFs) at the end of each trial visit within a site agreed timespan. These eCRFs will be coded with the participants study number and will not include patients' names and addresses and will conform to General Data Protection Requirements (GDPR). This database (MACRO v4.7 Elsevier Ltd 2017) will be hosted on a Swansea University server with back up and restoration procedures in place. All paper CRFs can be found by logging into the trial website and entering the password.

The trial database will be managed and operated as required by GCP. The site investigator or delegate will record all study data using the trial specific electronic database provided by STU. All data will be handled and stored in accordance with GDPR, Data Protection Act and applicable legislation.

Data will be checked according to the trial Data Management Plan and queries will be generated and sent to the site investigator for response using the database.

Data from laboratories and the anonymised glucose monitoring and diary data from patients will be securely transferred to the Trial Office.

Remote data collection after week 52 will be done using the REDCap™ database with links to participant questionnaires emailed by site researchers. No identifiable data is collected in the database during remote follow up.

The CI and trial statistician will have access to the final dataset for analysis. Should PIs or others require access to the final dataset this will require approval by the TMG, TSC and Sponsor.

The trial data will be held in a data repository, the location of which is still being negotiated.

MONITORING

Monitoring of this trial to ensure compliance with Good Clinical Practice (GCP) and scientific integrity will be conducted by STU via central and on-site monitoring as per the Trial Monitoring Plan.

This will include 100% central monitoring of all primary outcome data, with site initiation and closedown visits for all sites, and a minimum of one monitoring visit during the recruitment period to complete 100% Source Data Verification on primary outcome data. In addition, the trial office will facilitate monitoring by local R&D departments at any of the trial sites, should this be requested.

DISSEMINATION

A Publication Plan will be developed to organise the outputs from this trial. Outputs will be disseminated using highly accessed, peer-reviewed medical journals and will be presented at conferences.

Authorship will be agreed upon by the CI, PIs, and members of the TMG and will follow the guidance provided by the International Committee of Medical Journal Editors.

PATIENT AND PUBLIC INVOLVEMENT

We recruited a panel of children with T1D to help us develop a short recruitment video.

We recruited six Patient and Public Involvement (PPI) representatives, two for each committee (TMG, DSMB and TSC). All PPI representatives are either parents of children with T1D or have T1D themselves. Our PPI representatives assist in the development of participant facing documentation, support applications for approvals and will review and help to disseminate our results

For peer review only - http://bmjopen.bmj.com/site/about/guidelines.xhtml

STUDY MANAGEMENT

The Trial Office is based at STU, with the Chief Investigator, paediatric lead and adult lead all working at Cardiff University.

The sponsor of the trial is Cardiff University. The sponsor can be contacted at resgov@cardiff.ac.uk.

The sponsor has arranged appropriate insurance and indemnity to meet the potential legal liability for harm to the participants arising from the design or management of the trial for negligent harm. In addition, the trial health professionals hold substantive or honorary NHS contracts, giving them the protection of the appropriate NHS clinical negligence arrangements

TRIAL COMMITTEES

The trial oversight committees are the DSMB and TSC who will meet biannually. They comprise of clinical experts, a statistician and public and patient involvement (PPI) representatives and each work to a pre-agreed charter. The DSMB to provide ethical and safety reviews (including the assessment of adverse events and protocol deviations) and the TSC will have general oversight of the trial to ensure recruitment, treatment and follow up visits are safe and providing the relevant data, and that the protocol is being adhered to, based on DSMB recommendations.

The TMG consists of the trial team, independent advisors and PPI representatives and meets at least quarterly and provides a forum to discuss trial progress with key members and the content of reports to, and responses from, the oversight committees.

REGULATORY APPROVALS

Ethical approval for the trial protocol was received on 18/09/18 from Wales REC 3 – reference 18/WA/0092. Regulatory approval from the Medicines and Healthcare products Regulatory Agency (MHRA) was received on 26/06/18. Site-specific capability and capacity will be sought for the trial. Amendments to REC-approved documentation will not used until approval from the relevant regulatory authorities is in place.

AUTHORS' CONTRIBUTIONS

Authors MA, NB, JBM, AB, WYC, CD, GD, GF, JG, SH, GH, HH, SL, SMJ, AM, RS, DT, TT, KT and JY played a significant role in the development of the protocol. Authors SMJ and AB are our PPI representatives and review the protocol and other trial related documentation. CD is the Chief Investigator whilst JG is the paediatric T1D lead and DT is the adult T1D lead. CD and DT are the joint senior authors of the paper.

FUNDING STATEMENT

This project (project reference 16/36/01) is funded by the Efficacy and Mechanism Evaluation (EME) Programme, an MRC and NIHR partnership. The views expressed in this publication are those of the author(s) and not necessarily those of the MRC, NIHR or the Department of Health and Social Care. Additional funding for mechanistic laboratory tests has been provided by JDRF (Juvenile Diabetes Research Foundation) International Award 3-SRA-2018-629-S-B.

COMPETING INTERESTS STATEMENT

At the time of writing, no financial conflict or any other relevant connection or shared interest was declared for the CI, committee members or anyone involved in the management of the trial.

For peer review only - http://bmjopen.bmj.com/site/about/guidelines.xhtml

Janssen-Cilag will supply ustekinumab for the trial at no cost, while Abbott Diagnostics will supply glucose monitoring devices. Neither have been granted, nor sought to obtain, any financial or in kind advantage or reward for supplying the products. However, glucose sensors will be purchased from Abbott at cost price for the duration of the trial. Both parties will be contracted by the sponsor.

License statement

I, the Submitting Author has the right to grant and does grant on behalf of all authors of the Work (as defined in the below author licence), an exclusive licence and/or a non-exclusive licence for contributions from authors who are: i) UK Crown employees; ii) where BMJ has agreed a CC-BY licence shall apply, and/or iii) in accordance with the terms applicable for US Federal Government officers or employees acting as part of their official duties; on a worldwide, perpetual, irrevocable, royalty-free basis to BMJ Publishing Group Ltd ("BMJ") its licensees and where the relevant Journal is co-owned by BMJ to the co-owners of the Journal, to publish the Work in BMJ Open and any other BMJ products and to exploit all rights, as set out in our licence.

For peer review only

For peer review only - http://bmjopen.bmj.com/site/about/guidelines.xhtml

Figure 1: Trial flowchart

For peer review only

For peer review only - http://bmjopen.bmj.com/site/about/guidelines.xhtml

REFERENCES

1. Wood JR, Miller KM, Maahs DM, Beck RW, DiMeglio LA, Libman IM, et al. Most youth with type 1 diabetes in the T1D Exchange Clinic Registry do not meet American Diabetes Association or International Society for Pediatric and Adolescent Diabetes clinical guidelines. Diabetes care. 2013;36(7):2035-7.

2. Centers for Disease Prevention. Diabetes death rates among youths aged </= 19 years--United States, 1968-2009. MMWR Morbidity and mortality weekly report. 2012;61(43):869-72.

3. Centre for Disease Prevention. Diabetes death rates among youths aged ≤ 19 years--United States, 1968-2009. MMWR Morb Mortal Wkly Rep. 2012;61(43):869-72.

4. Wilcox NS, Rui J, Hebrok M, Herold KC. Life and death of β cells in Type 1 diabetes: A comprehensive review. J Autoimmun. 2016;71:51-8.

5. Lachin JM, McGee P, Palmer JP, Group DER. Impact of C-peptide preservation on metabolic and clinical outcomes in the Diabetes Control and Complications Trial. Diabetes. 2014;63(2):739-48.

6. Sorensen JS, Johannesen J, Pociot F, Kristensen K, Thomsen J, Hertel NT, et al. Residual beta-Cell function 3-6 years after onset of type 1 diabetes reduces risk of severe hypoglycemia in children and adolescents. Diabetes care. 2013;36(11):3454-9.

7. Effect of intensive therapy on residual beta-cell function in patients with type 1 diabetes in the diabetes control and complications trial. A randomized, controlled trial. The Diabetes Control and Complications Trial Research Group. Ann Intern Med. 1998;128(7):517-23.

8. Nathan DM, Cleary PA, Backlund JY, Genuth SM, Lachin JM, Orchard TJ, et al. Intensive diabetes treatment and cardiovascular disease in patients with type 1 diabetes. The New England journal of medicine. 2005;353(25):2643-53.

9. Writing Team for the Diabetes C, Complications Trial/Epidemiology of Diabetes I, Complications Research G. Sustained effect of intensive treatment of type 1 diabetes mellitus on development and progression of diabetic nephropathy: the Epidemiology of Diabetes Interventions and Complications (EDIC) study. Jama. 2003;290(16):2159-67.

10. Marwaha AK, Tan S, Dutz JP. Targeting the IL-17/IFN-γ axis as a potential new clinical therapy for type 1 diabetes. Clin Immunol. 2014;154(1):84-9.

11. Greenbaum CJ, Schatz DA, Haller MJ, Sanda S. Through the fog: recent clinical trials to preserve β-cell function in type 1 diabetes. Diabetes. 2012;61(6):1323-30.

12. American Diabetes Association. Standards of medical care in diabetes--2014. Diabetes care. 2014;37 Suppl 1:S14-80.

13. American Diabetes Association. 2. Classification and Diagnosis of Diabetes: Standards of Medical Care in Diabetes-2018. Diabetes care. 2018;41(Suppl 1):S13-S27.

14. Lachin JM, McGee PL, Greenbaum CJ, Palmer J, Pescovitz MD, Gottlieb P, et al. Sample size requirements for studies of treatment effects on beta-cell function in newly diagnosed type 1 diabetes. PLoS One. 2011;6(11):e26471.

15. Seaquist ER, Anderson J, Childs B, Cryer P, Dagogo-Jack S, Fish L, et al. Hypoglycemia and diabetes: a report of a workgroup of the American Diabetes Association and the Endocrine Society. Diabetes Care. 2013;36(5):1384-95.

16. Workgroup on Hypoglycemia AeDA. Defining and reporting hypoglycemia in diabetes: a report from the American Diabetes Association Workgroup on Hypoglycemia. Diabetes Care. 2005;28(5):1245-9.

17. Gonder-Frederick L, Nyer M, Shepard JA, Vajda K, Clarke W. Assessing fear of hypoglycemia in children with Type 1 diabetes and their parents. Diabetes Management. 2011;1(6):627-39.

18. Gonder-Frederick L, Schmidt KM, Vajda KA, Greear ML, Singh H, Shepard JA, et al. Psychometric Properties of the Hypoglycemia Fear Survey-II for Adults With Type 1 Diabetes. Diabetes Care. 2011;34:801-6.

19. Bradley C. The Diabetes Treatment Satisfaction Questionnaire: DTSQ. Handbook of Psychology and Diabetes: a guide to psychological measurement in diabetes research and practice. Switzerland: Harwood Academic Publishers; 1994.

For peer review only

For peer review only - http://bmjopen.bmj.com/site/about/guidelines.xhtml

20. Varni JW, Seid M, Rode CA. The PedsQL: measurement model for the pediatric quality of life inventory. Med Care. 1999;37(2):126-39.

21. Varni JW, Seid M, Kurtin PS. PedsQL 4.0: reliability and validity of the Pediatric Quality of Life Inventory version 4.0 generic core scales in healthy and patient populations. Med Care. 2001;39(8):800-12.

22. Varni JW, Delamater AM, Hood KK, Raymond JK, Chang NT, Driscoll KA, et al. PedsQL 3.2 Diabetes Module for Children, Adolescents, and Young Adults: Reliability and Validity in Type 1 Diabetes. Diabetes Care. 2018;41(10):2064-71.

23. Varni JW, Delamater AM, Hood KK, Raymond JK, Chang NT, Driscoll KA, et al. Pediatric Quality of Life Inventory (PedsQL) 3.2 Diabetes Module for youth with Type 2 diabetes: reliability and validity. Diabet Med. 2019;36(4):465-72.

24. National Institute for health and Care Excellence. Diabetes (type 1 and type 2) in children and young people: diagnosis and management. In: Excellence NIfHaC, editor. www.nice.org.uk/guidance/ng182015.

25. Tatovic D, Luzio S, Dunseath G, Liu Y, Alhadj Ali M, Peakman M, et al. Stimulated urine C-peptide creatinine ratio vs serum C-peptide level for monitoring of β-cell function in the first year after diagnosis of Type 1 diabetes. Diabet Med. 2016;33(11):1564-8.

26. Besser RE, Ludvigsson J, Jones AG, McDonald TJ, Shields BM, Knight BA, et al. Urine C-peptide creatinine ratio is a noninvasive alternative to the mixed-meal tolerance test in children and adults with type 1 diabetes. Diabetes Care. 2011;34(3):607-9.

27. Rigby MR, DiMeglio LA, Rendell MS, Felner EI, Dostou JM, Gitelman SE, et al. Targeting of memory T cells with alefacept in new-onset type 1 diabetes (T1DAL study): 12 month results of a randomised, double-blind, placebo-controlled phase 2 trial. Lancet Diabetes Endocrinol. 2013;1(4):284-94.

For peer review only

For peer review only - http://bmjopen.bmj.com/site/about/guidelines.xhtml

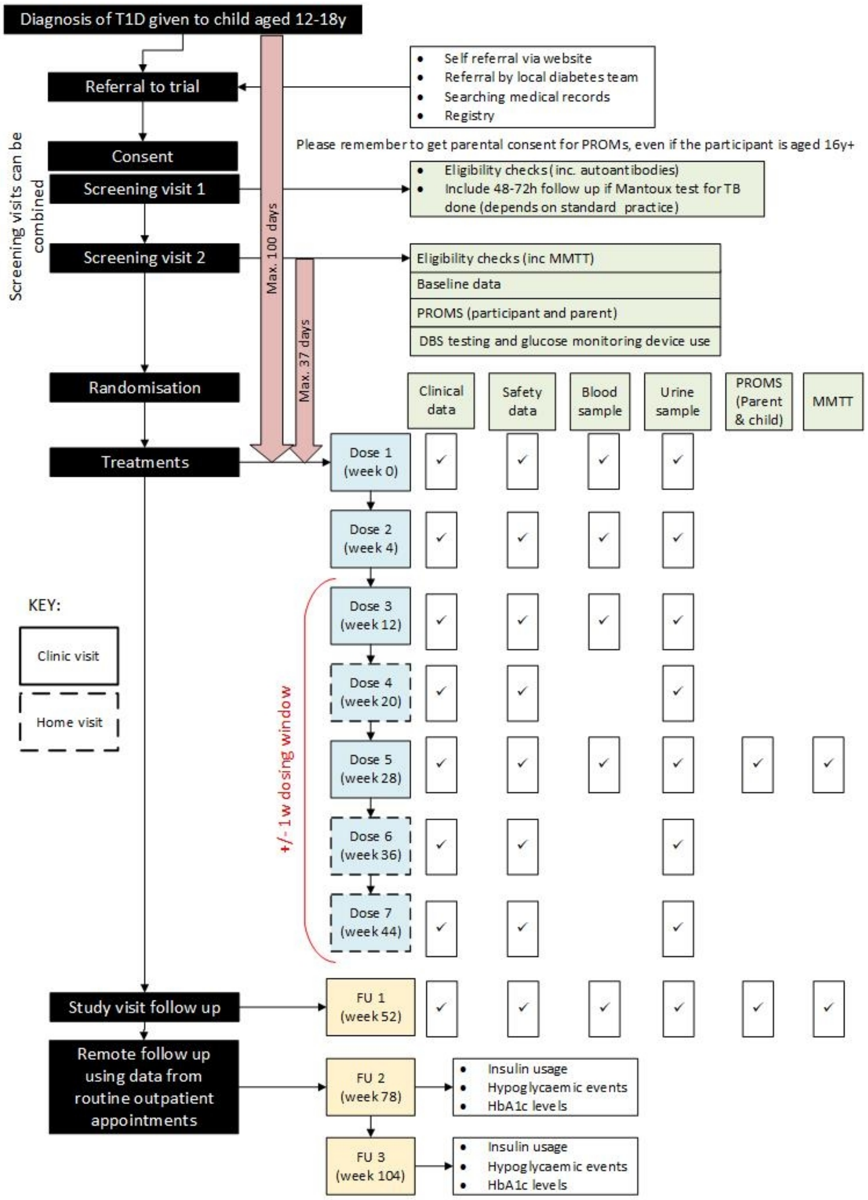

Figure 1: Trial flowchart

197x273mm (96 x 96 DPI)

A research study to see if the medicine Ustekinumab can make diabetes easier to manage

CONTACT DETAILS FOR STUDY TEAM:

NURSE:

DOCTOR:

EMERGENCIES:

FOR YOUNG PEOPLE AGED 12-15 YEARS OLD

We would like you to help us with our research study. Please read this information carefully and talk to your parent or carer about the study. Ask us if there is anything that is not clear or if you want to know more. Take time to decide if you want to take part. It is up to you if you want to do this. If you decide not to take part, then that is fine, you will be looked after by your doctors just the same.

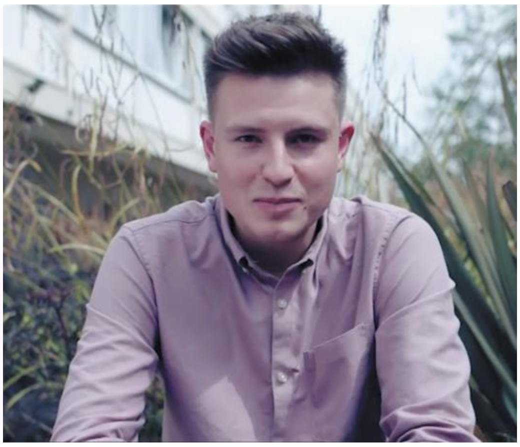

Please look at our video explaining the trial at www.type1diabetesresearch.org.uk/current-trials. The blue box below and the video contains the key points about the study. If you would like to know more, please read the rest of this leaflet.

KEY POINTS ABOUT THE STUDY:

- We want to see if the study medicine, **Ustekinumab**, can help people with Type 1 Diabetes (the type you have). The medicine works by "protecting" some of the cells in the body that still produce insulin to help make diabetes easier to manage.
- The study will involve injections of the study medicine or a placebo (a "dummy" medicine that has no effects) every 1-3 months. These injections are given under the skin like insulin injections and will be done by the study doctor or nurse. Neither you nor the research team will know if you are receiving the study medicine or the placebo.
- The medicine is already being used to treat other illnesses quite safely.
- You will be asked to come into your local hospital or research centre for 10 study visits over a 15 month period, but where possible this will be on the same day as your routine hospital visits and 3 of the visits can sometimes be done by a research nurse at your home.
- We will ask you to provide extra blood and urine (wee) samples to check that it is OK for you to take part in the study and to check your health during the study. We will also use them to do some special tests in laboratories.
- You will receive a small gift voucher for each study visit you come to.
- You will wear a flash glucose monitor ("Freestyle Libre") on your arm to check your blood glucose levels for 2 weeks before each study visit. You can keep this for use at home for the rest of the time you are in the study if you want to.
- You can stop taking part in the study at any time and do not have to give a reason why.

For peer review only - http://bmjopen.bmj.com/site/about/guidelines.xhtml

WHY IS THIS STUDY BEING DONE?

This study is being done to see if a medicine called **Ustekinumab** can help to "protect" the cells in the body that produce insulin in young people recently diagnosed with Type 1 Diabetes (or T1D). At the time of diagnosis, most people your age with diabetes have some of their insulin-producing cells still working. It usually takes between 1 and 5 years before they stop working completely due to damage by the immune system. Sometimes these last few working cells can make enough insulin to make blood glucose levels stable and easier to control — this is called the "Honeymoon period". This period is only temporary and doesn't last. Ustekinumab, the study medicine, may make this period last longer by reducing the effects of the immune system on the insulin-producing cells in the pancreas.

WHY HAVE I BEEN ASKED TO TAKE PART?

You have been chosen because you are aged 12 - 18 years old and have recently been diagnosed with T1D.

DO I HAVE TO TAKE PART?

No. It is completely up to you whether or not you take part and you can always change your mind at any time. If you decide you don't want to take part, that's OK. Nobody will be upset and the medical care you get from your diabetes health care team will not be affected. You do not need to decide if you want to take part straight away. You can take your time and talk about the study with your family, friends and the study team if you want to. You need to let us know within 6 weeks of being diagnosed with diabetes, so that we can start the treatment early enough.

If you are interested in this study then you and your parent/carer should let your doctor or nurse know. Someone from the research team will get in touch to explain more about the study and answer any questions you have. You will be asked to sign a form to say that you are happy to take part. Your parent/carer will also sign a form to say that they agree too.

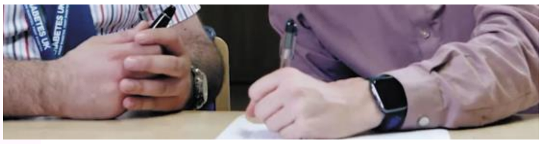

WHAT HAPPENS ONCE I HAVE AGREED TO TAKE PART?

SCREENING VISITS

Once the forms have been signed by you and your parent/carer we will do checks - "screening" - to see if you are suitable for the study. There are 2 screening visits during which we will do some tests to tell us about your health and your diabetes.

The first screening visit involves:

- A general health check by a doctor to make sure you can take part.
- We will take some blood samples (this will range from half a tablespoon to two tablespoons, depending on how much the hospital laboratory needs) from your arm.
- We will check whether you have an infection called TB (tuberculosis) or any viral infections which would stop you from taking part.
- We will also ask you to wee into a container so that we have a urine sample to test your kidneys and for girls, to make sure you're not pregnant.

The second screening visit involves:

- A **mixed meal tolerance test** (the Milkshake test) to test how much insulin your body still makes (see www.type1diabetesresearch.org.uk/current-trials). This involves coming to the hospital in the morning having not had breakfast. A small thin plastic tube will be put into a vein in your arm to take blood samples (with local anaesthetic ("numbing") cream/spray if you want it). This means that you will not need to have a needle in your arm for every blood sample we take during the test. We will need to take almost 3 tablespoons of blood at this visit in total. You then drink a flavoured drink that makes your body release insulin. Blood samples will be taken regularly through the tube over a period of 2 hours and your blood glucose is checked. During this time you can rest on a bed and can play on the internet or do other activities. At the end, the tube is removed. You then take an insulin injection depending on your blood glucose level and can leave the hospital when ready.

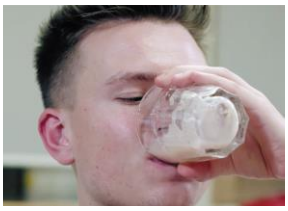

- We will ask you to complete a short questionnaire about your diabetes and how you feel. Your parent/carer will complete a similar one to you. You can ask the study nurse if you are unsure how to answer any questions.
- We will give you a free Freestyle Libre blood glucose monitor to wear at least two weeks before every visit (you can keep it on all the time if you want to).

If one of the screening tests tells us that you cannot take part, we will let you know as soon as possible.

If you and your parent/carer decide that it is easier to do both screening visits in one go, we can do this.

If the results of the screening tests are OK, then a computer will decide by chance whether you will receive the study medicine (Ustekinumab) or the placebo ("dummy" medicine).

Two thirds of young people taking part will receive the study medicine, and one third the placebo.

For peer review only - http://bmjopen.bmj.com/site/about/guidelines.xhtml

Neither, your family or the doctors and nurses will know which treatment you received until the end of the study.

STUDY VISITS

Once you enter the main study, you will be asked to come to 8 study visits over 52 weeks. Some of these visits may be on the same day as a routine hospital visit and 3 of the visits may be done by a nurse visiting you at home.

Activities at the visits may include:

- Check up by a nurse or doctor
- Urine sample tests
- Blood sample tests (between 3 and 4 tablespoons). If you feel unwell while this is being done, or you do not want to give the whole amount, the doctor or nurse can stop taking blood at any time.
- An injection of either the study medicine or the placebo. You will be asked to stay in the hospital for 1 hour after you receive the first injection so that the study team can make sure that you are OK.
- We will also download data that has been collected by a blood glucose monitoring device we will give you.
- Two study visits will involve mixed meal tolerance (Milkshake) tests.

FOLLOW UP

After the final study visit at week 52, we will check your hospital records for the next 12 months to find out how you are doing. You do not need to come into the hospital for a study visit. We may need to call you or your parent/carer up at home to check that you are OK and in good health.

WHAT ELSE WILL I BE ASKED TO DO?

You will be asked to complete a diary to record:

a) How much insulin you take in the two weeks before each study visit.
b) If you feel unwell or have to take any medicines during the study.
c) If you have any hypoglycaemic reactions (low blood glucose levels) that make you feel unwell during the study.

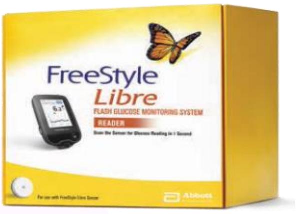

- We will ask you to test your blood glucose levels at home for at least 2 weeks before each visit using our FREE Abbott Freestyle Libre flash glucose monitoring system so you don't need to do extra finger prick tests for the trial. You will need to wear a sensor on your arm for the two weeks before the study visit if you want to take part and you can wear it every day if you want to. We will give you enough sensors to allow this for a year. We will also show you how it works.
- We will ask you to give a finger prick blood spot sample (just like a normal finger prick glucose test) before and 1 hour after the first meal of the day. This should be done once a week up for the first 28 weeks, then once a month for the next 6 months. You should do these at home with help from your parent/carer. We will show you how to do this - you prick your finger and then drop a spot of blood onto a special card.
- You will be asked to complete 3 questionnaires about your diabetes and how you are feeling at the start, middle and end of the study. Your parent/carer will also complete similar questionnaires for us.
- You will be asked not to have certain vaccinations before, during and immediately after the study.
- We will ask for permission to see your hospital records for a year after you finish your study visits to look at any changes to your insulin doses and glucose levels after stopping the treatment.
- A urine pregnancy test will be routinely done for all girls at each study visit.
- You need to be careful that you do not end up getting pregnant or making someone pregnant while you are in the study. We are asking everyone taking part to agree to use contraceptives before we can consider them for the study. Your parents/carers will be told about this requirement. Your GP or a pharmacist can advise on contraception if you don't want to discuss this with your parents/carers.

WILL THE STUDY HELP ME?

If you are in the group that receives the study medicine, it is possible that it will help your pancreas make insulin for longer. However, we cannot say this for certain until we have completed this study. During the study your diabetes will be very closely monitored. This will include regular check-ups with your local diabetes team including routine blood testing. You will have more time with the research team to discuss your diabetes and ask questions than at a normal clinic appointment.

You will be given an Abbott Freestyle Libre blood glucose monitoring system that you can use for the whole year to monitor your blood glucose levels without extra finger prick tests. You must wear this for the two weeks before the study visit if you want to take part. The treatments will stop at week 44 and you will not receive any further injections of the study drug / placebo during the trial.

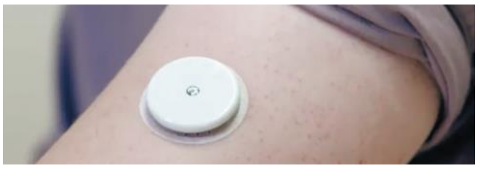

For peer review only - http://bmjopen.bmj.com/site/about/guidelines.xhtml

WHAT IF I DO NOT WANT TO TAKE PART ANYMORE?

Just tell your parents/carers to let us know. Nobody will be cross with you. You will still receive the same care from your doctors. We will still test the blood samples you have given so far unless you ask us not to.

WHAT IF THERE IS A PROBLEM OR SOMETHING GOES WRONG?

Tell your parents/carers as soon as possible if there is a problem so that they can let the study doctor or research team know and they will try to sort it out straight away. We will tell your parent/carer more about what to do if there is a problem when we talk to them about the study.

WHAT ARE THE POSSIBLE SIDE EFFECTS FOR ME IN PARTICIPATING IN THE STUDY?

You may get a bruise or a little discomfort where the needle goes in for the blood tests. We can use a cream or spray to numb the area from where we take blood if you like.

The FreeStyle Libre sensor may cause a slight rash for some people who might be allergic to the sticky part of the sensor. Please let the research team know if this happens.

During the Milkshake test, your blood glucose levels may be higher than usual because you will not have taken insulin immediately beforehand. The nurses and doctors will be available to help you make any changes to your usual insulin doses after this test.

The study medicine has not been tested in people with Type 1 Diabetes before so there may be some effects that we do not yet know about. Because the medicine acts on the immune system, there is a possibility that it could increase the risk of infections and cancer, but so far this has not been the case in people treated with this medicine for others diseases. It is also possible that you may get an allergic reaction to the treatment injection. We will ask you to stay for one hour after your first injection to check for any reactions.

We will need to do a chest x-ray. X-rays can cause damage to the body when you are older but we are only asking for one, which will hardly affect you.

If you feel ill at any time during the trial and go to your GP or the hospital, please show them the membership card you will be given so that they can contact the research team to ask about possible side effects.

WILL I RECEIVE ANY PAYMENT FOR TAKING PART?

As a thank you for you helping us with our research you will receive a £10 gift voucher for each treatment visit you come to and we'll give you a £30 gift voucher if you come to the final visit (visit 8) (that's £100 in total if you come to all visits). We will pay your parent/carer for any travel costs for attending study visits.

WHAT INFORMATION WILL YOU COLLECT AND HOW WILL IT BE KEPT PRIVATE?

We will ask for your name and contact details as well as information about your health which we get from your tests, your blood glucose monitor and your medical records. We will also ask you to complete a questionnaire at three time points. All of this information will be kept safely and nobody else will know that it is about you because your name will be removed and replaced with a study number. The people in our research team and diabetes care staff at the local hospital or research centre will know that you are taking part. We will also tell your family doctor (GP) that you are in the study if your parent/carer agrees to this.

People at the research laboratories will not know who you are when they test your samples. Your sample will be given a study number to replace your name so any study samples and data related to you will be anonymised. Cardiff University is responsible for the data we collect about you and will keep it secure for as long as is necessary.

WHAT WILL HAPPEN TO ANY SAMPLES I GIVE?

We want to test your blood and urine to better understand your diabetes and how the study medicine affects your diabetes. Samples will be tested in different research laboratories in the UK and may also be sent to special laboratories in Europe, America or Canada. These samples will not have your name on, only a study number so your identity will not be revealed.

We would like to keep any leftover blood samples in storage permanently for future research - we will ask you and your parent/carer to decide if they want you to do this.

WHAT SHOULD I DO NOW?

If you are interested in taking part, let your parents/carers know and they will get in touch with the study nurse/doctor.

Thank you for taking the time to read this information sheet and for considering taking part in this research study

For peer review only - http://bmjopen.bmj.com/site/about/guidelines.xhtml

[Insert local headers]

USTEKID

NHS National Institute for Health Research

CARDIFF UNIVERSITY PRIFYSGOL CAERDYDD

Swansea University Prifysgol Abertawe
Swansea Trials Unit Uned Dreialon Abertawe

A research study to see if the medicine Ustekinumab can make diabetes easier to manage

CONTACT DETAILS FOR STUDY TEAM:

NURSE:

DOCTOR:

EMERGENCIES:

FOR YOUNG PEOPLE AGED 16-18 YEARS OLD

We would like you to help us with our research study. Please read this information carefully and talk to your parent or carer about the study. Ask us if there is anything that is not clear or if you want to know more. Take time to decide if you want to take part. It is up to you if you want to do this. If you do not, then that is fine, you will be looked after by your doctors just the same.

Please look at our video explaining the trial at www.type1diabetesresearch.org.uk/current-trials. The blue box below and the video contains the key points about the study. If you would like to know more, please read the rest of this leaflet.

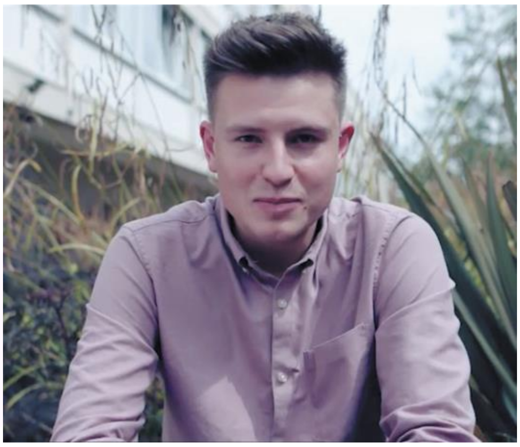

KEY POINTS ABOUT THE STUDY:

USTEKID

- We want to see if the study medicine, **Ustekinumab**, can make Type 1 Diabetes (the type you have) easier to manage. The medicine works by "protecting" some of the cells in the pancreas that still produce insulin from attack by the immune system.
- The study will involve you having an injection every 1-3 months with either the study medicine, Ustekinumab or a placebo (a "dummy medicine"). These injections are given under the skin just like insulin injections and will be done by the study doctor or nurse. Neither you nor the research team will know if you receive the study medicine or the placebo.
- The medicine is already being used to treat other illnesses quite safely. We will give you information about possible side effects before you decide if you will take part.
- You will be asked to come into your local hospital or research centre for 10 study visits over a 15 month period, but where possible this will be on the same day as your routine hospital visits. The first two will check if you are eligible to take part. Three of these visits can sometimes be done by a research nurse at home.
- We will ask you to provide extra blood and urine samples to check that it is safe for you to take part in the study and to check your health, your blood glucose levels and how your immune system is working during the study.
- At three visits, you will have blood tests over a 2 hour period to see how much insulin your body is making.
- We will provide you with a glucose monitor (Freestyle Libre) to wear for 2 weeks before each visit. You can keep the monitor for use at home for the whole time of the study.
- You will be offered a small gift voucher for each visit you come to and your travel expenses will be paid.
- You can stop taking part in the study at any time and you do not have to give a reason why.

For peer review only - http://bmjopen.bmj.com/site/about/guidelines.xhtml

WHY IS THIS STUDY BEING DONE?

This study is being done to see if a medicine called **Ustekinumab** can help to "protect" the cells in the body that produce insulin in young people recently diagnosed with Type 1 Diabetes. It is caused by the body's own immune system damaging the cells in the pancreas that make insulin. Our aim is to develop a treatment that can slow this process by targeting the immune cells causing the damage.

At the time of diagnosis, most people your age have 10-20% of their insulin-producing cells still working. It usually takes between 1 and 5 years before they stop working completely. Sometimes these last few working cells can make enough insulin to make blood glucose levels stable and easier to control – this is called the "Honeymoon period". This period is only temporary and doesn't last. Ustekinumab, the study medicine, may make this period last longer by reducing the damaging effects of the immune system on the remaining insulin-producing cells in the pancreas.

Ustekinumab is currently given to adults and teenagers with particular skin and bowel problems and it is known to be safe to use and effective at treating those conditions.

WHY HAVE I BEEN ASKED TO TAKE PART?

You have been chosen because you are aged 12 - 18 years old and have recently been diagnosed with Type 1 diabetes.

DO I HAVE TO TAKE PART?

No. It is completely up to you whether or not you take part and you can always change your mind at any time. If you are interested in this study then:

- Let us know by calling one of the people listed at the end of this information sheet.
- A member of the research team will contact you to explain more about the study and answer any questions you have.
- If you agree, you will sign a consent form and be given a copy of your signed consent form and this information sheet to keep.

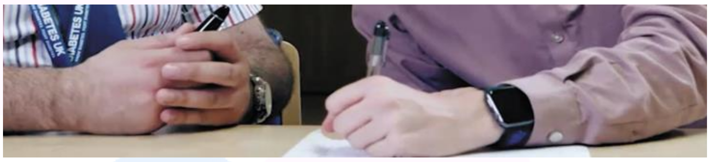

If you make a decision to take part, ***you are still free to withdraw from the study at any time without giving a reason***. This will not affect the medical care you get from your diabetes doctor in any way.

You do not need to decide if you want to take part straight away. You can take your time and talk about the study with your family, friends and the study team if you want to. You need to let us know within 6 weeks of being diagnosed with diabetes, so that we can start the treatment early enough.

WHAT HAPPENS ONCE I HAVE AGREED TO TAKE PART?

VISIT	WHAT WILL HAPPEN	WHERE AND HOW LONG WILL IT TAKE?
Screening visits	Before we can start the treatment, we need to check that you are eligible to take part in the study - this is called "screening". You will be asked to come to your local hospital or research centre to talk about the study. This is where you will be able to ask questions.<br>If you are happy to take part you will be asked to sign a consent form before we begin the screening tests which involve:<br>Screening visit 1:<br>• Doing a general health check (this includes a general examination and measuring your height, weight and blood pressure).<br>• Asking about any medicine you are taking and any illnesses you have had or still have.<br>• Taking some blood samples (between 0.5 - 2 tablespoons) from your arm to check your general health and diabetes, as well as testing for infections such as TB (tuberculosis), hepatitis and HIV. Blood volumes vary according to your hospital's local testing procedures.	Your local hospital or research centre.<br>Approximately 1 hour for the first visit and 3 hours for the second visit.

For peer review only - http://bmjopen.bmj.com/site/about/guidelines.xhtml

- Taking a chest X-ray to test for TB. There will be one other test for TB which will either be a blood test or a Mantoux test (a skin reaction test), depending on what your hospital's local procedures are.
- Taking a urine sample to test for infection, kidney function and a routine pregnancy test for all girls.

If one of these tests tells us that it is not safe for you to take part, we will let you know straight away. If the tests are OK, then we will proceed with a second screening visit for a few more tests.

Screening visit 2:

- Doing a general health check.
- Asking about any medicine you are taking and any illnesses you have had or still have since your last visit.
- Taking a urine sample to test for infection, kidney function and a routine pregnancy test for all girls.
- You and your parent/carer will be asked to complete a short questionnaire about your diabetes and general health.
- You will be given a free blood glucose monitor (FreeStyle Libre) and sensors to use for the study. The sensor should be worn two weeks prior to every study visit but can be worn constantly if you find it helpful.
- A Mixed Meal Tolerance Test (or Milkshake test). This test tells us how much insulin your body is still making. During this test we also take additional blood samples to test the immune system and pancreas function.

On the day of the Mixed Meal Tolerance Test, you will need to make sure that you have not eaten or drunk anything except water from midnight the night before onwards. You will also be asked to not take your early morning short acting insulin because you will not be eating breakfast. You will need to tell us your blood glucose levels on waking so that we can make sure it's OK for you to be tested. It will need to be between 4.0 and 11.1mmol/L for the screening visit to happen. If it is lower, the test will be rescheduled but if it is higher, you may be advised to take short acting insulin so that the visit can go ahead.

.

At the hospital, you will have blood taken through a small plastic tube (cannula) which we will insert in your arm (using local anaesthetic cream/spray if you want it). This will stay in your arm during the test so that we can take blood samples more easily.

Then you will be given a milkshake to drink (various flavours available). The research doctor or nurse will take blood at fixed times over the next 2 hours. Over this time, less than 1 tablespoon (10ml) of blood will be taken from you in total. During this time, you can relax on a bed, play games, read or study. Once the test is completed we will give you something to eat and drink and you will receive insulin in whatever dose is needed.

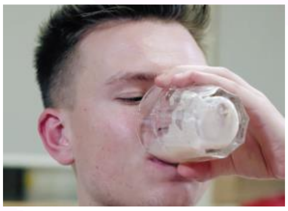

An additional 40mls (less than 3 tablespoons) will be taken at the same visit for testing in our laboratories.

Your blood samples will be analysed within 2 weeks and if the test shows that you are still making some of your own insulin, you will be contacted by the research team to tell you that everything is OK to be part of the study and to arrange the first injection.

If it is not convenient to have two separate visits for testing, we can arrange to combine the two sets of tests if you let us know beforehand. This is because the combined screening visit needs you to be fasted on arrival.

Study Visit 1

If you take part in this study you will be randomly allocated to either the study medicine group or the placebo (a "dummy" medicine that has no effect) group. This is decided by chance using a computer programme before the study visit and neither you nor the research team will know until the end of the study what treatment you have received.

2 out of every 3 people taking part will receive the study medicine compared with only 1 out of 3 getting the placebo. This is to give people a better chance of getting the study medicine.

Your local hospital or research centre.

Approximately 2 hours.

For peer review only - http://bmjopen.bmj.com/site/about/guidelines.xhtml

	Treatment visits will be booked in so that you receive your injection at the required intervals. The second dose will be four weeks after the first. All other doses afterwards will be eight weeks apart. These will be booked in advance so that any issues with attendance can be identified as soon as possible (e.g. holidays, exams). Postponing a treatment visit may result in the treatment being stopped if too much time has passed because the levels of the study medicine in your body may go too low and won't work anymore.<br>You will have a physical examination and tests will be done on your urine and blood samples (up to 57.5ml which is about 3 and a half tablespoons) at this visit.<br>Then you will receive an injection of either the study medicine, Ustekinumab, or the placebo. Injections are given under the skin using a very small needle similar to the one that you already use for daily insulin injections.<br>You will be asked to stay in the hospital for 1 hour after you receive the injection so that the study team can make sure that there are no side effects and that you are safe to leave.	
**Study Visit 2**<br>*4 weeks after the 1st visit*	You will have a physical examination and be asked questions about your health. We will also need a urine sample and a blood sample (up to 50.5ml which is just under 3 tablespoons). Then you will receive an injection of either the study medicine, Ustekinumab or placebo, depending on which treatment group you are in.<br>We will also download the data stored on your blood glucose monitor.	Your local hospital or research centre.<br>Approximately 1 hour
**Study Visit 3**<br>*12 weeks into the study*	You will have a physical exam and be asked questions about your health. We will also need a urine sample and a blood sample (up to 59.5ml which is nearly 3 and a half tablespoons).<br>Next you will receive an injection of either the study medicine, Ustekinumab or placebo, depending on which treatment group you are in.<br>We will also download the data stored on your blood glucose monitor.	Your local hospital or research centre.<br>Approximately 1 hour
**Study Visit 4**<br>*20 weeks into the study*	At this visit you will receive an injection of either the study medicine or placebo. You will also have a urine test but no blood sample will be needed.<br>This appointment can be done at your home.	Your local hospital or research centre or your home.<br>Approximately 1 hour
**Study Visit 5**<br>*28 weeks into the study*	You will be asked to do a second milkshake test in exactly the same way as described earlier and will involve taking half a tablespoon (10ml) of blood over a 2 hour period. This means that you have to arrive fasted for this study visit.<br>You will also have a physical examination, and be asked questions about your health. We will also need a urine sample and another blood sample (up to 61.5ml which is 3 and a half tablespoons).<br>Then you will receive an injection of either the study medicine, Ustekinumab or placebo, depending on which treatment group you are in.<br>This visit will also include both you and your parent/carer completing a short questionnaire exactly like the one you did at the screening visit.<br>We will also download the data stored on your blood glucose monitor.	Your local hospital or research centre.<br>Approximately 3 hours
**Study Visit 6**<br>36 weeks into the study	At this visit you will receive an injection of either the study medicine or placebo. You will also have a urine test but no blood sample will be needed.<br>This appointment can be done at your home.	Your local hospital or research centre or your home.<br>Approximately 1 hour
**Study Visit 7**<br>44 weeks into the study	At this visit you will receive the FINAL injection of either the study medicine or placebo. You will also have a urine test but no blood sample will be needed.<br>This appointment can be done at your home.<br>We will also download the data stored on your blood glucose monitor.	Your local hospital or research centre or your home.<br>Approximately 1 hour
**Study Visit 8 – follow up**	At this final visit you will have a physical examination and be asked questions about your health. You will be asked to do a final milkshake test in exactly the same way as described earlier and will involve taking half a tablespoon (10ml) of blood over a 2 hour period.	Your local hospital or research centre.

For peer review only - http://bmjopen.bmj.com/site/about/guidelines.xhtml

52 weeks into the study	We will also need a urine sample and another blood sample (up to 61.5ml which is 3 and a half tablespoons).<br>This visit will also include both you and your parent/carer completing a short questionnaire exactly like the one you did at the screening visit.<br>We will also download the data stored on your blood glucose monitor.	Approximately 3 hours
**Remote follow up**<br>Weeks 78 & 104	We will check your hospital records to find out how you are doing. You do not need to come into the hospital for a visit. We may need to call you up at home to check that you are OK and in good health.	No visit needed

WHAT ELSE WILL I BE ASKED TO DO?

As well as coming to your local hospital or research centre for study visits there are a few other things we will ask you to do during the study:

- You will be asked to complete a diary between study visits to record:
    a) How much insulin you take during the study (for the two weeks before every study visit)
    b) If you feel or have been unwell or have to take any other medicines during the study.
    c) If you have any hypoglycaemic (low blood glucose levels) episodes that need treating.

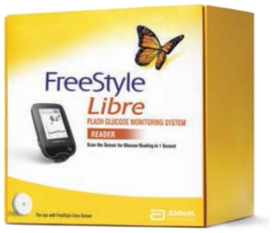

- We will ask you to test your blood glucose levels at home for at least 2 weeks before each visit using our FREE Abbott Freestyle Libre flash glucose monitoring system so you don't need to do extra finger prick tests for the trial. You will need to wear a sensor on your arm for the two weeks before our study visit if you want to be in the trial. We will show you how it works. You are free to use the monitor at home for the rest of the time of study if you want to. We will give you enough sensors to allow this for a year.

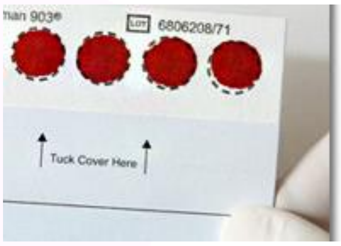

- We will ask you to give blood spot samples which you can do at home. You will need to do this once a week for 28 weeks, then every month for the next 6 months. The test involves pricking your finger like a normal finger prick blood glucose test and dabbing the blood spot onto a special card. You will need to do this before the first meal of the day and then 1 hour later. The card should be posted to our special laboratory for testing. We will show you how to do this and will provide envelopes and pay for the postage.
- You will be asked to make sure that you do not have certain vaccinations before, during and immediately after the study. If you need a vaccination, for example if you are travelling abroad, you must tell the study doctor or nurse immediately.
- Please be aware that a urine pregnancy test will be done for all females at each study visit. We need to do this because the law requires us to do this in clinical trials because the effects of the study medicine are not known in pregnancy and we would want the mother and baby to be safe. Any confirmed pregnancies will be monitored closely with your permission (including the female partners of male participants).
- Rather than asking if you are engaged in actions that may lead to pregnancy, we will ask everybody to agree to use adequate contraception (hormonal based contraception, barrier contraception, abstinence) until 4 months following the date of their final treatment. All participants will need to agree to this to take part. Your GP or a pharmacist can advise on suitable contraception if you don't want to discuss this with your parents/carers.
- Finally, we would like you to complete a short questionnaire about your health and diabetes. These questionnaires will be done at the second screening visit and study visits 5 and 8. Your parent/carer will also be asked to complete questionnaires at these time points so we will ask that they agree to attend those study visits with you.

WILL THE STUDY HELP ME?

If you have been allocated to the group that receives the study medicine, Ustekinumab, it is possible that it will help your pancreas make insulin for longer. However, we cannot say this for certain until we have completed this study.

For peer review only - http://bmjopen.bmj.com/site/about/guidelines.xhtml

During the study your diabetes will be very closely monitored. This will include regular check-ups with your local diabetes team including routine blood testing. You will have more time with the research team to discuss your diabetes and ask questions than at a normal clinic appointment.

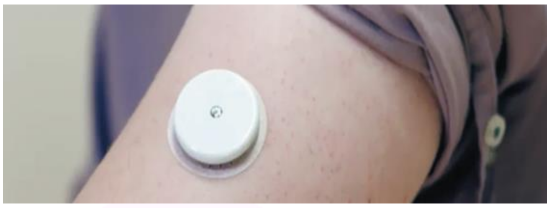

You will be provided with a FREE Abbott Freestyle Libre flash glucose monitoring system. You can use this to check your blood sugar levels while you are in the study, although you will still need to do some finger prick tests. The treatments will stop at week 44 and you will not receive any further injections of the study drug / placebo during the trial.

WHAT HAPPENS WHEN THE STUDY STOPS?

We will collect all the information together and we will decide if the study medicine can help people with Type 1 Diabetes make their own insulin for longer. If it does then we will carry out a bigger version of this study.

You will be informed which treatment you were given and your medical records will be updated with the treatment information.

WHAT IF NEW INFORMATION COMES ALONG?

Sometimes during research, we get new information about the treatment being studied. If this happens, we will tell you about it and discuss whether you want to continue in the study.

WHAT IF I DO NOT WANT TO TAKE PART ANYMORE?

Just let your study doctor or research nurse know about your decision. You will be asked whether you wish to withdraw from just having the study treatment or from the whole study (including the study visits and data collection).

You can withdraw from treatment but still come to study visits for sample and data collection. If you want to withdraw completely, we will make a note of this and we will make sure that you are transferred back to normal care as quickly as possible.

WHAT IF THERE IS A PROBLEM OR SOMETHING GOES WRONG?

If you feel unwell or suffer any unusual discomfort during the study it is important to inform the study doctor or nurse as soon as possible. If it is because of something in the study, we need to consider stopping your treatment. The diabetes care team will also be informed.

If you feel overwhelmed by your recent diagnosis, you can call the local [title] on [tel number / email] and they can talk through your concerns with you.

If you are unhappy about the conduct of the study and wish to complain, you can do this through:

(name and contact details of appropriate organisations – site specific).

WHAT ARE THE POSSIBLE SIDE EFFECTS FOR ME IN PARTICIPATING IN THE STUDY?

You may get a bruise or a little discomfort at the site of the blood tests.

The FreeStyle Libre sensor may cause a slight rash for some people who might be allergic to the adhesive on the sensor. The manufacturer are always improving their sensors to stop this happening but you may experience some discomfort from wearing the sensor. Please let the research team know if this happens.

During the Milkshake test, you may experience changes in blood glucose level because you will not have taken insulin immediately beforehand. The study nurses and doctors will be available to help you make any changes to your usual insulin doses after this test.

The medicine (Ustekinumab) being used in this study is currently used in patients with skin and bowel conditions safely. Because the medicine acts on the immune system, there is a possibility that it will increase the risk of infections and cancer, but so far this has not been found to be a problem with people treated with this medicine for others diseases. It is also possible that you may get an allergic reaction to the treatment injection. We will ask you to stay for one hour after your first injection to check for any reactions.

It is routine to check people who receive this medicine for tuberculosis (TB) as if you have this infection the study medicine may make it worse. If there is evidence of TB infection, you will not be allowed to take part.

If you take part in this study, you will have a chest X-ray to rule out TB which is additional to standard care. Chest x-rays involve using ionising radiation to form images of the body. Ionising radiation can cause cell damage in the

For peer review only - http://bmjopen.bmj.com/site/about/guidelines.xhtml

longer term which can sometimes lead to cancer developing. However, we only ask for one x-ray so taking part in this study will add only a very small chance of this happening to you when you are older. The risk is not much greater than that found with natural background radiation.

If you feel ill at any time during the trial and go to your GP or the hospital, please show them the membership card you will be given so that they can contact the research team to ask about possible side effects.

The research team will carefully monitor you throughout the study to check your health and to ensure that you are not experiencing any side effects. You must tell someone straight away if you feel unwell.

WILL I RECEIVE ANY PAYMENT FOR TAKING PART?

You will receive a £10.00 gift voucher for each treatment visit and we will give you £30.00 gift if you come to the final visit (visit 8) (that's £100 in total if you come to all visits).

You will be able to claim back your travel expenses for getting to the local hospital or research centre for all screening, treatment and follow up visits.

WHAT INFORMATION WILL YOU COLLECT AND HOW WILL IT BE KEPT PRIVATE?

We will ask for your name and contact details so that the research nurse can keep in touch and manage your visits. We will also need to collect data about your health that we get from your tests, your flash glucose monitor and your medical records. We will also ask to complete a questionnaire at three time points.

The people in our research team at the local hospital or research centre will know that you are taking part. The doctors looking after you when you come to hospital will also know that you are in the study. Your medical notes may be looked at by staff from Swansea and Cardiff Universities or NHS and regulatory auditors who will be checking that the study is being done correctly. If you agree, we will also tell your family doctor (GP) that you are in the study.

People at the research laboratories will not know who you are when they test your samples. You will be given a study number to replace your name so any study samples and data related to you will be anonymised. The questionnaires and the data from your flash glucose monitor will also use a study number instead of your name.

All information collected about you during the study will be kept by the research nurse in a locked cabinet and entered onto a secure database. Only people with the password can open up the database.

Cardiff University is the sponsor for this study based in the United Kingdom. Cardiff University will be using information from you and your medical records in order to undertake this study and will act as the data controller for this study. This means that we are responsible for looking after your information and using it properly. Cardiff University will keep identifiable information about you for 25 years after the study has finished.

Your rights to access, change or move your information are limited, as we need to manage your information in specific ways in order for the research to be reliable and accurate. If you withdraw from the study, we will keep the information about you that we have already obtained. To safeguard your rights, we will use the minimum personally-identifiable information possible.

You can find out more about how we use your information by contacting inforequest@cardiff.ac.uk.

WHAT WILL HAPPEN TO ANY SAMPLES I GIVE?

We want to test your blood and urine to better understand your diabetes and how the study medicine or placebo affects your diabetes. The blood samples will be used to test:

1. The amount of insulin your body still makes before and after taking part in the study.
2. Your average blood glucose levels
3. The antibodies to the insulin making cells
4. General health checks – anaemia, kidneys, liver etc
5. How much of the study medication is in your blood.
6. How your body's immune system is reacting to the study medication.

Scientists in laboratories around the UK will look at your anonymised blood samples. These samples will be stored in a safe place. Some of your blood samples will be sent to a laboratory in Europe and either America or Canada for special testing to find out how much of the study medicine is present.

We would like to keep any leftover blood samples in a special tissue repository permanently - we will ask for your permission to do this. The samples will only be accessed by scientists who have special permission to do so. The samples might be sent outside the UK to other research teams in Europe or countries such as America and Canada if you agree to this. These other teams must have permission from us to use your sample before we send it. If you

For peer review only - http://bmjopen.bmj.com/site/about/guidelines.xhtml

do not want to agree to this, your samples will be destroyed after they have been analysed.

WILL ANY GENETIC TESTS BE DONE?

We will use your blood samples to help us study the genes involved with diabetes and the immune system. These samples will not have your name on them and will not be used for any other reason without your permission.

WHAT WILL HAPPEN TO THE RESULTS OF THIS STUDY?

The full results of this study will not be known until the last patient has completed their tests, which may take more than 5 years. The research results will be reported in scientific publications and meetings but you will not be identified by name at all. If you are interested in receiving a summary of the research results, we can arrange this.

WHO IS ORGANISING AND FUNDING THE STUDY?

The study is being organised by researchers at Cardiff University and Swansea University.

It is being funded by a grant from the National Institute for Health and Research (NIHR).

WHERE IS THE STUDY BEING DONE?

The study is being done at hospitals and research centres across England, Wales and Scotland.

WHO HAS CHECKED THIS STUDY?

Before any research goes ahead it has to be checked by a Research Ethics Committee. This is a group of people who make sure that the research is OK to do and to make sure that the patient will be safe. This study has been looked at by Wales REC 3. As this study is looking at a medicine, it has also been approved by the government's Medicine and Healthcare products Regulatory Authority (MHRA) who check that the researchers carry out the study safely. It has also been checked by national and local NHS organisations to make sure that the study can be done using their site and staff.

WHAT SHOULD I DO NOW?

If you are interested in taking part, or have any questions please contact one of the following people:

**Name:**	**Name:**	**Name:** (only if needed)
**Role:** Principal Investigator	**Role:** Research nurse	**Role:**
**Tel. No:**	**Tel. No:**	**Tel. No:**
**Email:**	**Email:**	**Email:**

Alternatively, you may want to speak to someone at the USTEKID Trial Office who are managing the study, based at Swansea University. The Trial Manager's details are below:

Name: Dr Kym Thorne
Tel. No: 01792 606372 (direct) or 01792 606545 for Swansea Trials Unit
Email: ustekid@swansea.ac.uk
Address: Floor 2, Institute of Life Sciences 2, Swansea University Medical School, Singleton Park, Swansea SA2 8PP

Thank you for taking the time to read this information sheet and for considering taking part in this research study

For peer review only - http://bmjopen.bmj.com/site/about/guidelines.xhtml

[Insert local headers]

USTEKID

NHS National Institute for Health Research

CARDIFF UNIVERSITY PRIFYSGOL CAERDYD

Swansea University Prifysgol Abertawe Swansea Trials Unit Uned Dreialon Abertawe

A research study to see if the medicine Ustekinumab can make diabetes easier to manage

CONTACT DETAILS FOR STUDY TEAM:

NURSE:

DOCTOR:

EMERGENCIES:

FOR PARENTS OF YOUNG PEOPLE AGED 12-15 YEARS OLD

We would like you to help us with our research study. Please read this information carefully and talk to your parent or carer about the study. Ask us if there is anything that is not clear or if you want to know more. Take time to decide if you want to take part. It is up to you if you want to do this. If you do not, then that is fine, you will be looked after by your doctors just the same.

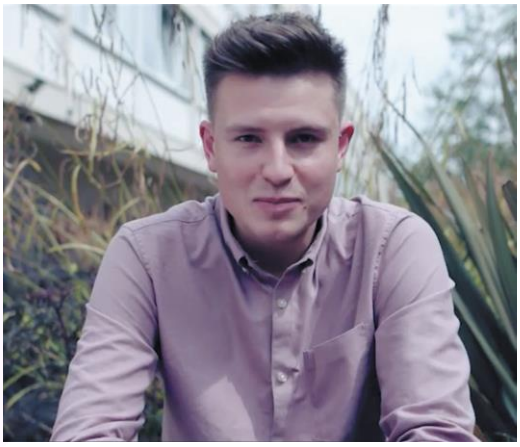

Please look at our video explaining the trial at www.type1diabetesresearch.org.uk/current-trials. The blue box below and the video contains the key points about the study. If you would like to know more, please read the rest of this leaflet.

KEY POINTS ABOUT THE STUDY:

USTEKID

- We want to see if the study medicine, **Ustekinumab**, can make Type 1 Diabetes (the type your child has) easier to manage. The medicine works by "protecting" some of the cells in the pancreas that still produce insulin from attack by the immune system.
- The study will involve your child having an injection every 1-3 months with either the study medicine, Ustekinumab or a placebo (a "dummy medicine"). These injections are given under the skin just like insulin injections and will be done by the study doctor or nurse. Neither you nor the research team will know if your child receives the study medicine or the placebo.
- The medicine is already being used to treat other illnesses quite safely. We will give you information about possible side effects before you decide if your child will take part.
- You will be asked to bring your child to your local hospital or research centre for 10 study visits over a 15 month period, but where possible this will be on the same day as your routine hospital visits. The first two will check if you are eligible to take part. Three of these visits can sometimes be done by a research nurse at home.
- We will ask your child to provide extra blood and urine samples to check that it is safe for them to take part in the study and to check their health, their blood glucose levels and how their immune system is working during the study.
- At three visits, they will have blood tests over a 2 hour period to see how much insulin their body is making.
- We will provide them with a Flash Glucose Monitor (Freestyle Libre) to wear for 2 weeks before each visit. They can keep the monitor for use at home for the whole time of the study.
- Your child will be offered a small gift voucher for each visit and your travel expenses will be paid.
- Your child can stop taking part in the study at any time and they do not have to give a reason why.

For peer review only - http://bmjopen.bmj.com/site/about/guidelines.xhtml

WHY IS THIS STUDY BEING DONE?

This study is being done to see if a medicine called **Ustekinumab** can help to "protect" the cells in the body that produce insulin in young people recently diagnosed with Type 1 Diabetes. It is caused by the body's own immune system damaging the cells in the pancreas that make insulin. Our aim is to develop a treatment that can slow this process by targeting the immune cells causing the damage.

At the time of diagnosis, most children have 10-20% of their insulin-producing cells still working. It usually takes between 1 and 5 years before they stop working completely. Sometimes these last few working cells can make enough insulin to make blood glucose levels stable and easier to control – this is called the "Honeymoon period". This period is only temporary and doesn't last. Ustekinumab, the study medicine, may make this period last longer by reducing the damaging effects of the immune system on the remaining insulin-producing cells in the pancreas.

Ustekinumab is currently given to adults and teenagers with particular skin and bowel problems and it is known to be safe to use and effective at treating those conditions.

WHY HAS MY CHILD BEEN ASKED TO TAKE PART?

Your child has been chosen because they are aged 12 - 18 years old and have recently been diagnosed with Type 1 diabetes.

DOES MY CHILD HAVE TO TAKE PART?

No. It is completely up to both your child and you whether or not they should take part and you or your child can always change your minds at any time. If you and your child are interested in this study then:

- Let us know by calling one of the people listed at the end of this information sheet.
- A member of the research team will contact you to explain more about the study and answer any questions you have.
- If your child wants to take part, they will be asked to agree to sign a form.
- As your child is under 16 years of age, you will need to sign a consent form to say that you agree for your child to take part. The consent form will also ask you to agree to complete three short questionnaires about your child's health. For this reason, it is preferable if the person most likely to attend the study visits with the child is the person who signs the consent form.
- You will be given this information sheet and a copy of your signed consent form to keep.

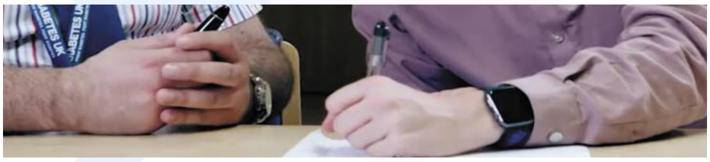

If you make a decision to allow your child to take part, ***you are still free to withdraw them from the study at any time without giving a reason***. This will not affect the medical care they get from their diabetes doctor in any way.

You do not need to decide if you want to allow your child to take part straight away. You can take your time and talk about the study with your child, family, friends and the study team if you want to. You need to let us know within 6 weeks of your child being diagnosed with diabetes, so that we can start the treatment early enough.

WHAT HAPPENS ONCE MY CHILD HAS AGREED TO TAKE PART?

VISIT	WHAT WILL HAPPEN	WHERE AND HOW LONG WILL IT TAKE?
Screening visits	Before we can start the treatment, we need to check that your child is eligible to take part in the study - this is called "screening". You will be asked to bring your child to your local hospital or research centre to talk about the study. This is where you will be able to ask questions.<br><br>If you are happy for your child to take part you will both be asked to sign consent forms before we begin the screening tests which involve:<br><br>Screening visit 1:	Your local hospital or research centre.<br><br>Approximately 1 hour for the first visit and 3 hours

For peer review only - http://bmjopen.bmj.com/site/about/guidelines.xhtml

for the second visit.

- Doing a general health check (this includes a general examination and measuring their height, weight and blood pressure).
- Asking about any medicine they are taking and any illnesses they have had or still have.
- Taking some blood samples (between 0.5 - 2 tablespoons) from their arm to check their general health and diabetes, as well as testing for infections such as TB (tuberculosis), hepatitis and HIV. Blood volumes vary according to your hospital's local testing procedures.
- Taking a chest X-ray to test for TB. There will be one other test for TB which will either be a blood test or a Mantoux test (a skin reaction test), depending on what your hospital's local procedures are.
- Taking a urine sample to test for infection, kidney function and a routine pregnancy test for all girls.

If one of these tests tells us that it is not safe for your child to take part, we will let you know straight away. If the tests are OK, then we will proceed with a second screening visit for a few more tests.

Screening visit 2:

- Doing a general health check.
- Asking about any medicine they are taking and any illnesses they have had or still have since their last visit.
- Taking a urine sample to test for infection, kidney function and a routine pregnancy test for all girls.
- You and your child will be asked to complete a short questionnaire about their diabetes and general health.
- Your child will be given a free blood glucose monitor (FreeStyle Libre) and sensors to use for the study. The sensor should be worn two weeks prior to every study visit but can be worn constantly if your child finds it helpful.
- A Mixed Meal Tolerance Test (or Milkshake test). This test tells us how much insulin their body is still making. During this test we also take additional blood samples to test the immune system and pancreas function.

On the day of the Mixed Meal Tolerance Test, you will need to make sure that your child has not eaten or drunk except water anything from midnight the night before onwards. They will also be asked to not take their early morning short acting insulin because they will not be eating breakfast. You will need to tell us your child's blood glucose levels on waking so that we can make sure it's OK for them to be tested. They will need to be between 4.0 and 11.1mmol/L for the screening visit to happen. If their blood glucose level runs low before coming to the hospital and needs to be treated, the test will be rescheduled but if they are higher, your child may be advised to take short acting insulin so that the visit can go ahead.

At the hospital, your child will have blood taken through a small plastic tube (cannula) which we will insert in their arm (using local anaesthetic cream/spray if they want it). This will stay in their arm during the test so that we can take blood samples more easily.

Then they will be given a milkshake to drink (various flavours available). The research doctor or nurse will take blood at fixed times over the next 2 hours. Over this time, less than 1 tablespoon (10ml) of blood will be taken from them in total. During this time, they can relax on a bed, play games, read or study. Once the test is completed we will give them something to eat and drink and they will receive insulin in whatever dose is needed.

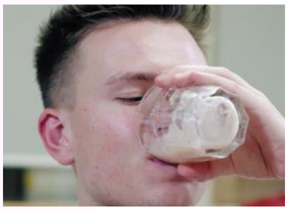

An additional 40mls (less than 3 tablespoons) will be taken at the same visit for testing in our laboratories.

The blood samples will be analysed within 2 weeks and if the test shows that they are still making some of their own insulin, you will be contacted by the research team to tell you that everything is OK for your child to be part of the study and to arrange the first injection.

For peer review only - http://bmjopen.bmj.com/site/about/guidelines.xhtml

	If it is not convenient to have two separate visits for testing, we can arrange to combine the two sets of tests if you let us know beforehand. This is because the combined screening visit needs your child to be fasted on arrival.	
**Study Visit 1**	If your child is eligible to take part in this study, they will be randomly allocated to either the study medicine group or the placebo (a "dummy" medicine that has no effect) group. This is decided by chance using a computer programme before the study visit and neither you nor the research team will know until the end of the study what treatment they received.<br><br>2 out of every 3 people taking part will receive the study medicine compared with only 1 out of 3 getting the placebo. This is to give people a better chance of getting the study medicine.<br><br>Treatment visits will be booked in so that your child will receive injections at the required intervals. The second dose will be four weeks after the first. All other doses afterwards will be eight weeks apart. These will be booked in advance so that any issues with attendance can be identified as soon as possible (e.g. holidays, exams). Postponing a treatment visit may result in the treatment being stopped if too much time has passed because the levels of the study medicine in your child's body may go too low and won't work anymore.Your child will have a physical examination and tests will be done on their urine and blood samples (57.5ml which is about 3 and a half tablespoons) at this visit.<br><br>Then they will receive an injection of either the study medicine, Ustekinumab, or the placebo. Injections are given under the skin using a very small needle similar to the one that they already use for daily insulin injections.<br><br>They will be asked to stay in the hospital for 1 hour after they receive the injection so that the study team can make sure that there are no side effects and that your child are safe to leave.	Your local hospital or research centre.<br><br>Approximately 2 hours.
**Study Visit 2**<br><br>*4 weeks after the 1st visit*	Your child will have a physical examination and be asked questions about your health. We will also need a urine sample and a blood sample (up to 50.5ml which is just under 3 tablespoons). Then your child will receive an injection of either the study medicine, Ustekinumab or placebo, depending on which treatment group they are in.<br><br>We will also download the data stored on their blood glucose monitor.	Your local hospital or research centre.<br><br>Approximately 1 hour
**Study Visit 3**<br><br>*12 weeks into the study*	Your child will have a physical exam and be asked questions about your health. We will also need a urine sample and a blood sample (up to 59.5ml which is nearly 3 and a half tablespoons).<br><br>Next your child will receive an injection of either the study medicine, Ustekinumab or placebo, depending on which treatment group they are in.<br><br>We will also download the data stored on their blood glucose monitor.	Your local hospital or research centre.<br><br>Approximately 1 hour
**Study Visit 4**<br><br>*20 weeks into the study*	At this visit your child will receive an injection of either the study medicine or placebo. They will also have a urine test but no blood sample will be needed.<br><br>This appointment may be done at your home.	Your local hospital or research centre or your home.<br><br>Approximately 1 hour
**Study Visit 5**<br><br>*28 weeks into the study*	Your child will be asked to do a second milkshake test in exactly the same way as described earlier and will involve taking less than 1 tablespoon (10ml) of blood over a 2 hour period. This means that they have to arrive fasted for this study visit.<br><br>Your child will also have a physical examination, and be asked questions about their health. We will also need a urine sample and another blood sample (up to 61.5ml which is 3 and a half tablespoons).<br><br>Then your child will receive an injection of either the study medicine, Ustekinumab or placebo, depending on which treatment group they are in.<br><br>This visit will also include both you and your child completing a short questionnaire exactly like the one you did at the screening visit.<br><br>We will also download the data stored on their blood glucose monitor.	Your local hospital or research centre.<br><br>Approximately 3 hours

For peer review only - http://bmjopen.bmj.com/site/about/guidelines.xhtml

**Study Visit 6**<br>36 weeks into the study	At this visit your child will receive an injection of either the study medicine or placebo. They will also have a urine test but no blood sample will be needed.<br>This appointment may be done at your home.	Your local hospital or research centre or your home.<br>Approximately 1 hour
**Study Visit 7**<br>44 weeks into the study	At this visit your child will receive the <u>FINAL</u> injection of either the study medicine or placebo. They will also have a urine test but no blood sample will be needed.<br>This appointment may be done at your home.<br>We will also download the data stored on their blood glucose monitor.	Your local hospital or research centre or your home.<br>Approximately 1 hour
**Study Visit 8 – follow up**<br>52 weeks into the study	At this final visit your child will have a physical examination and be asked questions about their health. They will be asked to do a final milkshake test in exactly the same way as described earlier and will involve taking half a tablespoon (10ml) of blood over a 2 hour period.<br>We will also need a urine sample and another blood sample (up to 61.5ml which is 3 and a half tablespoons).<br>This visit will also include both you and your child completing a short questionnaire exactly like the one you did at the screening visit.<br>We will also download the data stored on their blood glucose monitor.	Your local hospital or research centre.<br>Approximately 3 hours
**Remote follow up**<br>Weeks 78 & 104	We will check your child's hospital records to find out how they are doing. They do not need to come into the hospital for a visit. We may need to call you up at home to check that they are OK and in good health.	No visit needed

WHAT ELSE WILL MY CHILD AND I BE ASKED TO DO?

As well as attending with your child to your local hospital or research centre for study visits there are a few other things we will ask your child to do during the study which may involve your help:

- Your child will be asked to complete a diary between study visits to record:
    a) How much insulin they take during the study (for the <u>two weeks before every study visit</u>).
    b) If they feel or have been unwell or have to take any other medicines during the study.
    c) If they have any hypoglycaemic (low blood glucose levels) episodes that need treating.

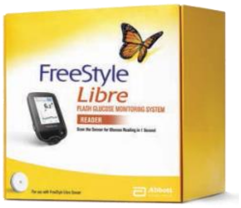

- We will ask them to test their blood glucose levels at home for at least 2 weeks before each visit using our FREE Abbott Freestyle Libre glucose monitoring system so they don't need to do extra finger prick tests for the trial. They will need to wear a sensor on their arm for the two weeks before our study visit if they want to be in the trial. We will show you both how it works. They are free to use the monitor at home for the rest of the time of study if they want to. We will give them enough sensors to allow this for a year.

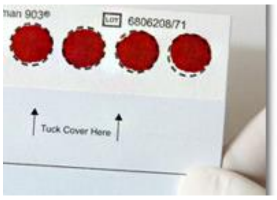

- We will ask them to give a blood spot sample which they can do at home. They will need to do this once a week for 28 weekly, then every month for the next 6 months. The test involves pricking their finger like a normal finger prick blood glucose test and dabbing the blood spot into a special card. They will need to do this before the first meal of the day and then 1 hour later. The paper should be posted to a special laboratory for testing. We will show you both how to do this and will provide envelopes and pay for the postage.

You will be asked to make sure that your child does not have certain vaccinations before, during and immediately after the study. If they need a vaccination, for example if they are travelling abroad, you must tell the study doctor or nurse immediately.

Please be aware that a urine pregnancy test will be done for all females at each study visit. We need to do this

For peer review only - http://bmjopen.bmj.com/site/about/guidelines.xhtml

because the law requires us to do this in clinical trials because the effects of the study medicine are not known in pregnancy and we want the mother and baby to be safe. Any confirmed pregnancies will be monitored closely with your permission (including the female partners of male participants). Rather than asking if your child is engaged in actions that may lead to pregnancy, we will ask everybody (males and females) to agree to use adequate contraception (hormonal based contraception, barrier contraception, abstinence) until 4 months following the date of their final treatment. You will need to agree to this on behalf of your child if you want them to take part. Your GP or a pharmacist can advise on suitable contraception for your child.

Finally, we would like you to complete a short questionnaire about your child's health and diabetes. These questionnaires will be done at the second screening visit and study visits 5 and 8. It is important that the person who consents will complete all three questionnaires.

WILL THE STUDY HELP MY CHILD?

If your child has been allocated to the group that receives the study medicine, Ustekinumab, it is possible that it will help their pancreas make insulin for longer. However we cannot say this for certain until we have completed this study. During the study your child's diabetes will be very closely monitored. This will include regular check-ups with your local diabetes team including routine blood testing. You and your child will have more time with the research team to discuss their diabetes and ask questions than at a normal clinic appointment.

Your child will be provided with a FREE Abbott Freestyle Libre blood glucose monitoring system. They can use this to check their blood glucose levels while they are in the study, although they will still need to do some finger prick tests. The treatments will stop at week 44 and your child will not receive any further injections of the study drug / placebo during the trial.

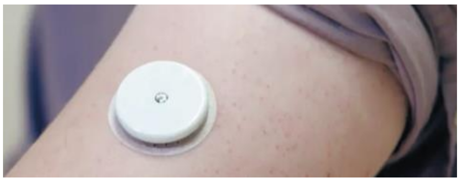

WHAT HAPPENS WHEN THE STUDY STOPS?

We will collect all the information together and we will decide if the study medicine can help people with Type 1 Diabetes make their own insulin for longer. If it does then we will carry out a bigger version of this study. You and your child will be informed which treatment they were given and their medical records will be updated with the treatment information.

WHAT IF NEW INFORMATION COMES ALONG?

Sometimes during research, we get new information about the treatment being studied. If this happens, we will tell you about it and discuss whether you and your child want to continue in the study.

WHAT IF I MY CHILD DOES NOT WANT TO TAKE PART ANYMORE?

Just let your study doctor or research nurse know about your decision. You and your child will be asked whether you wish to withdraw from just having the study treatment or from the whole study (including the study visits and data collection). You can withdraw from treatment but still come to study visits for sample and data collection. If they want to withdraw completely, we will make a note of this and we will make sure that your child are transferred back to normal care as quickly as possible.

WHAT IF THERE IS A PROBLEM OR SOMETHING GOES WRONG?

If your child feels unwell or suffers any unusual discomfort during the study it is important to inform the study doctor or nurse as soon as possible. If it is because of something in the study, we need to consider stopping your child's treatment.

If you or your child feel overwhelmed by their recent diagnosis, you can call the local [title] on [tel number / email] and they can talk through any concerns with you both.

If you are unhappy about the conduct of the study and wish to complain, you can do this through:

(name and contact details of appropriate organisations – site specific).

WHAT ARE THE POSSIBLE SIDE EFFECTS FOR MY CHILD IN PARTICIPATING IN THE STUDY?

They may get a bruise or a little discomfort at the site of the blood tests.

The FreeStyle Libre sensor may cause a slight rash for some people who might be allergic to the adhesive on the

For peer review only - http://bmjopen.bmj.com/site/about/guidelines.xhtml

sensor. The manufacturer are always improving their sensors to stop this happening but your child may experience some discomfort from wearing the sensor. Please let the research team know if this happens.

During the Milkshake test, they may experience changes in blood glucose level because they will not have taken insulin immediately beforehand. The study nurses and doctors will be available to help your child make any changes to their usual insulin doses after this test.

The medicine (Ustekinumab) being used in this study is currently used in patients with skin and bowel conditions. Because the medicine acts on the immune system, there is a possibility that it will increase the risk of infections and cancer, but so far, this has not been found to be a problem with people treated with this medicine for others diseases. It is also possible that they may get an allergic reaction to the treatment injection. We will ask them to stay for one hour after their first injection to check for any reactions.

It is routine to check people who receive this medicine for tuberculosis (TB) as, if your child have this infection, the study medicine may make it worse. We will check your child and if there is evidence of TB infection, they will not be allowed to take part.

If your child takes part in this study, they will have a chest X-ray to rule out TB which is additional to standard care. Chest x-rays involve using ionising radiation to form images of the body. Ionising radiation can cause cell damage in the longer term which can sometimes lead to cancer developing. However, we only ask for one x-ray so taking part in this study will add only a very small chance of this happening to them when they are older. The risk is not much greater than that found with natural background radiation.

If your child feels ill at any time during the trial and you go to your GP or the hospital, please show them the membership card your child will be given so that they can contact the research team to ask about possible side effects.

The research team will carefully monitor your child throughout the study to check their health and to ensure that they are not experiencing any side effects. You must tell someone straight away if they complain that they feel unwell.

WILL MY CHILD RECEIVE ANY PAYMENT FOR TAKING PART?

Your child will receive a £10.00 gift voucher for each treatment visit and we will give them £30.00 gift if they come to the final visit (visit 8) (that's £100 in total if they come to all visits).

You will be able to claim back your travel expenses for getting your child to the local hospital or research centre for all screening, treatment and follow up visits.

WHAT INFORMATION WILL YOU COLLECT AND HOW WILL IT BE KEPT PRIVATE?

We will ask for your name and contact details as well as your child's name so that the research nurse can keep in touch and manage your visits. We will also need to collect data about your child's health which we get from their tests, their blood glucose monitor and their medical records. We will also ask you and your child to complete a questionnaire each at three time points.

The people in our research team at the local hospital or research centre will know that your child is taking part. The doctors looking after your child when you come to hospital will also know that they are in the study. Their medical notes may be looked at by staff from Swansea and Cardiff Universities or NHS and regulatory auditors who will be checking that the study is being done correctly. If you agree, we will also tell your family doctor (GP) that they are in the study.

People at the research laboratories will not know who your child is when they test their samples. Your child will be given a study number to replace their name so any study samples and data related to them will be anonymised. The questionnaires and the data from your flash glucose monitor will also use study numbers instead of names.

All information collected about your child during the study will be kept by the research nurse in a locked cabinet and entered onto a secure database. Only people with the password can open up the database.

Cardiff University is the sponsor for this study based in the United Kingdom. Cardiff University will be using information from your child and their medical records in order to undertake this study and will act as the data controller for this study. This means that we are responsible for looking after their information and using it properly. Cardiff University will keep identifiable information about them for 25 years after the study has finished. Your rights to access, change or move the information about your child are limited, as we need to manage their information in specific ways in order for the research to be reliable and accurate. If you/they withdraw from the study, we will keep

For peer review only - http://bmjopen.bmj.com/site/about/guidelines.xhtml

the information about them that we have already obtained. To safeguard their rights, we will use the minimum personally-identifiable information possible. You can find out more about how we use your child's information by contacting inforequest@cardiff.ac.uk.

WHAT WILL HAPPEN TO ANY SAMPLES THEY GIVE?

We want to test your child's blood and urine to better understand your diabetes and how the study medicine or placebo affects their diabetes. The blood samples will be used to test:

1. The amount of insulin their body still makes before and after taking part in the study.
2. Their average blood glucose levels
3. The antibodies to the insulin making cells
4. General health checks – anaemia, kidneys, liver etc
5. How much of the study medication is in their blood.
6. How their body's immune system is reacting to the study medication.

Scientists in laboratories around the UK will look at their anonymised blood samples. These samples will be stored in a safe place. Some of their blood samples will be sent to a laboratory in Europe and either America or Canada for special testing to find out how much of the study medicine is present.

We would like to keep any leftover blood samples in a special tissue repository permanently - we will ask for your permission to do this. The samples will only be accessed by scientists who have special permission to do so. The samples might be sent outside the UK to other research teams in Europe or countries such as America and Canada if you agree to this. These other teams must have permission from us to use your sample before we send it. If you do not want to agree to this, their samples will be destroyed after they have been analysed.

WILL ANY GENETIC TESTS BE DONE?

We will use their blood samples to help us study the genes involved with diabetes and the immune system. These samples will not have your name on them and will not be used for any other reason without your permission.

WHAT WILL HAPPEN TO THE RESULTS OF THIS STUDY?

The full results of this study will not be known until the last patient has completed their tests, which may take more than 5 years. The research results will be reported in scientific publications and meetings but you will not be identified by name at all. If you are interested in receiving a summary of the research results, we can arrange this.

WHO IS ORGANISING AND FUNDING THE STUDY?

CARDIFF UNIVERSITY PRIFYSGOL CAERDYDD

Swansea University Prifysgol Abertawe
Swansea Trials Unit Uned Dreialon Abertawe

The study is being organised by researchers at Cardiff University and Swansea University.

It is being funded by a grant from the National Institute for Health and Research (NIHR).

NHS National Institute for Health Research

WHERE IS THE STUDY BEING DONE?

The study is being done at hospitals and research centres across England, Wales and Scotland.

WHO HAS CHECKED THIS STUDY?

Before any research goes ahead it has to be checked by a Research Ethics Committee. This is a group of people who make sure that the research is OK to do and to make sure that the patient will be safe. This study has been looked at by Wales REC 3. As this study is looking at a medicine, it has also been approved by the government's Medicine and Healthcare products Regulatory Authority (MHRA) who check that the researchers carry out the study safely. It has also been checked by national and local NHS organisations to make sure that the study can be done using their site and staff.

WHAT SHOULD I DO NOW?

If you are interested in taking part, or have any questions please contact one of the following people:

Name:	Name:	Name: (only if needed)

For peer review only - http://bmjopen.bmj.com/site/about/guidelines.xhtml

**Role:** Principal Investigator<br>**Tel. No:**<br>**Email:**	**Role:** Research nurse<br>**Tel. No:**<br>**Email:**	**Role:**<br>**Tel. No:**<br>**Email:**

Alternatively, you may want to speak to someone at the USTEKID Trial Office who are managing the study, based at Swansea University. The Trial Manager's details are below:

Name: Dr Kym Thorne
Tel. No: 01792 606372 (direct) or 01792 606545 for Swansea Trials Unit
Email: ustekid@swansea.ac.uk
Address: Floor 2, Institute of Life Sciences 2, Swansea University Medical School, Singleton Park, Swansea SA2 8PP

Thank you for taking the time to read this information sheet and for considering taking part in this research study

For peer review only - http://bmjopen.bmj.com/site/about/guidelines.xhtml

[Insert local headers]

USTEKID

NHS National Institute for Health Research

CARDIFF UNIVERSITY PRIFYSGOL CAERDYDD

Swansea University Prifysgol Abertawe Swansea Trials Unit Uned Dreialon Abertawe

A research study to see if the medicine Ustekinumab can make diabetes easier to manage

<u>CONTACT DETAILS FOR STUDY TEAM</u>:

NURSE:

DOCTOR:

EMERGENCIES:

FOR PARENTS OF YOUNG PEOPLE AGED 16-18 YEARS OLD

We would like to invite your child to help us with our research study. It is important for you to understand why the research is being done and what it will involve as we also need some information from you. Please take time to read the following information carefully and discuss it with friends and relatives if you wish. If anything is unclear or you need to know more, please ask us.

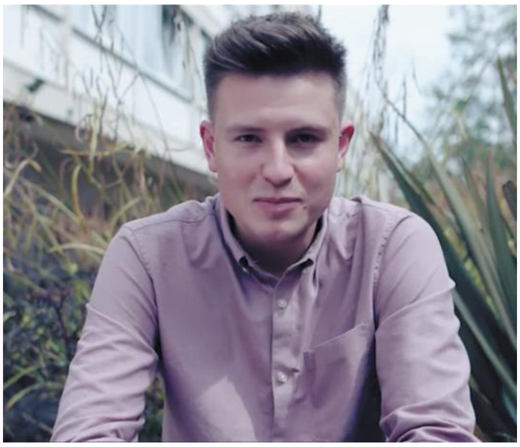

Please look at our video explaining the trial at www.type1diabetesresearch.org.uk/current-trials. The blue box below and the video contains the key points about the study. If you would like to know more, please read the rest of this leaflet.

<u>KEY POINTS ABOUT THE STUDY</u>:

USTEKID

- We want to see if the study medicine, **Ustekinumab**, can make Type 1 diabetes (the type your child has) easier to manage. The medicine works by "protecting" some of the cells in the pancreas that still produce insulin from attack by the immune system.
- The study will involve your child having an injection every 1-3 months with either the study medicine, Ustekinumab or a placebo (a "dummy medicine"). These injections are given under the skin just like insulin injections and will be done by the study doctor or nurse. Neither you nor the research team will know if your child is receiving the study medicine or the placebo.
- The medicine is already being used to treat other illnesses quite safely.
- Your child will come to your local hospital or research centre for 10 study visits over a 15 month period, but where possible this will be on the same day as their routine hospital visits. The first two will check if you are eligible to take part. Three of the visits can be sometimes done by a research nurse at home. You will be asked to come with them for three of these study visits to complete a questionnaire.
- We will ask your child to provide extra blood and urine samples for the study.
- We will provide your child with a flash blood glucose monitor (Freestyle Libre) to wear for 2 weeks before each visit. They can keep the monitor for use at home for the whole time of the study.
- Your child will be offered a small gift voucher for each visit.
- You can stop taking part in the study at any time and you do not have to give a reason why. It will not affect your child taking part in the study.

For peer review only - http://bmjopen.bmj.com/site/about/guidelines.xhtml

WHY IS THIS STUDY BEING DONE?

This study is being done to see if a medicine called **Ustekinumab** can help to "protect" the cells in the body that produce insulin in young people recently diagnosed with Type 1 Diabetes . It is caused by the body's own immune system damaging the cells in the pancreas that make insulin. Our aim is to develop a treatment that can slow this process by targeting the immune cells causing the damage.

At the time of diagnosis, most children have 10-20% of their insulin-producing cells still working. It usually takes between 1 and 5 years before they stop working completely. Sometimes these last few working cells can make enough insulin to make blood sugar levels stable and easier to control—this is called the "Honeymoon period". This period is only temporary and doesn't last. Ustekinumab, the study medicine, may make this period last longer by reducing the damaging effects of the immune system on the remaining insulin-producing cells in the pancreas.

Ustekinumab is currently given to adults and teenagers with particular skin and bowel problems and it is known to be safe and effective at treating those conditions.

WHY HAS MY CHILD BEEN ASKED TO TAKE PART?

Your child has been chosen because they are aged 12 - 18 years old and has recently been diagnosed with Type 1 diabetes. You are being told about the study because if they agree to take part, we need a parent/carer to complete three questionnaires for us.

DO I HAVE TO TAKE PART?

No. It is completely up to you whether or not you take part. It will not affect your child taking part in the study. Both you and your child are free to change your minds at any time.

If your child is interested in this study then they can let us know by calling one of the people listed at the end of their information sheet and a member of the research team will contact them and you to explain more about the study and answer any questions you may both have.

WHAT HAPPENS ONCE MY CHILD AGREEDS TO TAKE PART?

Before we can start the treatment, we need to check that your child is eligible to take part in the study - this is called "screening". Your child will be asked to come to your local hospital or research centre to talk about the study. This is where you will both be able to ask questions.

You will be invited to attend the screening visits and asked to consider whether you would be happy to consent to completing three questionnaires, one at the second screening visit, one 28 weeks after their first study treatment and one 52 weeks after their first study treatment. The person consenting should be the person who will complete all three study questionnaires. If you are happy to do so, we will ask you to sign a consent form and begin completing the first questionnaire at the second screening visit.

If you would like more details on your child's role in the study, please ask to see their information sheet which details the visits and tests being done.

WHAT WILL I BE ASKED TO DO?

As well as attending with your child to your local hospital or research centre for study visits that require you to complete questionnaires, there are a few things we will ask your child to do during the study which may involve your help:

- Your child will be asked to complete a diary between study visits to record:
  a) How much insulin they take during the study (for the two weeks before every study visit)
  b) If they feel unwell or have to take any medicines during the study.
  c) If they have any hypoglycaemic (low blood glucose level) episodes that need treating.
- We will ask them to test their blood glucose levels at home for at least 2 weeks before each visit using our FREE Abbott Freestyle Libre glucose monitoring system so they don't need to do extra finger prick tests for the trial. They will need to wear a sensor on their arm. We will show you both how it works. They are free to use the monitor at home for the rest of the time of study if they want to. We will give them enough sensors to allow this for a year.

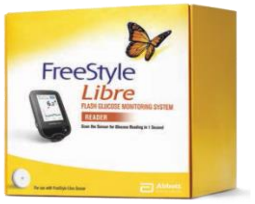

For peer review only - http://bmjopen.bmj.com/site/about/guidelines.xhtml

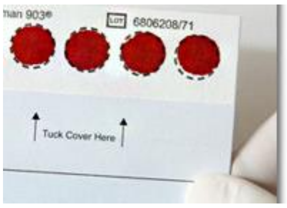

- We will ask them to give a blood spot sample which they can do at home. They will need to do this once a week for 28 weekly then every month for the next 6 months. We will show you both how to do this and will provide envelopes and pay for the postage.
- You will be asked to make sure that your child does not have certain vaccinations before, during and immediately after the study. If they need a vaccination, for example if they are travelling abroad, you must tell the study doctor or nurse immediately.

WILL THE STUDY HELP MY CHILD?

Your child has been allocated to the group that receives the study medicine Ustekinumab, it is possible that it will help their pancreas make insulin for longer. However we cannot say this for certain until we have completed this study. During the study your child's diabetes will be very closely monitored. This will include regular check-ups with your local diabetes team including routine blood testing. You and your child will have more time with the research team to discuss their diabetes and ask questions than at a normal clinic appointment.

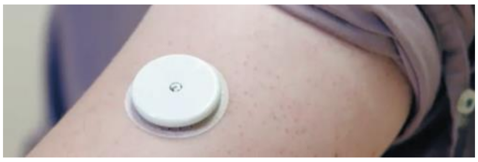

Your child will be provided with a FREE Abbott Freestyle Libre blood glucose monitoring system. They can use this to check their blood glucose levels while they are in the study.

Injections with the study medicine or the placebo will be done at weeks 0, 4, 12, 20, 28, 36 and 44. After this, no more treatments will be given to your child.

WHAT HAPPENS WHEN THE STUDY STOPS?

We will collect all the information together and we will decide if the study medicine can help people with Type 1 Diabetes make their own insulin for longer. If it does then we will carry out a bigger version of this study.

WHAT IF NEW INFORMATION COMES ALONG?

Sometimes during research, we get new information about the treatment being studied. If this happens, we will tell you about it and discuss whether you want to continue in the study.

WHAT IF I DO NOT WANT TO TAKE PART ANYMORE?

Just let the study doctor or research nurse know about your decision and we will make sure that you are not asked to complete further questionnaires. If you withdraw, this will not affect your child taking part in the study.

WHAT IF THERE IS A PROBLEM OR SOMETHING GOES WRONG?

If there are any problems, please contact the study team using the details at the end of this form.

If you feel overwhelmed by your child's recent diagnosis, you can call the local [title] on [tel number / email] and they can talk through your concerns with you.

If you are unhappy about the conduct of the study and wish to complain, you can do this through: (name and contact details of appropriate organisations – site specific).

WHAT ARE THE POSSIBLE SIDE EFFECTS FOR ME IN PARTICIPATING IN THE STUDY?

You are only being asked to complete three short questionnaires. We do not anticipate any risk being involved.

The risks for your child for taking part in the study are fully explained in their information sheet.

WILL I RECEIVE ANY PAYMENT FOR TAKING PART?

You will be able to claim back your travel expenses getting your child to the local hospital or research centre.

WHAT INFORMATION WILL YOU COLLECT AND HOW WILL IT BE KEPT PRIVATE?

We will ask for your name and contact details as well as your child's name so that the research nurse can keep in touch and manage your visits. We will also collect the information you record on your questionnaire and enter it into a database.

The people in our research team at the local hospital or research centre will know that your child is taking part. The doctors looking after your child when you come to hospital will also know that they are in the study. Their medical

For peer review only - http://bmjopen.bmj.com/site/about/guidelines.xhtml

notes may be looked at by staff from Swansea and Cardiff Universities who will be checking that the study is being done correctly. If your child has agreed, we will also tell your family doctor (GP) that they are in the study.

You and your child will be given a study number to replace their name so any study samples and information related to you both will be anonymised.

All information collected about you and your child during the study will be kept by the research nurse in a locked cabinet and entered onto a secure database. Only people with the password can open up the database.

Cardiff University is the sponsor for this study based in the United Kingdom. Cardiff University will be using information from you and your medical records in order to undertake this study and will act as the data controller for this study. This means that we are responsible for looking after your information and using it properly. Cardiff University will keep identifiable information about you for 25 years after the study has finished.

Your rights to access, change or move your information are limited, as we need to manage your information in specific ways in order for the research to be reliable and accurate. If you withdraw from the study, we will keep the information about you that we have already obtained. To safeguard your rights, we will use the minimum personally-identifiable information possible. You can find out more about how we use your information by contacting inforequest@cardiff.ac.uk.

WHAT WILL HAPPEN TO THE RESULTS OF THIS STUDY?

The full results of this study will not be known until the last patient has completed their tests, which may take more than 5 years. The research results will be reported in scientific publications and meetings but you will not be identified by name at all. If you are interested in receiving a summary of the research results, we can arrange this.

WHO IS ORGANISING AND FUNDING THE STUDY?

The study is being organised by researchers at Cardiff University and Swansea University. It is being funded by a grant from the National Institute for Health and Research (NIHR).

WHERE IS THE STUDY BEING DONE?

The study is being done at hospitals and research centres across England, Wales and Scotland.

WHO HAS CHECKED THIS STUDY?

Before any research goes ahead it has to be checked by a Research Ethics Committee. This is a group of people who make sure that the research is OK to do and to make sure that the patient will be safe. This study has been looked at by Wales REC 3. As this study is looking at a medicine, it has also been approved by the government's Medicine and Healthcare products Regulatory Authority (MHRA) who check that the researchers carry out the study safely. It has also been checked by national and local NHS organisations to make sure that the study can be done using their site and staff.

WHAT SHOULD I DO NOW?

If you are interested in taking part, or have any questions please contact one of the following people:

**Name:**	**Name:**	**Name:** (only if needed)
**Role:** Principal Investigator	**Role:** Research nurse	**Role:**
**Tel. No:**	**Tel. No:**	**Tel. No:**
**Email:**	**Email:**	**Email:**

Alternatively, you may want to speak to someone at the USTEKID Trial Office who are managing the study, based at Swansea University. The Trial Manager's details are below:

Name: Dr Kym Thorne
Tel. No: 01792 606372 (direct) or 01792 606545 for Swansea Trials Unit
Email: ustekid@swansea.ac.uk
Address: Floor 2, Institute of Life Sciences 2, Swansea University Medical School, Singleton Park, Swansea SA2 8PP

Thank you for taking the time to read this information sheet and for considering taking part in this research study

For peer review only - http://bmjopen.bmj.com/site/about/guidelines.xhtml

USTEKID

Phase II multi-centre, double-blind, randomised trial of Ustekinumab in adolescents with new-onset type 1 diabetes (USTEKID)

**Chief Investigator:**	Prof Colin Dayan	**Principal Investigator:**	
**Site ID:**		**Participant study number:**	
**Participant name (in capitals):**			

ASSENT FORM FOR YOUNG ADOLESCENTS (AGED 12-15y)

(to be completed by the child with help from and their parent/carer)

Circle either Yes or No to the following

Have you read the information sheet about the study (or has someone read it to you)?	YES	NO
Has somebody explained this study to you?	YES	NO
Do you understand what this study is about?	YES	NO
Have you asked all the questions you want?	YES	NO
Has someone answered your questions in a way that you understand?	YES	NO
Do you understand that it is OK to stop taking part at any time?	YES	NO
Are you happy to give samples of blood and wee (urine)?	YES	NO
Are you happy to take part?	YES	NO

If you do not want to take part, do not sign your name.
If you are not happy with what you have circled YES to, do not sign your name.

If you do want to take part, you should write your name clearly below.

Your name ____

Please write the date ____________________

The person who explained the study to you also needs to sign this form:

____________________ ____________________ ____________________

Researcher Name Signature Date

For peer review only

For peer review only - http://bmjopen.bmj.com/site/about/guidelines.xhtml

Insert logos

Phase II multi-centre, double-blind, randomised trial of Ustekinumab in adolescents with new-onset type 1 diabetes (USTEKID)

Chief Investigator: Prof Colin Dayan **Principal Investigator:**

Site ID: **Participant study number:**

CONSENT FORM FOR ADOLESCENTS (AGED 16-18y)

Please initial boxes

1. I confirm that I have read and understand the 16-18y Patient Information Sheet dated (………………) (version......) for the above study. I have had the opportunity to consider the information, ask questions and have had these answered satisfactorily.
2. I understand that my participation is voluntary and that I am free to withdraw at any time without giving any reason, without my medical care or legal rights being affected.
3. I agree to attend screening and study visits and to being randomised to receive either the study medicine or the placebo.
4. I agree to provide urine and blood samples for the study.
5. I agree to do dried blood spot testing at home for the study.
6. I agree to wear the FreeStyle Libre glucose monitor at least two weeks prior to each study visit.
7. I agree to complete diaries and questionnaires for the study.
8. I agree that if I am involved in actions that may lead to pregnancy, I will take adequate contraception (hormonal based contraception, barrier contraception, abstinence) until 4 months following the date of final treatment.
9. I give permission for relevant sections of my medical notes and data collected during the study to be looked at by responsible individuals from the USTEKID research team, from regulatory authorities or from Cardiff University (as Sponsor), where it is relevant to my taking part in this research.
10. I understand that the information collected about me may be used to support other research in the future, and may be shared anonymously with other researchers.
11. I understand that the information held and maintained in local hospital records and other central UK NHS bodies may be used to help contact me or provide information about my health status during the study follow up.
12. I understand and agree that my anonymised blood samples may be used for analysis by the study or other relevant studies if they obtain the relevant permissions.
13. I agree to my anonymised blood samples being stored in a Human Tissue Authority (HTA) repository for future ethically approved studies.
14. I agree that my anonymised blood samples may be transported within and outside the European Union for analysis in specialist laboratories.
15. I agree to my GP being notified of my involvement in the study, including any necessary exchange of information about me between my GP and the research team.
16. I agree to take part in the above study.

For the participant

NAME____________________________________SIGNATURE________________________ DATE___________________

For the person taking consent

NAME____________________________________SIGNATURE________________________ DATE___________________

For peer review only - http://bmjopen.bmj.com/site/about/guidelines.xhtml

Insert logos

Phase II multi-centre, double-blind, randomised trial of Ustekinumab in adolescents with new-onset type 1 diabetes (USTEKID)

Chief Investigator: Prof Colin Dayan **Principal Investigator:**

Site ID: **Participant study number:**

CONSENT FORM FOR PARENTS/CARERS OF YOUNG ADOLESCENTS (AGED 12-15y)

Please initial boxes

1. I confirm that I have read and understand the 12-15y Parent Information Sheet dated (………………) (version......) for the above study. I have had the opportunity to consider the information, ask questions and have had these answered satisfactorily.
2. I understand that my child's participation is voluntary and that we are free to withdraw at any time without giving any reason, without his/her medical care or legal rights being affected.
3. I agree to my child attending screening and study visits and to being randomised to receive either the study medicine or the placebo.
4. I agree that my child can provide urine and blood samples for the study.
5. I agree that my child can do dried blood spot testing at home for the study.
6. I agree that my child can wear the FreeStyle Libre glucose monitor at least two weeks prior to each study visit.
7. I agree that my child can complete diaries and questionnaires for the study.
8. I agree to complete study questionnaires myself.
9. I understand that the information collected from me and my child in study questionnaires will be viewed by the research team and will be stored securely.
10. I agree that if my child is involved in actions which may lead to pregnancy, they will take adequate contraception (hormonal based contraception, barrier contraception, abstinence) until 4 months following the date of final treatment.
11. I give permission for relevant sections of my child's medical notes and data collected during the study to be looked at by responsible individuals from the USTEKID research team, from regulatory authorities or from Cardiff University (as Sponsor), where it is relevant to my child taking part in this research.
12. I understand that the information collected about my child may be used to support other research in the future, and may be shared anonymously with other researchers.
13. I understand that the information held and maintained in local hospital records and other central UK NHS bodies may be used to help contact myself or my child, or to provide information about my child's health status during the study follow up.
14. I understand and agree that my child's anonymised blood samples may be used for analysis by the study or other relevant studies if they obtain the relevant permissions.
15. I agree to my child's anonymised blood samples being stored in a Human Tissue Authority (HTA) repository for future ethically approved studies.
16. I agree that my child's anonymised blood samples may be transported within and outside the European Union for analysis in specialist laboratories.
17. I agree to my child's GP being notified of his/her involvement in the study, including any necessary exchange of information about my child between my GP and the research team.
18. I agree to my child taking part in the above study.

For the participant's parent/carer

NAME____________________________SIGNATURE_________________________ DATE________________

For the person taking consent

NAME____________________________SIGNATURE_________________________ DATE________________

For peer review only - http://bmjopen.bmj.com/site/about/guidelines.xhtml

Phase II multi-centre, double-blind, randomised trial of Ustekinumab in adolescents with new-onset type 1 diabetes (USTEKID)

**Chief Investigator:**	Prof Colin Dayan	**Principal Investigator:**	Prof John Gregory
**Site ID:**		**Participant study number:**	
**Participant name (in capitals):**			

CONSENT FORM FOR PARENT/CARER OF ADOLESCENTS (AGED 16-18y)

Please initial the boxes

1. I confirm that I have read and understand the 16-18y Parent Information Sheet dated 2nd July 2019, version 3 for the above study. I have had the opportunity to consider the information, ask questions and have had these answered satisfactorily. ☐
2. I understand that my participation is voluntary and that I am free to withdraw at any time without giving any reason, without my child's medical care or legal rights being affected. ☐
3. I agree to complete study questionnaires. ☐
4. I understand that the information collected from me in study questionnaires will be viewed by the research team and will be stored securely. ☐
5. I agree to take part in the above study. ☐

For the participant's parent/carer

NAME____________________________ SIGNATURE_________________________ DATE________________

For the person taking consent

NAME____________________________ SIGNATURE_________________________ DATE________________

For peer review only - http://bmjopen.bmj.com/site/about/guidelines.xhtml

Reporting checklist for protocol of a clinical trial.

Based on the SPIRIT guidelines.

Instructions to authors

Complete this checklist by entering the page numbers from your manuscript where readers will find each of the items listed below.

Your article may not currently address all the items on the checklist. Please modify your text to include the missing information. If you are certain that an item does not apply, please write "n/a" and provide a short explanation.

Upload your completed checklist as an extra file when you submit to a journal.

In your methods section, say that you used the SPIRIT reporting guidelines, and cite them as:

Chan A-W, Tetzlaff JM, Gøtzsche PC, Altman DG, Mann H, Berlin J, Dickersin K, Hróbjartsson A, Schulz KF, Parulekar WR, Krleža-Jerić K, Laupacis A, Moher D. SPIRIT 2013 Explanation and Elaboration: Guidance for protocols of clinical trials. BMJ. 2013;346:e7586

		Reporting Item	Page Number
**Administrative information**			
Title	#1	Descriptive title identifying the study design, population, interventions, and, if applicable, trial acronym	1

For peer review only

Trial registration	#2a	Trial identifier and registry name. If not yet registered, name of intended registry	2
Trial registration: data set	#2b	All items from the World Health Organization Trial Registration Data Set	Not included – repository agreement not currently in place
Protocol version	#3	Date and version identifier	2
Funding	#4	Sources and types of financial, material, and other support	14
Roles and responsibilities: contributorship	#5a	Names, affiliations, and roles of protocol contributors	1 & 15
Roles and responsibilities: sponsor contact information	#5b	Name and contact information for the trial sponsor	14
Roles and responsibilities: sponsor and funder	#5c	Role of study sponsor and funders, if any, in study design; collection, management, analysis, and interpretation of data; writing of the report; and the decision to submit the report for publication, including whether they will have ultimate authority over any of these activities	14

For peer review only

Roles and responsibilities: committees	#5d	Composition, roles, and responsibilities of the coordinating centre, steering committee, endpoint adjudication committee, data management team, and other individuals or groups overseeing the trial, if applicable (see Item 21a for data monitoring committee)	14
**Introduction**			
Background and rationale	#6a	Description of research question and justification for undertaking the trial, including summary of relevant studies (published and unpublished) examining benefits and harms for each intervention	3
Background and rationale: choice of comparators	#6b	Explanation for choice of comparators	3
Objectives	#7	Specific objectives or hypotheses	3 and Table 1
Trial design	#8	Description of trial design including type of trial (eg, parallel group, crossover, factorial, single group), allocation ratio, and framework (eg, superiority, equivalence, non-inferiority, exploratory)	3
**Methods: Participants, interventions, and outcomes**			

For peer review only

Study setting	#9	Description of study settings (eg, community clinic, academic hospital) and list of countries where data will be collected. Reference to where list of study sites can be obtained	3
Eligibility criteria	#10	Inclusion and exclusion criteria for participants. If applicable, eligibility criteria for study centres and individuals who will perform the interventions (eg, surgeons, psychotherapists)	5 and Table 2
Interventions: description	#11a	Interventions for each group with sufficient detail to allow replication, including how and when they will be administered	10
Interventions: modifications	#11b	Criteria for discontinuing or modifying allocated interventions for a given trial participant (eg, drug dose change in response to harms, participant request, or improving / worsening disease)	11
Interventions: adherance	#11c	Strategies to improve adherence to intervention protocols, and any procedures for monitoring adherence (eg, drug tablet return; laboratory tests)	Not applicable. Intervention given at site
Interventions: concomitant care	#11d	Relevant concomitant care and interventions that are permitted or prohibited during the trial	Table 2
Outcomes	#12	Primary, secondary, and other outcomes, including the specific measurement variable (eg, systolic	Table 1

For peer review only

		blood pressure), analysis metric (eg, change from baseline, final value, time to event), method of aggregation (eg, median, proportion), and time point for each outcome. Explanation of the clinical relevance of chosen efficacy and harm outcomes is strongly recommended	
Participant timeline	#13	Time schedule of enrolment, interventions (including any run-ins and washouts), assessments, and visits for participants. A schematic diagram is highly recommended (see Figure)	Table 3, Figure 1
Sample size	#14	Estimated number of participants needed to achieve study objectives and how it was determined, including clinical and statistical assumptions supporting any sample size calculations	12
Recruitment	#15	Strategies for achieving adequate participant enrolment to reach target sample size	5
**Methods: Assignment of interventions (for controlled trials)**			
Allocation: sequence generation	#16a	Method of generating the allocation sequence (eg, computer-generated random numbers), and list of any factors for stratification. To reduce predictability of a random sequence, details of any planned	7

For peer review only

		restriction (eg, blocking) should be provided in a separate document that is unavailable to those who enrol participants or assign interventions	
Allocation concealment mechanism	#16b	Mechanism of implementing the allocation sequence (eg, central telephone; sequentially numbered, opaque, sealed envelopes), describing any steps to conceal the sequence until interventions are assigned	7
Allocation: implementation	#16c	Who will generate the allocation sequence, who will enrol participants, and who will assign participants to interventions	7
Blinding (masking)	#17a	Who will be blinded after assignment to interventions (eg, trial participants, care providers, outcome assessors, data analysts), and how	7
Blinding (masking): emergency unblinding	#17b	If blinded, circumstances under which unblinding is permissible, and procedure for revealing a participant's allocated intervention during the trial	7
**Methods: Data collection, management, and analysis**			
Data collection plan	#18a	Plans for assessment and collection of outcome, baseline, and other trial data, including any related processes to promote data quality (eg, duplicate	8-10

For peer review only

		measurements, training of assessors) and a description of study instruments (eg, questionnaires, laboratory tests) along with their reliability and validity, if known. Reference to where data collection forms can be found, if not in the protocol	
Data collection plan: retention	#18b	Plans to promote participant retention and complete follow-up, including list of any outcome data to be collected for participants who discontinue or deviate from intervention protocols	10
Data management	#19	Plans for data entry, coding, security, and storage, including any related processes to promote data quality (eg, double data entry; range checks for data values). Reference to where details of data management procedures can be found, if not in the protocol	13-4
Statistics: outcomes	#20a	Statistical methods for analysing primary and secondary outcomes. Reference to where other details of the statistical analysis plan can be found, if not in the protocol	12 and Table 1
Statistics: additional analyses	#20b	Methods for any additional analyses (eg, subgroup and adjusted analyses)	12
Statistics: analysis population and missing data	#20c	Definition of analysis population relating to protocol non-adherence (eg, as randomised analysis), and	12

For peer review only

		any statistical methods to handle missing data (eg, multiple imputation)	
**Methods: Monitoring**			
Data monitoring: formal committee	#21a	Composition of data monitoring committee (DMC); summary of its role and reporting structure; statement of whether it is independent from the sponsor and competing interests; and reference to where further details about its charter can be found, if not in the protocol. Alternatively, an explanation of why a DMC is not needed	14
Data monitoring: interim analysis	#21b	Description of any interim analyses and stopping guidelines, including who will have access to these interim results and make the final decision to terminate the trial	12
Harms	#22	Plans for collecting, assessing, reporting, and managing solicited and spontaneously reported adverse events and other unintended effects of trial interventions or trial conduct	11
Auditing	#23	Frequency and procedures for auditing trial conduct, if any, and whether the process will be independent from investigators and the sponsor	13
**Ethics and dissemination**			

For peer review only

Research ethics approval	#24	Plans for seeking research ethics committee / institutional review board (REC / IRB) approval	14
Protocol amendments	#25	Plans for communicating important protocol modifications (eg, changes to eligibility criteria, outcomes, analyses) to relevant parties (eg, investigators, REC / IRBs, trial participants, trial registries, journals, regulators)	14
Consent or assent	#26a	Who will obtain informed consent or assent from potential trial participants or authorised surrogates, and how (see Item 32)	5
Consent or assent: ancillary studies	#26b	Additional consent provisions for collection and use of participant data and biological specimens in ancillary studies, if applicable	Not applicable
Confidentiality	#27	How personal information about potential and enrolled participants will be collected, shared, and maintained in order to protect confidentiality before, during, and after the trial	11
Declaration of interests	#28	Financial and other competing interests for principal investigators for the overall trial and each study site	14
Data access	#29	Statement of who will have access to the final trial dataset, and disclosure of contractual agreements that limit such access for investigators	13

For peer review only

Ancillary and post trial care	#30	Provisions, if any, for ancillary and post-trial care, and for compensation to those who suffer harm from trial participation	11
Dissemination policy: trial results	#31a	Plans for investigators and sponsor to communicate trial results to participants, healthcare professionals, the public, and other relevant groups (eg, via publication, reporting in results databases, or other data sharing arrangements), including any publication restrictions	13
Dissemination policy: authorship	#31b	Authorship eligibility guidelines and any intended use of professional writers	13
Dissemination policy: reproducible research	#31c	Plans, if any, for granting public access to the full protocol, participant-level dataset, and statistical code	Not yet agreed.
**Appendices**			
Informed consent materials	#32	Model consent form and other related documentation given to participants and authorised surrogates	All 8 can be provided if requested
Biological specimens	#33	Plans for collection, laboratory evaluation, and storage of biological specimens for genetic or molecular analysis in the current trial and for future use in ancillary studies, if applicable	8-10

For peer review only

None The SPIRIT checklist is distributed under the terms of the Creative Commons Attribution License CC-BY-ND 3.0. This checklist can be completed online using https://www.goodreports.org/, a tool made by the EQUATOR Network in collaboration with Penelope.ai

For peer review only

BMJ Open

Phase II multi-centre, double-blind, randomised trial of ustekinumab in adolescents with new-onset Type 1 Diabetes (USTEK1D): Trial Protocol

Journal:	*BMJ Open*
Manuscript ID	bmjopen-2021-049595.R1
Article Type:	Protocol
Date Submitted by the Author:	24-Aug-2021
Complete List of Authors:	Gregory, John; Cardiff University, Division of Population Medicine<br>Carter, Kymberley; Swansea University, Swansea Trials Unit<br>Cheung, Ivy; Swansea University, Diabetes Research Group<br>Holland, Gail; Swansea University, Swansea Trials Unit;<br>Bowen-Morris, Jane; Cardiff University, Diabetes Research Group<br>Luzio, Stephen; Swansea University, Diabetes Research Group<br>Dunseath, Gareth; Swansea University, Diabetes Research Group<br>Tree, Timothy<br>Yang, Jennie; King's College London, Department of Immunobiology<br>Marwaha, Ashish; University of Toronto, Department of Molecular Genetics<br>Ali, Mohammad; Cardiff University, Diabetes Research Group<br>Bashir, Nadim; Swansea University, Trials Unit<br>Hutchings, Hayley; Swansea University, Swansea Trials Unit<br>Fegan, Greg; Swansea University, Swansea Trials Unit<br>Stenson, Rachel; Cardiff University, Diabetes Research Group<br>Hiles, Stephen; Swansea University, Swansea Trials Unit<br>Marques-Jones, Susie; Patient and Public Representative<br>Brown, Amy; Patient and Public Representative<br>Tatovic, Danijela; Cardiff University, Diabetes Research Group<br>Dayan, Colin; Cardiff University School of Medicine
**Primary Subject Heading**:	Diabetes and endocrinology
Secondary Subject Heading:	Paediatrics
Keywords:	Paediatric endocrinology < DIABETES & ENDOCRINOLOGY, STATISTICS & RESEARCH METHODS, Diabetes & endocrinology < INTERNAL MEDICINE

SCHOLARONE™
Manuscripts

I, the Submitting Author has the right to grant and does grant on behalf of all authors of the Work (as defined in the below author licence), an exclusive licence and/or a non-exclusive licence for contributions from authors who are: i) UK Crown employees; ii) where BMJ has agreed a CC-BY licence shall apply, and/or iii) in accordance with the terms applicable for US Federal Government officers or employees acting as part of their official duties; on a worldwide, perpetual, irrevocable, royalty-free basis to BMJ Publishing Group Ltd ("BMJ") its licensees and where the relevant Journal is co-owned by BMJ to the co-owners of the Journal, to publish the Work in this journal and any other BMJ products and to exploit all rights, as set out in our licence.

The Submitting Author accepts and understands that any supply made under these terms is made by BMJ to the Submitting Author unless you are acting as an employee on behalf of your employer or a postgraduate student of an affiliated institution which is paying any applicable article publishing charge ("APC") for Open Access articles. Where the Submitting Author wishes to make the Work available on an Open Access basis (and intends to pay the relevant APC), the terms of reuse of such Open Access shall be governed by a Creative Commons licence – details of these licences and which Creative Commons licence will apply to this Work are set out in our licence referred to above.

Other than as permitted in any relevant BMJ Author's Self Archiving Policies, I confirm this Work has not been accepted for publication elsewhere, is not being considered for publication elsewhere and does not duplicate material already published. I confirm all authors consent to publication of this Work and authorise the granting of this licence.

For peer review only

Phase II multi-centre, double-blind, randomised trial of ustekinumab in adolescents with new-onset Type 1 Diabetes (USTEK1D): Trial Protocol

Corresponding author: Dr Kym Carter, Swansea Trials Unit, Floor 2, ILS2, Swansea University Medical School, Singleton Park, Swansea, UK, SA2 8PP k.carter@swansea.ac.uk

John W. Gregory [1]

Kym Carter [2]

Wai Yee Cheung [3]

Gail Holland [2]

Jane Bowen-Morris [4]

Steve Luzio [3]

Gareth Dunseath [3]

Tim I.M. Tree [5]

Jennie H. M. Yang [5]

Ashish Marwaha [6]

Mohammad Alhadj Ali [4]

Nadim M. Bashir [2]

Hayley A. Hutchings [2]

Greg W. Fegan [2]

Rachel Stenson [4]

Stephen Hiles [2]

Susie Marques-Jones [7]

Amy Brown [7]

Danijela Tatovic [4]

Colin M. Dayan [4]

1 Division of Population Medicine, Cardiff University School of Medicine, Cardiff, UK

2 Swansea Trials Unit, Swansea University Medical School, Singleton Park, Swansea, UK

3 Diabetes Research Unit Cymru, Swansea University Medical School, Singleton Park, Swansea, UK

4 Diabetes Research Group, Cardiff University School of Medicine, Cardiff, UK

5 Department of Immunobiology, Faculty of Life Sciences and Medicine, King's College London, London, UK

6 Department of Molecular Genetics, University of Toronto, Toronto, Ontario, Canada

7 Patient / Parent representatives with no affiliation to an organisation

Word count, excluding title page, abstract, references, figures and tables: 4118

For peer review only - http://bmjopen.bmj.com/site/about/guidelines.xhtml

For peer review only

ABSTRACT

Introduction

Most individuals newly diagnosed with Type 1 Diabetes (T1D) have 10-20% of beta-cell function remaining at the time of diagnosis. Preservation of residual beta-cell function at diagnosis, may improve glycaemic control and reduce longer term complications.

Immunotherapy has the potential to preserve endogenous beta-cell function and thereby improve metabolic control even in poorly compliant individuals. We propose to test ustekinumab (STELARA®), a targeted and well-tolerated therapy that may halt T-cell and cytokine-mediated destruction of beta-cells in the pancreas at the time of diagnosis.

Methods and analysis

This is a double-blind Phase II study to assess the safety and efficacy of ustekinumab in 72 children and adolescents aged 12-18 with new-onset T1D.

Participants should have evidence of residual functioning beta-cells (serum C-peptide level >0.2nmol/L in the Mixed Meal Tolerance Test (MMTT) and be positive for at least one islet autoantibody (GAD, IA-2, ZnT8) to be eligible.

Participants will be given ustekinumab/placebo subcutaneously at weeks 0, 4 and 12, 20, 28, 36 and 44 in a dose depending on the body weight and will be followed for 12 months after dose 1.

MMTTs will be used to measure the efficacy of ustekinumab for preserving C-peptide area under the curve at week 52 compared with placebo. Secondary objectives include further investigations into the efficacy and safety of ustekinumab, patient and parent questionnaires, alternative methods for measuring insulin production and exploratory mechanistic work.

Ethics and dissemination.

This trial received research ethics approval from the Wales Research Ethics Committee 3 in September 2018 and began recruiting in December 2018.

The results will be disseminated using highly accessed, peer-reviewed medical journals and presented at conferences.

Trial ISRCTN ID: 14274380.

Protocol v4.0 dated 4th May 2020.

STRENGTHS AND LIMITATIONS OF THIS STUDY

- This trial is being undertaken in 16 sites across the UK (England = 12, Scotland = 2 and Wales = 2) with a recruitment period of three years due to temporary closure of recruiting sites caused by COVID infection.
- The trial will provide evidence of the efficacy and safety of treating new onset T1D in 12-18 year olds with ustekinumab (Stelara ®).
- We have included an extensive range of secondary and exploratory outcomes to investigate the efficacy and safety of ustekinumab, and to expand existing methodological designs in this field.

For peer review only

For peer review only - http://bmjopen.bmj.com/site/about/guidelines.xhtml

INTRODUCTION

Nearly 100 years after the discovery of insulin, over 70% of patients with Type 1 diabetes (T1D) continue to have unsatisfactory glycaemic control putting them at risk of long-term complications.(1) Tragically, death rates amongst adolescents have not improved in the last few decades (1968-2009).(2) Despite major advances in closed loop insulin pump therapy, much of the morbidity arises from young people failing to engage with complex therapies.

Most individuals have 10-20% of beta-cell function remaining at the time of diagnosis of T1D.(3) Preservation of even 5% of beta-cell function has been shown to lower blood glucose levels (as measured by HbA1c tests) by 1%, permit over 50% of people to reach target glycaemic levels, reduce hypoglycaemic risk by >50% and reduce long-term complications by 50%.(4, 5) Immunotherapy has the potential to preserve endogenous beta-cell function and thereby improve metabolic control even in poorly compliant individuals. (6-8).

Novel low-risk targeted biologic therapies are widely used in other autoimmune diseases such as rheumatoid arthritis, psoriasis and inflammatory bowel disease, but no treatment was licensed for use in T1D. Ustekinumab is licenced in the UK for the treatment of psoriasis in children and adults, psoriatic arthritis in adults and Crohn's disease in adults.

Extensive evidence exists to implicate two major autoimmune cytokine pathways, IL-12/IFN-γ and IL-23/IL-17, in beta cell destruction. Ustekinumab (STELARA®), binds and inhibits the p40 molecular subunits of both IL-12 and IL-23 thus blocking their action in inducing pathogenic CD4 Th1 and Th17 T cell subsets.(9) Our overarching hypothesis is that interrupting the IL-17 and IFN-γ axes in individuals with recent-onset T1D will halt or slow the autoimmune destruction of beta cells sufficiently to permit beta cell preservation and maintain residual physiological insulin secretion. Given the therapeutic success of biologics that target immune molecules in other autoimmune and inflammatory diseases, and the evidence that IL-17 and IFN-γ producing cells are pathogenic to beta cells, we propose that ustekinumab may be beneficial for the treatment of T1D.

This paper presents the protocol for a double blind, multicentre, randomised phase II trial to evaluate the effect of ustekinumab in patients aged 12-18 years with new onset T1D.

METHODS

Overview

This is a multicentre, double blind, randomised, controlled trial comparing ustekinumab with placebo (2:1 ratio). Doses of ustekinumab will be 2mg/Kg body weight if the child is ≤40Kg and 90mg if >40Kg. Doses will be administered at week 0, 4, 12, 20, 28, 36 and 44 and with follow up at week 52 (see Figure 1). The study will be carried out in 12 to 18-year-olds within 100 days of diagnosis of T1D in 16 sites across mainland UK.

The primary objective is to determine the efficacy of ustekinumab for preserving mixed meal tolerance test (MMTT) stimulated 2-hour plasma C-peptide area under the curve (AUC) at week 52 as compared to placebo. This follows the rationale published by Greenbaum. (10)

Objectives

Table 1 details the trial objectives, outcome measures and time points for data analysis.

Objectives	Outcome Measures
**Primary Objective**	

For peer review only - http://bmjopen.bmj.com/site/about/guidelines.xhtml

Objectives	Outcome Measures
To determine the efficacy of ustekinumab (dose: 2mg/kg (≤40kg); 90mg (>40kg)) for preserving MMTT stimulated 2-hour C-peptide area under the curve (AUC) at week 52 as compared to placebo in children and adolescents with new-onset T1D.	MMTT C-peptide AUC values at week 52
**Secondary Objectives**	
1. To determine the efficacy of the ustekinumab dosing to elicit response to treatment.	Number of responders (defined as participant who has HbA1c ≤ 48mmol/mol and mean daily insulin use <0.5 IU/kg/day) measured over 7 consecutive days during the 2 weeks preceding the visit in treatment and placebo group at week 52
2. To investigate additional efficacy (metabolic) endpoints including MMTT C-peptide AUC at week 28, HbA1c and insulin use measurements at week 52.	MMTT C-peptide AUC values at Week 28
	HbA1c at weeks 0, 12, 28 and 52
	Exogenous insulin requirement as reflected in mean daily insulin usage over 7 consecutive days (IU units/kg body weight/day) as recorded in diaries prior to study visits at weeks 12, 28 and 52
	Insulin dose adjusted HbA1c (IDAAC) at week 52
3. To compare alternative metabolic endpoint assays to MMTT: including glycaemic variability in glucose monitoring systems –Freestyle Libre) and hypoglycaemia rates.	Glycaemic variability parameters downloaded from glucose monitoring at each study visit, e.g.<br>• Blood glucose level at 1,2,3 hours before and after each meal<br>• Number of episodes and length of time within the following glucose level: below 4.0 mmol/L, >10 mmol/L and >15 mmol/L<br>• % Time hypoglycaemic (<3.0 mmol/ and <4.0 mmol)
	Clinical hypoglycaemic events determined by patient diary reports and AE reports at week 52
4. To determine the safety of ustekinumab dose in adolescents with new-onset T1D.	Frequency and severity of all adverse events at week 52
5. To compare between treatment arms and across the course of treatment the age appropriate PROMs scores completed by participants and parents/carers.	HYPOFEAR, DTSQ, and PedsQL questionnaires completed by participants and their parent/carer at weeks -2, 28 and 52
**Exploratory objectives**	
1. To investigate alternative ways of measuring insulin production other than MMTT C-peptide.	Proinsulin, Glucagon, somatostatin levels, Dried blood spot (DBS) C-peptide at weeks 28 and 52
	DBS C-peptide vs MMTT C-peptide at weeks -2, 28 and 52

For peer review only - http://bmjopen.bmj.com/site/about/guidelines.xhtml

Objectives	Outcome Measures
2. To investigate changes in relevant immune mechanistic parameters include flow cytometry immune phenotyping of all IL-17 and IFN-gamma secreting T cell subsets, fluorospot analysis for IL-17 and IFN-gamma secretion in response to antigens for CD4+ T cells.	Changes at weeks 12, 28 and 52 in:<br>• immune phenotype of all IL-17, IFN-g secreting immune subsets<br>• T cell responses to antigens or peptides derived from islet antigens (including proinsulin, GAD and IA-2) measured by cytokine FLUOROSPOT (IFN-g and IL-17)<br>• T cell responses to antigens or peptides derived from islet antigens (including proinsulin, GAD, IA-2) measured by the level of IFNg, IL-17, IL-12 and IL-23 production in supernatants (Luminex)<br>• additional immunological biomarkers (e.g. flow cytometry profiles, T cell responsiveness measured by activation profiles, T reg assays, autoantibodies)
3. To investigate ustekinumab pharmacokinetics (PK) and compliance with therapy	Ustekinumab drug levels in serum at weeks 4, 12, 28 and 52
4. To explore association of C-peptide changes with age-appropriate PROMs.	C-peptide AUC and HYPOFEAR, DTSQ, and PedsQL questionnaires at weeks -2, 28 and 52
5. To compare participant and parent/carer proxy completed PROMs.	HYPOFEAR, DTSQ, and PedsQL questionnaires completed by participants and their parent/carer at weeks -2, 28 and 52
6. To investigate the longer term effect of ustekinumab on glycaemic control.	• Severe hypoglycaemic events<br>• Insulin use<br>• HbA1c<br>• C-peptide using DBS samples<br>• CGM data<br>At weeks 78 and 104

Table 1 – Objectives and outcome measures

Table key: -2 refers to the second screening visit about 2 weeks prior to dose 1.

Consent

Potential participants identified from health records, clinical contacts, patient registries and self-referrals through the T1DUK consortium and ADDRESS-2 website will be asked to view our short recruitment video (https://www.youtube.com/watch?v=8kuCefuBSW4) followed by a more detailed information sheet relevant to their age (see Appendices 1-4).

Written informed consent will be obtained for all participants at the first screening visit (see Appendices 5-8). For participants under 16, written assent will be obtained in addition to written consent from a parent/carer. Reconsent will be requested when participants turn 16.

Eligibility criteria

Consented participants will have eligibility checks (see Table 2), including auto-antibody screening and a MMTT. Tuberculosis must be ruled out using a chest x-ray and either a Mantoux test or a blood-based TB test. All blood and urine tests must be within clinically normal parameters.

For peer review only - http://bmjopen.bmj.com/site/about/guidelines.xhtml

The first dose of Investigational Medical Prduct (IMP) must be given within 100 days from clinical diagnosis. The screening MMTT must be within 37 days of the first dose of IMP.

INCLUSION CRITERIA	EXCLUSION CRITERIA
Clinical diagnosis of immune-mediated Type 1 diabetes mellitus as defined by the American Diabetes Association (ADA) (11, 12).	Breastfeeding, pregnancy or unwillingness to comply with contraceptive advice and regular pregnancy testing throughout the trial.
Commenced on insulin within 1 month of **clinical** diagnosis (defined as confirmed raised blood sugar (ADA criteria), not symptoms alone).	Prior exposure to ustekinumab within 3 months of the first dose of IMP.
An interval of ≤100 days between the **confirmed** diagnosis (defined as date of first insulin dose) and the first planned dose of the IMP.	Use of more than 10mg prednisolone daily (or equivalent) for >5 days within 3 months of the first dose of IMP.
Written and witnessed informed consent/assent to participate.	Prior exposure to any anti-lymphocyte monoclonal antibody, such as anti-CD20, anti-thymocyte globulin (ATG), Rituximab (Rituxan®), or Alemtuzumab (Campath®).
Male or female, aged 12-18 years inclusive at the time of randomisation.	Use of immunosuppressive or immunomodulatory therapies, including systemic steroids within 30 days prior to receiving the first dose and/or intent on using any monoclonal antibody therapy given for any indication for the duration (including follow up) of the trial.
Evidence of residual functioning beta-cells (peak serum C-peptide level > 0.2nmol/L in the MMTT test).	Use of any hypoglycaemia agents other than insulin, for more than 6 weeks, at any time prior to trial entry, including SGLT2 inhibitors.
Positive for at least one islet autoantibody (GAD, IA-2, ZnT8).	Use of inhaled insulin.
Body weight < 100kg.	Known alcohol abuse, drug abuse.
Willing to record all insulin doses and blood glucose levels required for monitoring during the study, including reporting any hypoglycaemic events.	Evidence of active Hepatitis B, Hepatitis C, HIV or considered by the investigator to be at high risk for HIV infection.
Willing to provide dried blood spot (DBS) samples.	Significant systemic infection during the 6 weeks before the first dose of the IMP (e.g. infection requiring hospitalisation, major surgery, requiring IV antibiotic treatment). Other infections e.g. glandular fever, bronchitis, sinusitis, cellulitis, or urinary tract infections must be assessed on a case-by-case basis by the investigator to assess whether they are serious enough to warrant exclusion or delay to inclusion.
Willing to wear the FreeStyle Libre Flash Glucose Monitor (FGM) device at least two weeks prior to a study visit.	History of current or past active tuberculosis (TB) infection and no latent tuberculosis. Active TB will be assessed using a **mandatory** chest x-ray and one of the following: a) blood-based test; b) the Mantoux skin test.
Willing to complete a diary and quality of life questionnaires.	Any live immunisations for 1 month prior to trial entry. Planned live immunisations are also not permitted during the study period.

For peer review only - http://bmjopen.bmj.com/site/about/guidelines.xhtml

Willing to consent to remote follow up via health records and telephone contact.	Previous use of any other investigational drug within the 3 months prior to the first dose and/or intent on using any investigational drug for the duration (including follow up) of the trial.
Female participants have a negative urine test for pregnancy; all participants must agree to use adequate contraception if they become/are sexually active (hormonal based contraception, double barrier contraception, abstinence) until 4 months following the date of their final treatment of IMP.	Recent (within 3 months) involvement in other research studies, which, in the opinion of investigators, may adversely affect the safety of the participants or the results of the study.
	Significantly abnormal laboratory results during the screening period, other than those due to T1D.
	Prior allergic reaction, including anaphylaxis, to any component of the IMP product.
	Prior allergic reaction, including anaphylaxis, to any human, humanised, chimeric or rodent antibody treatment.
	Any major planned surgery scheduled within the 30 day period prior to the first drug dose or anticipating requiring major surgery during the study period.
	Any other medical condition or treatment that, in the opinion of investigators, could affect the safety of the participant's participation or outcomes of the study, including malignancy, immunocompromised states and autoimmune conditions.
	Participants or parents/carers who lack the capacity to comply with trial requirements.

Table 2 – Trial eligibility criteria

Randomisation and blinding

Minimisation by age (12-15 vs 16-18 years respectively) and screened peak C-peptide levels (0.2 - 0.7 vs > 0.7 nmol/L) will be used to ensure balance between treatment groups. These variables are important prognostic factors and need to be evenly distributed between the groups. The baseline c-peptide cut off of 0.7nmol/L was selected to correspond with Lachin 2001. (13)

The treatment:placebo ratio will be 2:1 to promote recruitment and to provide additional data on drug safety. The minimisation algorithm and randomisation list will be provided by Sealed Envelope Ltd (https://sealedenvelope.com) and accessed by sites using an online randomisation system which was validated prior to use by statisticians in Swansea Trials Unit (STU). The system will email a randomisation code to designated site personnel including Pharmacy who will cross-reference it with a code break list to determine the allocation.

Dosage and regimen of placebo and ustekinumab will be matched. Only staff preparing the blinded syringe will be unblinded at sites. Participants, research staff and the trial office remain blinded, with only limited independent researchers at (STU managing the code break list and any IMP-related queries from Pharmacies.

Emergency unblinding will be managed by Sealed Envelope Ltd. If emergency unblinding is delayed, the treating clinician should treat the patient as if ustekinumab has been given.

Trial assessments

An overview of the trial procedures are listed in Table 3.

For peer review only - http://bmjopen.bmj.com/site/about/guidelines.xhtml

	Screening (SC) [1]		Dose							Follow up
			1	2	3	4	5	6	7	
**Visit**	**SC1**	**SC2**	1	2	3	4	5	6	7	8
**Week**	Approx. -2		0	4	12	20	28	36	44	52
**Window allowed**			≤100d of clinical diagnosis and ≤37d of SC2	+/-1 week						
Consent	X									
Medical History	X									
Physical exam	X		X		X		X			X
Concomitant medication (D)	X	X	X	X	X	X	X	X	X	X
Weight	X	X	X		X		X			X
Height	X		X		X		X			X
Vital signs	X	X	X		X		X			X
TB tests [2]	X									
Adverse events (including hypoglycaemia) (D)		X	X	X	X	X	X	X	X	X
Blood draw [3, 4]	X	X	X	X	X		X			X
Urine collection [5]	X	X	X	X	X	X	X	X	X	X
Dried blood spot review		X	X	X	X	X	X	X	X	X
Download blood glucose monitoring data			(X)	X	X	(X)	X	(X)	X	X
Glycaemic control (as part of routine care)			X	X	X	X	X	X	X	X
Insulin dose usage (D)		X	X	X	X	X	X	X	X	X
PROMs (adolescent & parent)		X					X			X

Table 3: Schedule of events at sites

Table key: (X) = optional data download; (D) = data from diary

[1] *Screening visits may be combined*

[2] *Chest x-ray AND either a i) blood test (T spot / quantiferon) or ii) Mantoux test*

[3] *Safety bloods - Full blood count; urea, electrolytes and creatinine; liver function tests (total bilirubin, total protein, albumin, AST (SGOT), SGPT (ALT), alkaline phosphatase; thyroid stimulating hormone; immunoglobulins (G, A, M); calcium; magnesium, phosphate, lipid profile (total cholesterol, LDL, HDL, triglyceride), HbA1c. For screening only, we also request HIV and Hepatitis B and C and TB testing.*

[4] *Bloods for Research laboratories:* ***Diabetes Research Unit Cymru (DRUC)*** *= MMTT, Islet autoantibodies, HbA1c, exocrine enzymes, proinsulin;* ***Kings College London (KCL)*** *= T cell assays, Flow-cytometry profiles of leucocyte populations, Cytokine production by CD4 and CD8 T cells;* ***Royal Devon & Exeter Hospital*** *= glucagon and somatostatin levels;* ***University of Bristol*** *= cell free DNA;* ***commercial company*** *= pharmacokinetics analysis.*

[5] *Urine samples are collected for pregnancy testing (females), Urinalysis for pH, blood and protein by dipstick urinalysis and laboratory analysis for albumin/creatinine ratio. We also collect a sample as part of the MMTT for DRUC.*

Further details are provided below for each assessment contributing towards the objectives.

Mixed-meal tolerance test (MMTT)

Secretion of C-peptide will be assessed for the primary outcome measure of the trial using a MMTT at screening and week 52. We also conduct an MMTT at week 28 to address a secondary objective.

For peer review only

For peer review only - http://bmjopen.bmj.com/site/about/guidelines.xhtml

Participants will fast from midnight and check their blood glucose on waking. The MMTT will be started between 7-11AM if their blood glucose prior to arriving is between 4.0 and 11.1mmol/L (inclusive). If it is <4mmol/L on waking, the test will be postponed to a different day. If the value is >11.1 mmol/L the participant will be advised to take an appropriate correction bolus of very short acting insulin at home so that the blood glucose would be within range on arrival at the hospital. The test may be postponed if the blood glucose is not in range after 2 hours.

The participant will be asked to drink a standardised liquid meal provided by the trial - Ensure Plus ™ 6 ml/kg (Maximum 360ml). This must be ingested within 5 minutes.

Blood glucose measurements will be taken prior to, and at the end of, the MMTT. The participant will void their bladder and urine will be collected at the end of the MMTT (at 120 minutes). Venous blood samples will be collected for measurement of C-peptide at time 0, 15, 30, 60, 90 and 120 minutes. Blood samples for mechanistic work will be taken at time 0.

Glucose monitoring

All participants will be provided with an Abbott FreeStyle Libre™ blood glucose monitoring system. Participants are expected to wear a sensor for at least 2 weeks prior to each study visit and will be advised to read their measurements at least 4-7 times a day. Anonymised data will be sent electronically to the Trial Office.

Hypoglycaemia

Participants will be advised by the research staff to record in a trial diary any hypoglycaemia symptoms between each study visit. This will be compared with glucose monitoring data. Participants will be asked to record a finger-prick blood glucose in the diary any time hypoglycaemic symptoms occur, even if the glucose monitor sensor is also being worn. A medic will categorise all hypoglycaemic events recorded in the diary according to ADA Guidelines.(14, 15)

Dried blood spot (DBS) measurements

DBS sampling will be carried out at home by the participant weekly from screening until week 28 and then monthly up to month 12 for the measurement of C-peptide. Blood samples will be obtained by finger prick and placed onto filter paper cards (Perkin Elmer). Samples will be provided before the first meal of the day, and one 60 minutes afterwards. Patients will be asked to withhold their pre-meal insulin until after the second DBS samples have been taken.

Insulin dose

Mean daily insulin use will be calculated over 7 consecutive days during the 2 weeks preceding all visits and participants will be asked to record all insulin usage in their diary during those 2 weeks. This value will be calculated in units of IU/kg/day. Where data from consecutive days are not available, the three days closest together will be used.

Body weight and BMI (clinical care measurement)

Body weight and height will be recorded at site visits and the most recent weight recorded will be used to calculate drug dosages for forthcoming treatment visits. Body mass index will be calculated as standard: weight (kg)/ [height (m)]^2.

Patient and parent reported outcome measures (PROMS)

Quality of Life for participants and their parent/carer will be assessed at screening, and weeks 28 and 52 by validated questionnaires: the Hypoglycaemia Fear Survey – HYPOFEAR;(16, 17) Diabetes Treatment Satisfaction Questionnaire for inpatients – DTSQ;(18) Paediatric Quality of Life inventory – PedsQL ™ Copyright 1998 JW Varni, PhD (generic core scale (19, 20) and diabetes-specific (21, 22) modules).

The questionnaires will be completed during the latter stages of the MMTT whilst the participant and parent are waiting for the end of the test. Participant and parent will be encouraged not to discuss their responses with each other.

For peer review only - http://bmjopen.bmj.com/site/about/guidelines.xhtml

Glycaemic control

Glycaemic control will be maintained according to clinical guidelines with the support of the participant's local diabetes clinical care team. HbA1c will be measured as per the study schedule based on the local laboratory results with a target value set according to 2015 NICE guidelines (23) in agreement with the participant and their clinical care team. Where this target is not met, advice will be given as clinically required.

Urine C-peptide/creatinine ratio (UCPCR)

Urine C-peptide/creatinine ratio will be measured from the 120 minute urine sample taken during the MMTT at screening, weeks 28 and 52. We selected this to determine whether it could be used as an alternative non-invasive test for future trials based on successes in other trials. (24, 25)

HbA1c

HbA1c will be tested in the local NHS laboratories of the study sites to guide clinical care. A blood sample will also be taken at weeks 0, 12, 28 and 52 for measurement of HbA1c using an HPLC method.

Immunological changes (mechanistic study)

Changes in immune mechanistic parameters including IL-17 and IFN-gamma production, phenotypes and function of CD4+ and CD8+ T cells will be assessed by flow cytometry immunophenotyping, Fluorospot and other immune assays, such as Luminex, at screening or week 0 (as baseline), and week 12, 28 and 52, using primarily overnight blood samples and also cryopreserved peripheral blood mononuclear cells (PBMC).

Changes in IL-17 and IFN-gamma production will be measured in both agnostic and antigen-specific manner, where for the latter T cell responses will be determined in response to antigens or peptides derived from islet antigens.

Long term follow-up assessments

We will record weight and height, insulin doses over a two week period, severe hypoglycaemia events and HbA1c levels at time points closest to weeks 78 and 104 which also coincide with a routine clinic visit. The data will be sourced from the medical records where possible and from the participant using a short questionnaire.

We also seek consent to have two additional DBS cards completed at the corresponding time points and an anonymised copy of the glucose monitor data for the two weeks prior to the time points matching the clinic visits.

TRIAL TREATMENTS

Ustekinumab (Stelara®)

Ustekinumab is a fully human IgG1k monoclonal antibody (mAb) supplied by the marketing authorisation holder Janssen-Cilag Ltd (EU/1/08/494/002). It is supplied as sterile single use 2ml glass vials containing 0.5 ml of solution with 45mg of ustekinumab for injection. Section 4.8 of the Summary of Product Characteristics (SmPC) for STELARA® (https://www.medicines.org.uk/emc/product/4413/smpc) dated 22 March 2018 will be used as the Reference Safety Information (RSI) for pharmacovigilance purposes. It was assessed by the MHRA as part of the original approvals process.

The SmPC has been updated three times so far. However, there were no significant change to the safety parameters of the trial so the original version continues to be used.

For peer review only - http://bmjopen.bmj.com/site/about/guidelines.xhtml
For peer review only

Placebo

Saline in the form of Sodium Chloride 0.9% w/v solution for injection will be used as the placebo. Any brand of saline with a marketing authorisation in the UK can be used for this trial. A representative SmPC will be used to represent all saline (marketing authorisation number PL 02848/0157).

Discontinuation / modification of drug dosing

Drug dosing will only be altered in response to a change in body weight as per the protocol which states 2mg/Kg ≤ 40Kg or 90mg if >40Kg.

WITHDRAWALS

The Principal Investigator or participant (or parent/carer if the participant is <16y) can opt to discontinue treatment for any reason. The participant (and parent if <16y) will be asked to remain in the trial for sample and data collection purposes only. They have the right to withdraw completely without giving a reason.

Oversight committees and the Sponsor can request the withdrawal of a participant(s) or to terminate the trial.

Exceeding the timeframe for receiving medication may also result in withdrawal from treatment.

SAFETY REPORTING

The risk of major adverse unexpected events is anticipated to be low. Ustekinumab has a marketing authorisation in the age group being studied for other indications. The available SmPC describes all essential information for the use of the medicine, and the qualitative and quantitative information on benefits and risks. Participants being exposed to ustekinumab are a different disease population from those described in the SmPC. In addition, the dose used in this trial is higher than that currently licensed for psoriasis in adolescents, although it (and higher doses) have been used in adults with both psoriasis and Crohn's disease.

Hypoglycaemic events are common in this population and may not necessarily be IMP-related. Hypoglycaemia rates are an important secondary outcome, as it is anticipated that these should be reduced by ustekinumab if it is effective. Hypoglycaemic events are recorded specifically for this trial separately from other adverse events (AEs) because they require medical assessment according to ADA Guidelines (14, 15).

A review of AEs will be performed at all visits (participant-reported) and using blood and urine samples at screening and 0, 12, 28 and 52 weeks. A urine pregnancy test will be completed on all females at all trial visits. Principal Investigators will be expected to assess any values outside the laboratory reference range for clinical significance.

Hypoglycaemia and diabetic ketoacidosis are considered expected for newly diagnosed T1D patients. If the event leads to death, this will be considered unexpected.

Any pregnancies for female participants or the pregnant partners of male participants must be reported immediately. Pregnant participants will be withdrawn from treatment and asked to provide consent to follow up the pregnancy until the child is 12 months old.

POST TRIAL CARE

Following completion of their trial participation, participants will be kept informed of ongoing trial developments including final outcomes following statistical analyses. Should participants be concerned about implications arising from their trial participation, they will be asked to discuss these

For peer review only - http://bmjopen.bmj.com/site/about/guidelines.xhtml

with their local clinicians. Senior members of the trial team will be available for further advice should the local clinician require.

Once the trial is complete (defined as last participant, completing the 24 month follow up data collection task), following unblinding, individual participants and their local clinicians will be informed by letter on request as to which arm of the trial they were randomised to. After completing the first 52 weeks of the trial, clinical care and follow-up will be provided by the participant's local diabetes care team. Ustekinumab will not be available for ongoing therapy.

STATISTICS AND DATA ANALYSIS

Sample size considerations

The power calculation closely follows Lachin (13) based on data for children and young adolescents aged 13-17 years as well as the T1DAL study in 12-35y olds (26). A sample size of 66 apportioned in a 2:1 ratio has a greater than 85% power to detect a 0.2nmol/L difference between the 2-hour MMTT mean AUC C-peptide values of the intervention and placebo arms which are assumed to be 0.5 and 0.3 (nmol/L) respectively at 12 months. Seventy-two participants (48 ustekinumab :24 placebo) will be recruited to allow for approximately 10% loss to follow-up.

Data analysis

Data cleaning and preparation processes will be carried out prior to final analysis. A statistical analysis plan (SAP) approved by the Data Safety Monitoring Board (DSMB) will be followed.

All participants enrolled will be followed up and included unless they withdraw from the study before the administration of the first dose. An intention to treat (ITT) analysis will be carried out. Per protocol analysis of the primary outcome will also be carried out alongside the ITT analysis if deemed necessary by the Trial Steering Committee (TSC).

The primary data analysis will be the application of analysis of covariance to the 12-month recorded AUC mean values of C-peptide taking into account the baseline values of these measures and using transformations as suggested by Lachin (13). The analysis will be adjusted by important covariates such as gender, age at recruitment, baseline insulin use and glycaemic control.

For the secondary outcomes including the mechanistic and questionnaire studies we will evaluate the various outcomes using the most appropriate statistical approach i.e. binomial or logistic regression for binary outcomes, Poisson or related count outcome models for number of events/objects and linear models for continuous outcomes. Where necessary, mixed or multilevel models will be used to account for correlation within observations.

No interim analysis is planned. No subgroup analysis is planned. Should there be substantial non-fidelity to allocated treatment, a per-protocol analysis for the primary outcome will be considered after approval by the TSC.

Efficacy analyses will be adjusted by gender, age and baseline test values. Safety analysis will not be adjusted.

Interim analysis on safety data only will be conducted if requested by TSC/ DSMB. Decision criteria based on safety as part of a guideline for early stopping or other adaptations will be set by TSC with input from DSMB.

Every attempt will be made to minimise missing data, encouraging participants to provide week 52 data even if they are no longer taking the interventional medication. Patterns and level of missing data will be examined. Multiple imputation will be considered if required, if there are more than 5% and less than 10% (>3 and <7 participant) missing.

For peer review only - http://bmjopen.bmj.com/site/about/guidelines.xhtml

DATA MANAGEMENT

Source documents produced for this trial will be filed with the participant's medical records. Source data will be entered into trial-specific database of electronic Case Report Forms (eCRFs) at the end of each trial visit within a site agreed timespan. These eCRFs will be coded with the participants study number and will not include patients' names and addresses and will conform to General Data Protection Requirements (GDPR). This database (MACRO v4.7 Elsevier Ltd 2017) will be hosted on a Swansea University server with back up and restoration procedures in place. All paper CRFs can be found by logging into the trial website and entering the password.

The trial database will be managed and operated as required by GCP. The site investigator or delegate will record all study data using the trial specific electronic database provided by STU. All data will be handled and stored in accordance with GDPR, Data Protection Act and applicable legislation.

Data will be checked according to the trial Data Management Plan and queries will be generated and sent to the site investigator for response using the database.

Data from laboratories and the anonymised glucose monitoring and diary data from patients will be securely transferred to the Trial Office.

Remote data collection after week 52 will be done using the REDCap™ database with links to participant questionnaires emailed by site researchers. No identifiable data is collected in the database during remote follow up.

The CI and trial statistician will have access to the final dataset for analysis. Should PIs or others require access to the final dataset this will require approval by the TMG, TSC and Sponsor.

The trial data will be held in a data repository, the location of which is still being negotiated.

MONITORING

Monitoring of this trial to ensure compliance with Good Clinical Practice (GCP) and scientific integrity will be conducted by STU via central and on-site monitoring as per the Trial Monitoring Plan.

This will include 100% central monitoring of all primary outcome data, with site initiation and closedown visits for all sites, and a minimum of one monitoring visit during the recruitment period to complete 100% Source Data Verification on primary outcome data. In addition, the trial office will facilitate monitoring by local R&D departments at any of the trial sites, should this be requested.

DISSEMINATION

A Publication Plan will be developed to organise the outputs from this trial. Outputs will be disseminated using highly accessed, peer-reviewed medical journals and will be presented at conferences.

Authorship will be agreed upon by the CI, PIs, and members of the TMG and will follow the guidance provided by the International Committee of Medical Journal Editors.

PATIENT AND PUBLIC INVOLVEMENT

We recruited a panel of children with T1D to help us develop a short recruitment video.

We recruited six Patient and Public Involvement (PPI) representatives, two for each committee (TMG, DSMB and TSC). All PPI representatives are either parents of children with T1D or have T1D themselves. Our PPI representatives assist in the development of participant facing documentation, support applications for approvals and will review and help to disseminate our results

For peer review only - http://bmjopen.bmj.com/site/about/guidelines.xhtml

STUDY MANAGEMENT

The Trial Office is based at STU, with the Chief Investigator, paediatric lead and adult lead all working at Cardiff University.

The sponsor of the trial is Cardiff University. The sponsor can be contacted at resgov@cardiff.ac.uk.

The sponsor has arranged appropriate insurance and indemnity to meet the potential legal liability for harm to the participants arising from the design or management of the trial for negligent harm. In addition, the trial health professionals hold substantive or honorary NHS contracts, giving them the protection of the appropriate NHS clinical negligence arrangements

TRIAL COMMITTEES

The trial oversight committees are the DSMB and TSC who will meet biannually. They comprise of clinical experts, a statistician and public and patient involvement (PPI) representatives and each work to a pre-agreed charter. The DSMB to provide ethical and safety reviews (including the assessment of adverse events and protocol deviations) and the TSC will have general oversight of the trial to ensure recruitment, treatment and follow up visits are safe and providing the relevant data, and that the protocol is being adhered to, based on DSMB recommendations.

The TMG consists of the trial team, independent advisors and PPI representatives and meets at least quarterly and provides a forum to discuss trial progress with key members and the content of reports to, and responses from, the oversight committees.

REGULATORY APPROVALS

Ethical approval for the trial protocol was received on 18/09/18 from Wales REC 3 – reference 18/WA/0092. Regulatory approval from the Medicines and Healthcare products Regulatory Agency (MHRA) was received on 26/06/18. Site-specific capability and capacity will be sought for the trial. Amendments to REC-approved documentation will not used until approval from the relevant regulatory authorities is in place.

AUTHORS' CONTRIBUTIONS

Authors MA, NB, JBM, AB, WYC, CD, GD, GF, JG, SH, GH, HH, SL, SMJ, AM, RS, DT, TT, KC and JY played a significant role in the development of the protocol. Authors SMJ and AB are our PPI representatives and review the protocol and other trial related documentation. CD is the Chief Investigator whilst JG is the paediatric T1D lead and DT is the adult T1D lead. CD and DT are the joint senior authors of the paper.

FUNDING STATEMENT

This project (project reference 16/36/01) is funded by the Efficacy and Mechanism Evaluation (EME) Programme, an MRC and NIHR partnership. The views expressed in this publication are those of the author(s) and not necessarily those of the MRC, NIHR or the Department of Health and Social Care. Additional funding for mechanistic laboratory tests has been provided by JDRF (Juvenile Diabetes Research Foundation) International Award 3-SRA-2018-629-S-B.

COMPETING INTERESTS STATEMENT

At the time of writing, no financial conflict or any other relevant connection or shared interest was declared for the CI, committee members or anyone involved in the management of the trial.

For peer review only - http://bmjopen.bmj.com/site/about/guidelines.xhtml

Janssen-Cilag will supply ustekinumab for the trial at no cost, while Abbott Diagnostics will supply glucose monitoring devices. Neither have been granted, nor sought to obtain, any financial or in kind advantage or reward for supplying the products. However, glucose sensors will be purchased from Abbott at cost price for the duration of the trial. Both parties will be contracted by the sponsor.

License statement

I, the Submitting Author has the right to grant and does grant on behalf of all authors of the Work (as defined in the below author licence), an exclusive licence and/or a non-exclusive licence for contributions from authors who are: i) UK Crown employees; ii) where BMJ has agreed a CC-BY licence shall apply, and/or iii) in accordance with the terms applicable for US Federal Government officers or employees acting as part of their official duties; on a worldwide, perpetual, irrevocable, royalty-free basis to BMJ Publishing Group Ltd ("BMJ") its licensees and where the relevant Journal is co-owned by BMJ to the co-owners of the Journal, to publish the Work in BMJ Open and any other BMJ products and to exploit all rights, as set out in our licence.

For peer review only

For peer review only - http://bmjopen.bmj.com/site/about/guidelines.xhtml

Figure 1: Trial flowchart

For peer review only

For peer review only - http://bmjopen.bmj.com/site/about/guidelines.xhtml

REFERENCES

1. Wood JR, Miller KM, Maahs DM, Beck RW, DiMeglio LA, Libman IM, et al. Most youth with type 1 diabetes in the T1D Exchange Clinic Registry do not meet American Diabetes Association or International Society for Pediatric and Adolescent Diabetes clinical guidelines. Diabetes Care. 2013;36(7):2035-7.

2. Centers for Disease Control and Prevention. Diabetes death rates among youths aged ≤ 19 years--United States, 1968-2009. MMWR Morb Mortal Wkly Rep. 2012;61(43):869-72.

3. Wilcox NS, Rui J, Hebrok M, Herold KC. Life and death of β cells in Type 1 diabetes: A comprehensive review. J Autoimmun. 2016;71:51-8.

4. Lachin JM, McGee P, Palmer JP, Group DER. Impact of C-peptide preservation on metabolic and clinical outcomes in the Diabetes Control and Complications Trial. Diabetes. 2014;63(2):739-48.

5. Sorensen JS, Johannesen J, Pociot F, Kristensen K, Thomsen J, Hertel NT, et al. Residual beta-Cell function 3-6 years after onset of type 1 diabetes reduces risk of severe hypoglycemia in children and adolescents. Diabetes care. 2013;36(11):3454-9.

6. Herold KC, Gitelman SE, Ehlers MR, Gottlieb PA, Greenbaum CJ, Hagopian W, et al. Teplizumab (anti-CD3 mAb) treatment preserves C-peptide responses in patients with new-onset type 1 diabetes in a randomized controlled trial: metabolic and immunologic features at baseline identify a subgroup of responders. Diabetes. 2013;62(11):3766-74.

7. Pescovitz MD, Greenbaum CJ, Bundy B, Becker DJ, Gitelman SE, Goland R, et al. B-lymphocyte depletion with rituximab and β-cell function: two-year results. Diabetes Care. 2014;37(2):453-9.

8. Quattrin T, Haller MJ, Steck AK, Felner EI, Li Y, Xia Y, et al. Golimumab and Beta-Cell Function in Youth with New-Onset Type 1 Diabetes. N Engl J Med. 2020;383(21):2007-17.

9. Marwaha AK, Tan S, Dutz JP. Targeting the IL-17/IFN-γ axis as a potential new clinical therapy for type 1 diabetes. Clin Immunol. 2014;154(1):84-9.

10. Greenbaum CJ, Schatz DA, Haller MJ, Sanda S. Through the fog: recent clinical trials to preserve β-cell function in type 1 diabetes. Diabetes. 2012;61(6):1323-30.

11. American Diabetes A. Standards of medical care in diabetes--2014. Diabetes care. 2014;37 Suppl 1:S14-80.

12. American Diabetes A. 2. Classification and Diagnosis of Diabetes: Standards of Medical Care in Diabetes-2018. Diabetes care. 2018;41(Suppl 1):S13-S27.

13. Lachin JM, McGee PL, Greenbaum CJ, Palmer J, Pescovitz MD, Gottlieb P, et al. Sample size requirements for studies of treatment effects on beta-cell function in newly diagnosed type 1 diabetes. PLoS One. 2011;6(11):e26471.

14. Seaquist ER, Anderson J, Childs B, Cryer P, Dagogo-Jack S, Fish L, et al. Hypoglycemia and diabetes: a report of a workgroup of the American Diabetes Association and the Endocrine Society. Diabetes Care. 2013;36(5):1384-95.

15. Workgroup on Hypoglycemia AeDA. Defining and reporting hypoglycemia in diabetes: a report from the American Diabetes Association Workgroup on Hypoglycemia. Diabetes Care. 2005;28(5):1245-9.

16. Gonder-Frederick L, Nyer M, Shepard JA, Vajda K, Clarke W. Assessing fear of hypoglycemia in children with Type 1 diabetes

and their parents. Diabetes Management. 2011;1(6):627-39.

17. Gonder-Frederick L, Schmidt KM, Vajda KA, Greear ML, Singh H, Shepard JA, et al. Psychometric Properties of the

Hypoglycemia Fear Survey-II for

Adults With Type 1 Diabetes. Diabetes Care. 2011;34:801-6.

18. Bradley C. The Diabetes Treatment Satisfaction Questionnaire: DTSQ. Handbook of Psychology and Diabetes: a guide to psychological

measurement in diabetes research and practice. Switzerland: Harwood Academic Publishers; 1994.

For peer review only - http://bmjopen.bmj.com/site/about/guidelines.xhtml

19. Varni JW, Seid M, Rode CA. The PedsQL: measurement model for the pediatric quality of life inventory. Med Care. 1999;37(2):126-39.

20. Varni JW, Seid M, Kurtin PS. PedsQL 4.0: reliability and validity of the Pediatric Quality of Life Inventory version 4.0 generic core scales in healthy and patient populations. Med Care. 2001;39(8):800-12.

21. Varni JW, Delamater AM, Hood KK, Raymond JK, Chang NT, Driscoll KA, et al. PedsQL 3.2 Diabetes Module for Children, Adolescents, and Young Adults: Reliability and Validity in Type 1 Diabetes. Diabetes Care. 2018;41(10):2064-71.

22. Varni JW, Delamater AM, Hood KK, Raymond JK, Chang NT, Driscoll KA, et al. Pediatric Quality of Life Inventory (PedsQL) 3.2 Diabetes Module for youth with Type 2 diabetes: reliability and validity. Diabet Med. 2019;36(4):465-72.

23. Excellence NIfHaC. Diabetes (type 1 and type 2)

in children and young people:

diagnosis and management. In: Excellence NIfHaC, editor. www.nice.org.uk/guidance/ng182015.

24. Tatovic D, Luzio S, Dunseath G, Liu Y, Alhadj Ali M, Peakman M, et al. Stimulated urine C-peptide creatinine ratio vs serum C-peptide level for monitoring of β-cell function in the first year after diagnosis of Type 1 diabetes. Diabet Med. 2016;33(11):1564-8.

25. Besser RE, Ludvigsson J, Jones AG, McDonald TJ, Shields BM, Knight BA, et al. Urine C-peptide creatinine ratio is a noninvasive alternative to the mixed-meal tolerance test in children and adults with type 1 diabetes. Diabetes Care. 2011;34(3):607-9.

26. Rigby MR, DiMeglio LA, Rendell MS, Felner EI, Dostou JM, Gitelman SE, et al. Targeting of memory T cells with alefacept in new-onset type 1 diabetes (T1DAL study): 12 month results of a randomised, double-blind, placebo-controlled phase 2 trial. Lancet Diabetes Endocrinol. 2013;1(4):284-94.

For peer review only

For peer review only - http://bmjopen.bmj.com/site/about/guidelines.xhtml

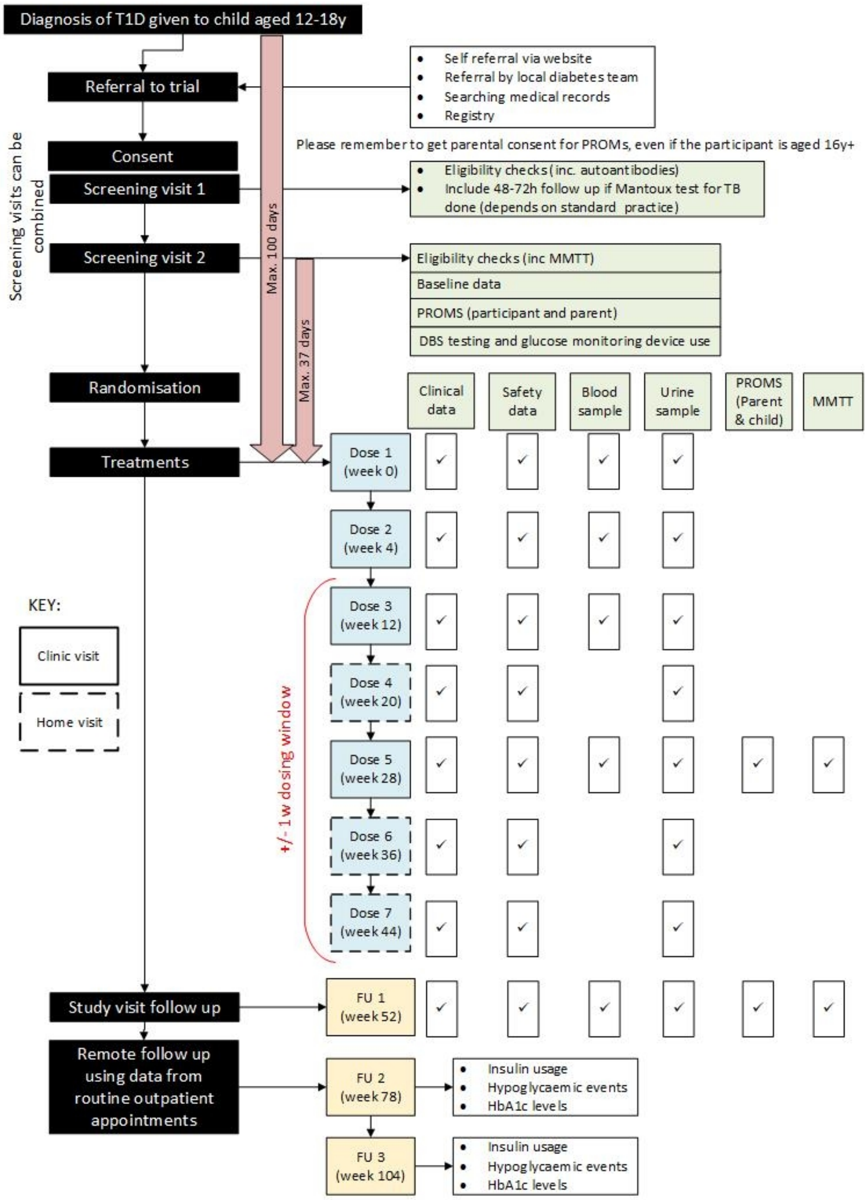

Figure 1: Trial Flowchart

197x273mm (96 x 96 DPI)

[Insert local headers]

A research study to see if the medicine Ustekinumab can make diabetes easier to manage

CONTACT DETAILS FOR STUDY TEAM:

NURSE:

DOCTOR:

EMERGENCIES:

FOR YOUNG PEOPLE AGED 12-15 YEARS OLD

We would like you to help us with our research study. Please read this information carefully and talk to your parent or carer about the study. Ask us if there is anything that is not clear or if you want to know more. Take time to decide if you want to take part. It is up to you if you want to do this. If you decide not to take part, then that is fine, you will be looked after by your doctors just the same.

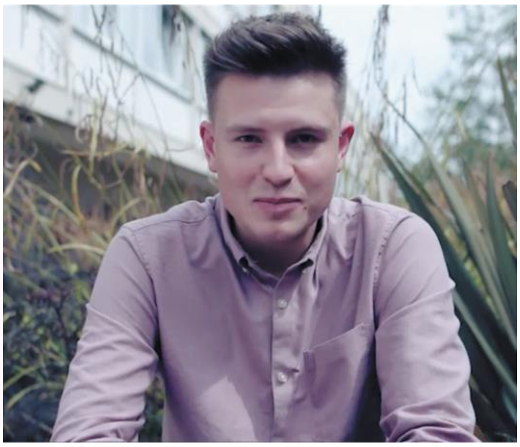

Please look at our video explaining the trial at www.type1diabetesresearch.org.uk/current-trials. The blue box below and the video contains the key points about the study. If you would like to know more, please read the rest of this leaflet.

KEY POINTS ABOUT THE STUDY:

- We want to see if the study medicine, **Ustekinumab**, can help people with Type 1 Diabetes (the type you have). The medicine works by "protecting" some of the cells in the body that still produce insulin to help make diabetes easier to manage.
- The study will involve injections of the study medicine or a placebo (a "dummy" medicine that has no effects) every 1-3 months. These injections are given under the skin like insulin injections and will be done by the study doctor or nurse. Neither you nor the research team will know if you are receiving the study medicine or the placebo.
- The medicine is already being used to treat other illnesses quite safely.
- You will be asked to come into your local hospital or research centre for 10 study visits over a 15 month period, but where possible this will be on the same day as your routine hospital visits and 3 of the visits can sometimes be done by a research nurse at your home.
- We will ask you to provide extra blood and urine (wee) samples to check that it is OK for you to take part in the study and to check your health during the study. We will also use them to do some special tests in laboratories.
- You will receive a small gift voucher for each study visit you come to.
- You will wear a flash glucose monitor ("Freestyle Libre") on your arm to check your blood glucose levels for 2 weeks before each study visit. You can keep this for use at home for the rest of the time you are in the study if you want to.
- You can stop taking part in the study at any time and do not have to give a reason why.

For peer review only - http://bmjopen.bmj.com/site/about/guidelines.xhtml

WHY IS THIS STUDY BEING DONE?

This study is being done to see if a medicine called **Ustekinumab** can help to "protect" the cells in the body that produce insulin in young people recently diagnosed with Type 1 Diabetes (or T1D). At the time of diagnosis, most people your age with diabetes have some of their insulin-producing cells still working. It usually takes between 1 and 5 years before they stop working completely due to damage by the immune system. Sometimes these last few working cells can make enough insulin to make blood glucose levels stable and easier to control – this is called the "Honeymoon period". This period is only temporary and doesn't last. Ustekinumab, the study medicine, may make this period last longer by reducing the effects of the immune system on the insulin-producing cells in the pancreas.

WHY HAVE I BEEN ASKED TO TAKE PART?

You have been chosen because you are aged 12 - 18 years old and have recently been diagnosed with T1D.

DO I HAVE TO TAKE PART?

No. It is completely up to you whether or not you take part and you can always change your mind at any time. If you decide you don't want to take part, that's OK. Nobody will be upset and the medical care you get from your diabetes health care team will not be affected. You do not need to decide if you want to take part straight away. You can take your time and talk about the study with your family, friends and the study team if you want to. You need to let us know within 6 weeks of being diagnosed with diabetes, so that we can start the treatment early enough.

If you are interested in this study then you and your parent/carer should let your doctor or nurse know. Someone from the research team will get in touch to explain more about the study and answer any questions you have. You will be asked to sign a form to say that you are happy to take part. Your parent/carer will also sign a form to say that they agree too.

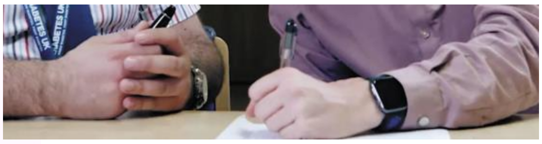

WHAT HAPPENS ONCE I HAVE AGREED TO TAKE PART?

SCREENING VISITS

Once the forms have been signed by you and your parent/carer we will do checks - "screening" - to see if you are suitable for the study. There are 2 screening visits during which we will do some tests to tell us about your health and your diabetes.

The first screening visit involves:

- A general health check by a doctor to make sure you can take part.
- We will take some blood samples (this will range from half a tablespoon to two tablespoons, depending on how much the hospital laboratory needs) from your arm.
- We will check whether you have an infection called TB (tuberculosis) or any viral infections which would stop you from taking part.
- We will also ask you to wee into a container so that we have a urine sample to test your kidneys and for girls, to make sure you're not pregnant.

The second screening visit involves:

- A **mixed meal tolerance test** (the Milkshake test) to test how much insulin your body still makes (see www.type1diabetesresearch.org.uk/current-trials). This involves coming to the hospital in the morning having not had breakfast. A small thin plastic tube will be put into a vein in your arm to take blood samples (with local anaesthetic ("numbing") cream/spray if you want it). This means that you will not need to have a needle in your arm for every blood sample we take during the test. We will need to take almost 3 tablespoons of blood at this visit in total. You then drink a flavoured drink that makes your body release insulin. Blood samples will be taken regularly through the tube over a period of 2 hours and your blood glucose is checked. During this time you can rest on a bed and can play on the internet or do other activities. At the end, the tube is removed. You then take an insulin injection depending on your blood glucose level and can leave the hospital when ready.

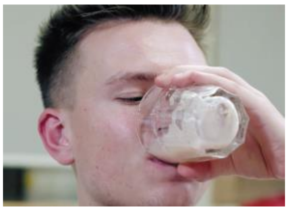

- We will ask you to complete a short questionnaire about your diabetes and how you feel. Your parent/carer will complete a similar one to you. You can ask the study nurse if you are unsure how to answer any questions.
- We will give you a free Freestyle Libre blood glucose monitor to wear at least two weeks before every visit (you can keep it on all the time if you want to).

If one of the screening tests tells us that you cannot take part, we will let you know as soon as possible.

If you and your parent/carer decide that it is easier to do both screening visits in one go, we can do this.

If the results of the screening tests are OK, then a computer will decide by chance whether you will receive the study medicine (Ustekinumab) or the placebo ("dummy" medicine).

Two thirds of young people taking part will receive the study medicine, and one third the placebo.

For peer review only - http://bmjopen.bmj.com/site/about/guidelines.xhtml

Neither, your family or the doctors and nurses will know which treatment you received until the end of the study.

STUDY VISITS

Once you enter the main study, you will be asked to come to 8 study visits over 52 weeks. Some of these visits may be on the same day as a routine hospital visit and 3 of the visits may be done by a nurse visiting you at home.

Activities at the visits may include:

- Check up by a nurse or doctor
- Urine sample tests
- Blood sample tests (between 3 and 4 tablespoons). If you feel unwell while this is being done, or you do not want to give the whole amount, the doctor or nurse can stop taking blood at any time.
- An injection of either the study medicine or the placebo. You will be asked to stay in the hospital for 1 hour after you receive the first injection so that the study team can make sure that you are OK.
- We will also download data that has been collected by a blood glucose monitoring device we will give you.
- Two study visits will involve mixed meal tolerance (Milkshake) tests.

FOLLOW UP

After the final study visit at week 52, we will check your hospital records for the next 12 months to find out how you are doing. You do not need to come into the hospital for a study visit. We may need to call you or your parent/carer up at home to check that you are OK and in good health.

WHAT ELSE WILL I BE ASKED TO DO?

You will be asked to complete a diary to record:

a) How much insulin you take in the two weeks before each study visit.
b) If you feel unwell or have to take any medicines during the study.
c) If you have any hypoglycaemic reactions (low blood glucose levels) that make you feel unwell during the study.

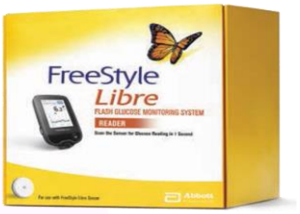

- We will ask you to test your blood glucose levels at home for at least 2 weeks before each visit using our FREE Abbott Freestyle Libre flash glucose monitoring system so you don't need to do extra finger prick tests for the trial. You will need to wear a sensor on your arm for the two weeks before the study visit if you want to take part and you can wear it every day if you want to. We will give you enough sensors to allow this for a year. We will also show you how it works.
- We will ask you to give a finger prick blood spot sample (just like a normal finger prick glucose test) before and 1 hour after the first meal of the day. This should be done once a week up for the first 28 weeks, then once a month for the next 6 months. You should do these at home with help from your parent/carer. We will show you how to do this - you prick your finger and then drop a spot of blood onto a special card.
- You will be asked to complete 3 questionnaires about your diabetes and how you are feeling at the start, middle and end of the study. Your parent/carer will also complete similar questionnaires for us.
- You will be asked not to have certain vaccinations before, during and immediately after the study.
- We will ask for permission to see your hospital records for a year after you finish your study visits to look at any changes to your insulin doses and glucose levels after stopping the treatment.
- A urine pregnancy test will be routinely done for all girls at each study visit.
- You need to be careful that you do not end up getting pregnant or making someone pregnant while you are in the study. We are asking everyone taking part to agree to use contraceptives before we can consider them for the study. Your parents/carers will be told about this requirement. Your GP or a pharmacist can advise on contraception if you don't want to discuss this with your parents/carers.

WILL THE STUDY HELP ME?

If you are in the group that receives the study medicine, it is possible that it will help your pancreas make insulin for longer. However, we cannot say this for certain until we have completed this study. During the study your diabetes will be very closely monitored. This will include regular check-ups with your local diabetes team including routine blood testing. You will have more time with the research team to discuss your diabetes and ask questions than at a normal clinic appointment.

You will be given an Abbott Freestyle Libre blood glucose monitoring system that you can use for the whole year to monitor your blood glucose levels without extra finger prick tests. You must wear this for the two weeks before the study visit if you want to take part. The treatments will stop at week 44 and you will not receive any further injections of the study drug / placebo during the trial.

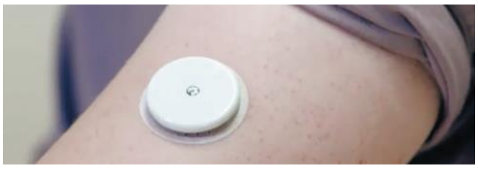

For peer review only - http://bmjopen.bmj.com/site/about/guidelines.xhtml

WHAT IF I DO NOT WANT TO TAKE PART ANYMORE?

Just tell your parents/carers to let us know. Nobody will be cross with you. You will still receive the same care from your doctors. We will still test the blood samples you have given so far unless you ask us not to.

WHAT IF THERE IS A PROBLEM OR SOMETHING GOES WRONG?

Tell your parents/carers as soon as possible if there is a problem so that they can let the study doctor or research team know and they will try to sort it out straight away. We will tell your parent/carer more about what to do if there is a problem when we talk to them about the study.

WHAT ARE THE POSSIBLE SIDE EFFECTS FOR ME IN PARTICIPATING IN THE STUDY?

You may get a bruise or a little discomfort where the needle goes in for the blood tests. We can use a cream or spray to numb the area from where we take blood if you like.

The FreeStyle Libre sensor may cause a slight rash for some people who might be allergic to the sticky part of the sensor. Please let the research team know if this happens.

During the Milkshake test, your blood glucose levels may be higher than usual because you will not have taken insulin immediately beforehand. The nurses and doctors will be available to help you make any changes to your usual insulin doses after this test.

The study medicine has not been tested in people with Type 1 Diabetes before so there may be some effects that we do not yet know about. Because the medicine acts on the immune system, there is a possibility that it could increase the risk of infections and cancer, but so far this has not been the case in people treated with this medicine for others diseases. It is also possible that you may get an allergic reaction to the treatment injection. We will ask you to stay for one hour after your first injection to check for any reactions.

We will need to do a chest x-ray. X-rays can cause damage to the body when you are older but we are only asking for one, which will hardly affect you.

If you feel ill at any time during the trial and go to your GP or the hospital, please show them the membership card you will be given so that they can contact the research team to ask about possible side effects.

WILL I RECEIVE ANY PAYMENT FOR TAKING PART?

As a thank you for you helping us with our research you will receive a £10 gift voucher for each treatment visit you come to and we'll give you a £30 gift voucher if you come to the final visit (visit 8) (that's £100 in total if you come to all visits). We will pay your parent/carer for any travel costs for attending study visits.

WHAT INFORMATION WILL YOU COLLECT AND HOW WILL IT BE KEPT PRIVATE?

We will ask for your name and contact details as well as information about your health which we get from your tests, your blood glucose monitor and your medical records. We will also ask you to complete a questionnaire at three time points. All of this information will be kept safely and nobody else will know that it is about you because your name will be removed and replaced with a study number. The people in our research team and diabetes care staff at the local hospital or research centre will know that you are taking part. We will also tell your family doctor (GP) that you are in the study if your parent/carer agrees to this.

People at the research laboratories will not know who you are when they test your samples. Your sample will be given a study number to replace your name so any study samples and data related to you will be anonymised. Cardiff University is responsible for the data we collect about you and will keep it secure for as long as is necessary.

WHAT WILL HAPPEN TO ANY SAMPLES I GIVE?

We want to test your blood and urine to better understand your diabetes and how the study medicine affects your diabetes. Samples will be tested in different research laboratories in the UK and may also be sent to special laboratories in Europe, America or Canada. These samples will not have your name on, only a study number so your identity will not be revealed.

We would like to keep any leftover blood samples in storage permanently for future research - we will ask you and your parent/carer to decide if they want you to do this.

WHAT SHOULD I DO NOW?

If you are interested in taking part, let your parents/carers know and they will get in touch with the study nurse/doctor.

Thank you for taking the time to read this information sheet and for considering taking part in this research study

For peer review only - http://bmjopen.bmj.com/site/about/guidelines.xhtml

[Insert local headers]

USTEKID

NHS National Institute for Health Research

CARDIFF UNIVERSITY PRIFYSGOL CAERDYDD

Swansea University Prifysgol Abertawe Swansea Trials Unit Uned Dreialon Abertawe

A research study to see if the medicine Ustekinumab can make diabetes easier to manage

CONTACT DETAILS FOR STUDY TEAM:

NURSE:

DOCTOR:

EMERGENCIES:

FOR PARENTS OF YOUNG PEOPLE AGED 12-15 YEARS OLD

We would like you to help us with our research study. Please read this information carefully and talk to your parent or carer about the study. Ask us if there is anything that is not clear or if you want to know more. Take time to decide if you want to take part. It is up to you if you want to do this. If you do not, then that is fine, you will be looked after by your doctors just the same.

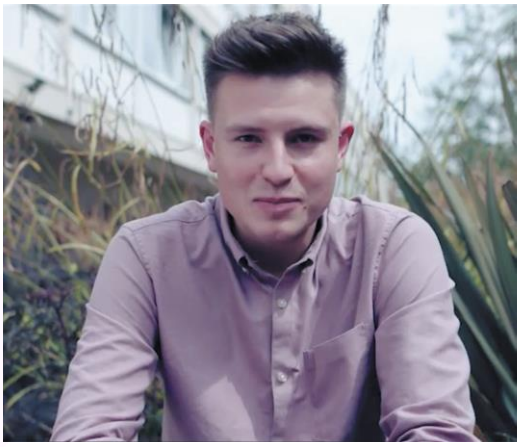

Please look at our video explaining the trial at www.type1diabetesresearch.org.uk/current-trials. The blue box below and the video contains the key points about the study. If you would like to know more, please read the rest of this leaflet.

KEY POINTS ABOUT THE STUDY:

USTEKID

- We want to see if the study medicine, **Ustekinumab**, can make Type 1 Diabetes (the type your child has) easier to manage. The medicine works by "protecting" some of the cells in the pancreas that still produce insulin from attack by the immune system.
- The study will involve your child having an injection every 1-3 months with either the study medicine, Ustekinumab or a placebo (a "dummy medicine"). These injections are given under the skin just like insulin injections and will be done by the study doctor or nurse. Neither you nor the research team will know if your child receives the study medicine or the placebo.
- The medicine is already being used to treat other illnesses quite safely. We will give you information about possible side effects before you decide if your child will take part.
- You will be asked to bring your child to your local hospital or research centre for 10 study visits over a 15 month period, but where possible this will be on the same day as your routine hospital visits. The first two will check if you are eligible to take part. Three of these visits can sometimes be done by a research nurse at home.
- We will ask your child to provide extra blood and urine samples to check that it is safe for them to take part in the study and to check their health, their blood glucose levels and how their immune system is working during the study.
- At three visits, they will have blood tests over a 2 hour period to see how much insulin their body is making.
- We will provide them with a Flash Glucose Monitor (Freestyle Libre) to wear for 2 weeks before each visit. They can keep the monitor for use at home for the whole time of the study.
- Your child will be offered a small gift voucher for each visit and your travel expenses will be paid.
- Your child can stop taking part in the study at any time and they do not have to give a reason why.

For peer review only - http://bmjopen.bmj.com/site/about/guidelines.xhtml

WHY IS THIS STUDY BEING DONE?

This study is being done to see if a medicine called **Ustekinumab** can help to "protect" the cells in the body that produce insulin in young people recently diagnosed with Type 1 Diabetes. It is caused by the body's own immune system damaging the cells in the pancreas that make insulin. Our aim is to develop a treatment that can slow this process by targeting the immune cells causing the damage.

At the time of diagnosis, most children have 10-20% of their insulin-producing cells still working. It usually takes between 1 and 5 years before they stop working completely. Sometimes these last few working cells can make enough insulin to make blood glucose levels stable and easier to control – this is called the "Honeymoon period". This period is only temporary and doesn't last. Ustekinumab, the study medicine, may make this period last longer by reducing the damaging effects of the immune system on the remaining insulin-producing cells in the pancreas.

Ustekinumab is currently given to adults and teenagers with particular skin and bowel problems and it is known to be safe to use and effective at treating those conditions.

WHY HAS MY CHILD BEEN ASKED TO TAKE PART?

Your child has been chosen because they are aged 12 - 18 years old and have recently been diagnosed with Type 1 diabetes.

DOES MY CHILD HAVE TO TAKE PART?

No. It is completely up to both your child and you whether or not they should take part and you or your child can always change your minds at any time. If you and your child are interested in this study then:

- Let us know by calling one of the people listed at the end of this information sheet.
- A member of the research team will contact you to explain more about the study and answer any questions you have.
- If your child wants to take part, they will be asked to agree to sign a form.
- As your child is under 16 years of age, you will need to sign a consent form to say that you agree for your child to take part. The consent form will also ask you to agree to complete three short questionnaires about your child's health. For this reason, it is preferable if the person most likely to attend the study visits with the child is the person who signs the consent form.
- You will be given this information sheet and a copy of your signed consent form to keep.

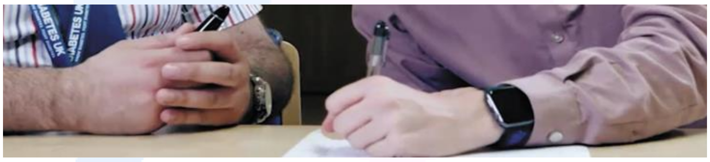

If you make a decision to allow your child to take part, ***you are still free to withdraw them from the study at any time without giving a reason***. This will not affect the medical care they get from their diabetes doctor in any way.

You do not need to decide if you want to allow your child to take part straight away. You can take your time and talk about the study with your child, family, friends and the study team if you want to. You need to let us know within 6 weeks of your child being diagnosed with diabetes, so that we can start the treatment early enough.

WHAT HAPPENS ONCE MY CHILD HAS AGREED TO TAKE PART?

VISIT	WHAT WILL HAPPEN	WHERE AND HOW LONG WILL IT TAKE?
Screening visits	Before we can start the treatment, we need to check that your child is eligible to take part in the study - this is called "screening". You will be asked to bring your child to your local hospital or research centre to talk about the study. This is where you will be able to ask questions.<br><br>If you are happy for your child to take part you will both be asked to sign consent forms before we begin the screening tests which involve:<br><br>Screening visit 1:	Your local hospital or research centre.<br><br>Approximately 1 hour for the first visit and 3 hours

For peer review only - http://bmjopen.bmj.com/site/about/guidelines.xhtml

	• Doing a general health check (this includes a general examination and measuring their height, weight and blood pressure).<br>• Asking about any medicine they are taking and any illnesses they have had or still have.<br>• Taking some blood samples (between 0.5 - 2 tablespoons) from their arm to check their general health and diabetes, as well as testing for infections such as TB (tuberculosis), hepatitis and HIV. Blood volumes vary according to your hospital's local testing procedures.<br>• Taking a chest X-ray to test for TB. There will be one other test for TB which will either be a blood test or a Mantoux test (a skin reaction test), depending on what your hospital's local procedures are.<br>• Taking a urine sample to test for infection, kidney function and a routine pregnancy test for all girls.<br><br>If one of these tests tells us that it is not safe for your child to take part, we will let you know straight away. If the tests are OK, then we will proceed with a second screening visit for a few more tests.<br><br>Screening visit 2:<br>• Doing a general health check.<br>• Asking about any medicine they are taking and any illnesses they have had or still have since their last visit.<br>• Taking a urine sample to test for infection, kidney function and a routine pregnancy test for all girls.<br>• You and your child will be asked to complete a short questionnaire about their diabetes and general health.<br>• Your child will be given a free blood glucose monitor (FreeStyle Libre) and sensors to use for the study. The sensor should be worn two weeks prior to every study visit but can be worn constantly if your child finds it helpful.<br>• A Mixed Meal Tolerance Test (or Milkshake test). This test tells us how much insulin their body is still making. During this test we also take additional blood samples to test the immune system and pancreas function.<br><br>On the day of the Mixed Meal Tolerance Test, you will need to make sure that your child has not eaten or drunk except water anything from midnight the night before onwards. They will also be asked to not take their early morning short acting insulin because they will not be eating breakfast. You will need to tell us your child's blood glucose levels on waking so that we can make sure it's OK for them to be tested. They will need to be between 4.0 and 11.1mmol/L for the screening visit to happen. If their blood glucose level runs low before coming to the hospital and needs to be treated, the test will be rescheduled but if they are higher, your child may be advised to take short acting insulin so that the visit can go ahead.<br><br>At the hospital, your child will have blood taken through a small plastic tube (cannula) which we will insert in their arm (using local anaesthetic cream/spray if they want it). This will stay in their arm during the test so that we can take blood samples more easily.<br><br>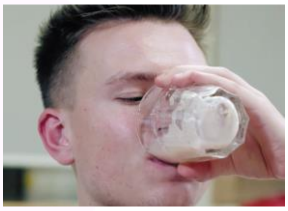<br>Then they will be given a milkshake to drink (various flavours available). The research doctor or nurse will take blood at fixed times over the next 2 hours. Over this time, less than 1 tablespoon (10ml) of blood will be taken from them in total. During this time, they can relax on a bed, play games, read or study. Once the test is completed we will give them something to eat and drink and they will receive insulin in whatever dose is needed.<br><br>An additional 40mls (less than 3 tablespoons) will be taken at the same visit for testing in our laboratories.<br><br>The blood samples will be analysed within 2 weeks and if the test shows that they are still making some of their own insulin, you will be contacted by the research team to tell you that everything is OK for your child to be part of the study and to arrange the first injection.	for the second visit.

For peer review only - http://bmjopen.bmj.com/site/about/guidelines.xhtml

	If it is not convenient to have two separate visits for testing, we can arrange to combine the two sets of tests if you let us know beforehand. This is because the combined screening visit needs your child to be fasted on arrival.	
**Study Visit 1**	If your child is eligible to take part in this study, they will be randomly allocated to either the study medicine group or the placebo (a "dummy" medicine that has no effect) group. This is decided by chance using a computer programme before the study visit and neither you nor the research team will know until the end of the study what treatment they received.<br>2 out of every 3 people taking part will receive the study medicine compared with only 1 out of 3 getting the placebo. This is to give people a better chance of getting the study medicine.<br>Treatment visits will be booked in so that your child will receive injections at the required intervals. The second dose will be four weeks after the first. All other doses afterwards will be eight weeks apart. These will be booked in advance so that any issues with attendance can be identified as soon as possible (e.g. holidays, exams). Postponing a treatment visit may result in the treatment being stopped if too much time has passed because the levels of the study medicine in your child's body may go too low and won't work anymore.Your child will have a physical examination and tests will be done on their urine and blood samples (57.5ml which is about 3 and a half tablespoons) at this visit.<br>Then they will receive an injection of either the study medicine, Ustekinumab, or the placebo. Injections are given under the skin using a very small needle similar to the one that they already use for daily insulin injections.<br>They will be asked to stay in the hospital for 1 hour after they receive the injection so that the study team can make sure that there are no side effects and that your child are safe to leave.	Your local hospital or research centre.<br>Approximately 2 hours.
**Study Visit 2**<br>*4 weeks after the 1st visit*	Your child will have a physical examination and be asked questions about your health. We will also need a urine sample and a blood sample (up to 50.5ml which is just under 3 tablespoons). Then your child will receive an injection of either the study medicine, Ustekinumab or placebo, depending on which treatment group they are in.<br>We will also download the data stored on their blood glucose monitor.	Your local hospital or research centre.<br>Approximately 1 hour
**Study Visit 3**<br>*12 weeks into the study*	Your child will have a physical exam and be asked questions about your health. We will also need a urine sample and a blood sample (up to 59.5ml which is nearly 3 and a half tablespoons).<br>Next your child will receive an injection of either the study medicine, Ustekinumab or placebo, depending on which treatment group they are in.<br>We will also download the data stored on their blood glucose monitor.	Your local hospital or research centre.<br>Approximately 1 hour
**Study Visit 4**<br>*20 weeks into the study*	At this visit your child will receive an injection of either the study medicine or placebo. They will also have a urine test but no blood sample will be needed.<br>This appointment may be done at your home.	Your local hospital or research centre or your home.<br>Approximately 1 hour
**Study Visit 5**<br>*28 weeks into the study*	Your child will be asked to do a second milkshake test in exactly the same way as described earlier and will involve taking less than 1 tablespoon (10ml) of blood over a 2 hour period. This means that they have to arrive fasted for this study visit.<br>Your child will also have a physical examination, and be asked questions about their health. We will also need a urine sample and another blood sample (up to 61.5ml which is 3 and a half tablespoons).<br>Then your child will receive an injection of either the study medicine, Ustekinumab or placebo, depending on which treatment group they are in.<br>This visit will also include both you and your child completing a short questionnaire exactly like the one you did at the screening visit.<br>We will also download the data stored on their blood glucose monitor.	Your local hospital or research centre.<br>Approximately 3 hours

For peer review only - http://bmjopen.bmj.com/site/about/guidelines.xhtml

**Study Visit 6**<br>36 weeks into the study	At this visit your child will receive an injection of either the study medicine or placebo. They will also have a urine test but no blood sample will be needed.<br>This appointment may be done at your home.	Your local hospital or research centre or your home.<br>Approximately 1 hour
**Study Visit 7**<br>44 weeks into the study	At this visit your child will receive the <u>FINAL</u> injection of either the study medicine or placebo. They will also have a urine test but no blood sample will be needed.<br>This appointment may be done at your home.<br>We will also download the data stored on their blood glucose monitor.	Your local hospital or research centre or your home.<br>Approximately 1 hour
**Study Visit 8 – follow up**<br>52 weeks into the study	At this final visit your child will have a physical examination and be asked questions about their health. They will be asked to do a final milkshake test in exactly the same way as described earlier and will involve taking half a tablespoon (10ml) of blood over a 2 hour period.<br>We will also need a urine sample and another blood sample (up to 61.5ml which is 3 and a half tablespoons).<br>This visit will also include both you and your child completing a short questionnaire exactly like the one you did at the screening visit.<br>We will also download the data stored on their blood glucose monitor.	Your local hospital or research centre.<br>Approximately 3 hours
**Remote follow up**<br>Weeks 78 & 104	We will check your child's hospital records to find out how they are doing. They do not need to come into the hospital for a visit. We may need to call you up at home to check that they are OK and in good health.	No visit needed

WHAT ELSE WILL MY CHILD AND I BE ASKED TO DO?

As well as attending with your child to your local hospital or research centre for study visits there are a few other things we will ask your child to do during the study which may involve your help:

- Your child will be asked to complete a diary between study visits to record:
  a) How much insulin they take during the study (for the <u>two weeks before every study visit</u>).
  b) If they feel or have been unwell or have to take any other medicines during the study.
  c) If they have any hypoglycaemic (low blood glucose levels) episodes that need treating.

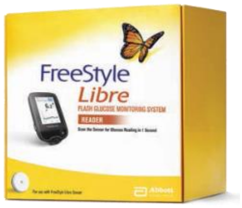

- We will ask them to test their blood glucose levels at home for at least 2 weeks before each visit using our FREE Abbott Freestyle Libre glucose monitoring system so they don't need to do extra finger prick tests for the trial. They will need to wear a sensor on their arm for the two weeks before our study visit if they want to be in the trial. We will show you both how it works. They are free to use the monitor at home for the rest of the time of study if they want to. We will give them enough sensors to allow this for a year.

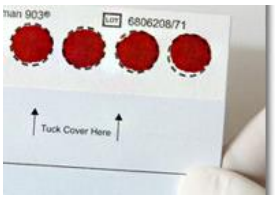

- We will ask them to give a blood spot sample which they can do at home. They will need to do this once a week for 28 weekly, then every month for the next 6 months. The test involves pricking their finger like a normal finger prick blood glucose test and dabbing the blood spot into a special card. They will need to do this before the first meal of the day and then 1 hour later. The paper should be posted to a special laboratory for testing. We will show you both how to do this and will provide envelopes and pay for the postage.

You will be asked to make sure that your child does not have certain vaccinations before, during and immediately after the study. If they need a vaccination, for example if they are travelling abroad, you must tell the study doctor or nurse immediately.

Please be aware that a urine pregnancy test will be done for all females at each study visit. We need to do this

For peer review only - http://bmjopen.bmj.com/site/about/guidelines.xhtml

because the law requires us to do this in clinical trials because the effects of the study medicine are not known in pregnancy and we want the mother and baby to be safe. Any confirmed pregnancies will be monitored closely with your permission (including the female partners of male participants). Rather than asking if your child is engaged in actions that may lead to pregnancy, we will ask everybody (males and females) to agree to use adequate contraception (hormonal based contraception, barrier contraception, abstinence) until 4 months following the date of their final treatment. You will need to agree to this on behalf of your child if you want them to take part. Your GP or a pharmacist can advise on suitable contraception for your child.

Finally, we would like you to complete a short questionnaire about your child's health and diabetes. These questionnaires will be done at the second screening visit and study visits 5 and 8. It is important that the person who consents will complete all three questionnaires.

WILL THE STUDY HELP MY CHILD?

If your child has been allocated to the group that receives the study medicine, Ustekinumab, it is possible that it will help their pancreas make insulin for longer. However we cannot say this for certain until we have completed this study. During the study your child's diabetes will be very closely monitored. This will include regular check-ups with your local diabetes team including routine blood testing. You and your child will have more time with the research team to discuss their diabetes and ask questions than at a normal clinic appointment.

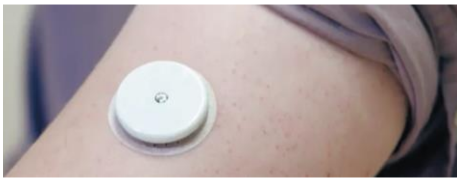

Your child will be provided with a FREE Abbott Freestyle Libre blood glucose monitoring system. They can use this to check their blood glucose levels while they are in the study, although they will still need to do some finger prick tests. The treatments will stop at week 44 and your child will not receive any further injections of the study drug / placebo during the trial.

WHAT HAPPENS WHEN THE STUDY STOPS?

We will collect all the information together and we will decide if the study medicine can help people with Type 1 Diabetes make their own insulin for longer. If it does then we will carry out a bigger version of this study. You and your child will be informed which treatment they were given and their medical records will be updated with the treatment information.

WHAT IF NEW INFORMATION COMES ALONG?

Sometimes during research, we get new information about the treatment being studied. If this happens, we will tell you about it and discuss whether you and your child want to continue in the study.

WHAT IF I MY CHILD DOES NOT WANT TO TAKE PART ANYMORE?

Just let your study doctor or research nurse know about your decision. You and your child will be asked whether you wish to withdraw from just having the study treatment or from the whole study (including the study visits and data collection). You can withdraw from treatment but still come to study visits for sample and data collection. If they want to withdraw completely, we will make a note of this and we will make sure that your child are transferred back to normal care as quickly as possible.

WHAT IF THERE IS A PROBLEM OR SOMETHING GOES WRONG?

If your child feels unwell or suffers any unusual discomfort during the study it is important to inform the study doctor or nurse as soon as possible. If it is because of something in the study, we need to consider stopping your child's treatment.

If you or your child feel overwhelmed by their recent diagnosis, you can call the local [title] on [tel number / email] and they can talk through any concerns with you both.

If you are unhappy about the conduct of the study and wish to complain, you can do this through:

(name and contact details of appropriate organisations – site specific).

WHAT ARE THE POSSIBLE SIDE EFFECTS FOR MY CHILD IN PARTICIPATING IN THE STUDY?

They may get a bruise or a little discomfort at the site of the blood tests.

The FreeStyle Libre sensor may cause a slight rash for some people who might be allergic to the adhesive on the

For peer review only - http://bmjopen.bmj.com/site/about/guidelines.xhtml

sensor. The manufacturer are always improving their sensors to stop this happening but your child may experience some discomfort from wearing the sensor. Please let the research team know if this happens.

During the Milkshake test, they may experience changes in blood glucose level because they will not have taken insulin immediately beforehand. The study nurses and doctors will be available to help your child make any changes to their usual insulin doses after this test.

The medicine (Ustekinumab) being used in this study is currently used in patients with skin and bowel conditions. Because the medicine acts on the immune system, there is a possibility that it will increase the risk of infections and cancer, but so far, this has not been found to be a problem with people treated with this medicine for others diseases. It is also possible that they may get an allergic reaction to the treatment injection. We will ask them to stay for one hour after their first injection to check for any reactions.

It is routine to check people who receive this medicine for tuberculosis (TB) as, if your child have this infection, the study medicine may make it worse. We will check your child and if there is evidence of TB infection, they will not be allowed to take part.

If your child takes part in this study, they will have a chest X-ray to rule out TB which is additional to standard care. Chest x-rays involve using ionising radiation to form images of the body. Ionising radiation can cause cell damage in the longer term which can sometimes lead to cancer developing. However, we only ask for one x-ray so taking part in this study will add only a very small chance of this happening to them when they are older. The risk is not much greater than that found with natural background radiation.

If your child feels ill at any time during the trial and you go to your GP or the hospital, please show them the membership card your child will be given so that they can contact the research team to ask about possible side effects.

The research team will carefully monitor your child throughout the study to check their health and to ensure that they are not experiencing any side effects. You must tell someone straight away if they complain that they feel unwell.

WILL MY CHILD RECEIVE ANY PAYMENT FOR TAKING PART?

Your child will receive a £10.00 gift voucher for each treatment visit and we will give them £30.00 gift if they come to the final visit (visit 8) (that's £100 in total if they come to all visits).

You will be able to claim back your travel expenses for getting your child to the local hospital or research centre for all screening, treatment and follow up visits.

WHAT INFORMATION WILL YOU COLLECT AND HOW WILL IT BE KEPT PRIVATE?

We will ask for your name and contact details as well as your child's name so that the research nurse can keep in touch and manage your visits. We will also need to collect data about your child's health which we get from their tests, their blood glucose monitor and their medical records. We will also ask you and your child to complete a questionnaire each at three time points.

The people in our research team at the local hospital or research centre will know that your child is taking part. The doctors looking after your child when you come to hospital will also know that they are in the study. Their medical notes may be looked at by staff from Swansea and Cardiff Universities or NHS and regulatory auditors who will be checking that the study is being done correctly. If you agree, we will also tell your family doctor (GP) that they are in the study.

People at the research laboratories will not know who your child is when they test their samples. Your child will be given a study number to replace their name so any study samples and data related to them will be anonymised. The questionnaires and the data from your flash glucose monitor will also use study numbers instead of names.

All information collected about your child during the study will be kept by the research nurse in a locked cabinet and entered onto a secure database. Only people with the password can open up the database.

Cardiff University is the sponsor for this study based in the United Kingdom. Cardiff University will be using information from your child and their medical records in order to undertake this study and will act as the data controller for this study. This means that we are responsible for looking after their information and using it properly. Cardiff University will keep identifiable information about them for 25 years after the study has finished. Your rights to access, change or move the information about your child are limited, as we need to manage their information in specific ways in order for the research to be reliable and accurate. If you/they withdraw from the study, we will keep

For peer review only - http://bmjopen.bmj.com/site/about/guidelines.xhtml

the information about them that we have already obtained. To safeguard their rights, we will use the minimum personally-identifiable information possible. You can find out more about how we use your child's information by contacting inforequest@cardiff.ac.uk.

WHAT WILL HAPPEN TO ANY SAMPLES THEY GIVE?

We want to test your child's blood and urine to better understand your diabetes and how the study medicine or placebo affects their diabetes. The blood samples will be used to test:

1. The amount of insulin their body still makes before and after taking part in the study.
2. Their average blood glucose levels
3. The antibodies to the insulin making cells
4. General health checks – anaemia, kidneys, liver etc
5. How much of the study medication is in their blood.
6. How their body's immune system is reacting to the study medication.

Scientists in laboratories around the UK will look at their anonymised blood samples. These samples will be stored in a safe place. Some of their blood samples will be sent to a laboratory in Europe and either America or Canada for special testing to find out how much of the study medicine is present.

We would like to keep any leftover blood samples in a special tissue repository permanently - we will ask for your permission to do this. The samples will only be accessed by scientists who have special permission to do so. The samples might be sent outside the UK to other research teams in Europe or countries such as America and Canada if you agree to this. These other teams must have permission from us to use your sample before we send it. If you do not want to agree to this, their samples will be destroyed after they have been analysed.

WILL ANY GENETIC TESTS BE DONE?

We will use their blood samples to help us study the genes involved with diabetes and the immune system. These samples will not have your name on them and will not be used for any other reason without your permission.

WHAT WILL HAPPEN TO THE RESULTS OF THIS STUDY?

The full results of this study will not be known until the last patient has completed their tests, which may take more than 5 years. The research results will be reported in scientific publications and meetings but you will not be identified by name at all. If you are interested in receiving a summary of the research results, we can arrange this.

WHO IS ORGANISING AND FUNDING THE STUDY?

CARDIFF UNIVERSITY PRIFYSGOL CAERDYDD

Swansea University Prifysgol Abertawe
Swansea Trials Unit Uned Dreialon Abertawe

The study is being organised by researchers at Cardiff University and Swansea University.

It is being funded by a grant from the National Institute for Health and Research (NIHR).

NHS
National Institute for Health Research

WHERE IS THE STUDY BEING DONE?

The study is being done at hospitals and research centres across England, Wales and Scotland.

WHO HAS CHECKED THIS STUDY?

Before any research goes ahead it has to be checked by a Research Ethics Committee. This is a group of people who make sure that the research is OK to do and to make sure that the patient will be safe. This study has been looked at by Wales REC 3. As this study is looking at a medicine, it has also been approved by the government's Medicine and Healthcare products Regulatory Authority (MHRA) who check that the researchers carry out the study safely. It has also been checked by national and local NHS organisations to make sure that the study can be done using their site and staff.

WHAT SHOULD I DO NOW?

If you are interested in taking part, or have any questions please contact one of the following people:

Name:	Name:	Name: (only if needed)

For peer review only - http://bmjopen.bmj.com/site/about/guidelines.xhtml

**Role:** Principal Investigator<br>**Tel. No:**<br>**Email:**	**Role:** Research nurse<br>**Tel. No:**<br>**Email:**	**Role:**<br>**Tel. No:**<br>**Email:**

Alternatively, you may want to speak to someone at the USTEKID Trial Office who are managing the study, based at Swansea University. The Trial Manager's details are below:

Name: Dr Kym Thorne
Tel. No: 01792 606372 (direct) or 01792 606545 for Swansea Trials Unit
Email: ustekid@swansea.ac.uk
Address: Floor 2, Institute of Life Sciences 2, Swansea University Medical School, Singleton Park, Swansea SA2 8PP

Thank you for taking the time to read this information sheet and for considering taking part in this research study

For peer review only - http://bmjopen.bmj.com/site/about/guidelines.xhtml

USTEKID

National Institute for Health Research

CARDIFF UNIVERSITY PRIFYSGOL CAERDYDD

Swansea University Prifysgol Abertawe
Swansea Trials Unit
Uned Dreialon Abertawe

A research study to see if the medicine Ustekinumab can make diabetes easier to manage

CONTACT DETAILS FOR STUDY TEAM:

NURSE:

DOCTOR:

EMERGENCIES:

FOR YOUNG PEOPLE AGED 16-18 YEARS OLD

We would like you to help us with our research study. Please read this information carefully and talk to your parent or carer about the study. Ask us if there is anything that is not clear or if you want to know more. Take time to decide if you want to take part. It is up to you if you want to do this. If you do not, then that is fine, you will be looked after by your doctors just the same.

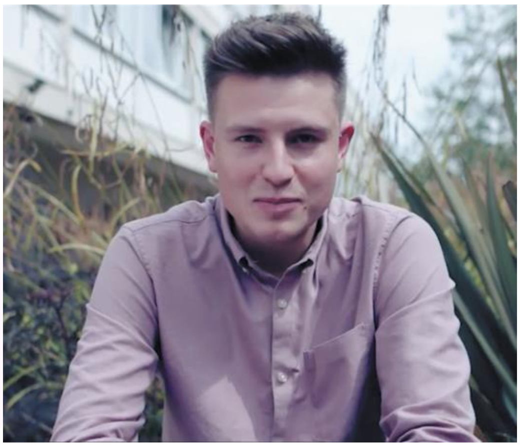

Please look at our video explaining the trial at www.type1diabetesresearch.org.uk/current-trials. The blue box below and the video contains the key points about the study. If you would like to know more, please read the rest of this leaflet.

KEY POINTS ABOUT THE STUDY:

USTEKID

- We want to see if the study medicine, **Ustekinumab**, can make Type 1 Diabetes (the type you have) easier to manage. The medicine works by "protecting" some of the cells in the pancreas that still produce insulin from attack by the immune system.
- The study will involve you having an injection every 1-3 months with either the study medicine, Ustekinumab or a placebo (a "dummy medicine"). These injections are given under the skin just like insulin injections and will be done by the study doctor or nurse. Neither you nor the research team will know if you receive the study medicine or the placebo.
- The medicine is already being used to treat other illnesses quite safely. We will give you information about possible side effects before you decide if you will take part.
- You will be asked to come into your local hospital or research centre for 10 study visits over a 15 month period, but where possible this will be on the same day as your routine hospital visits. The first two will check if you are eligible to take part. Three of these visits can sometimes be done by a research nurse at home.
- We will ask you to provide extra blood and urine samples to check that it is safe for you to take part in the study and to check your health, your blood glucose levels and how your immune system is working during the study.
- At three visits, you will have blood tests over a 2 hour period to see how much insulin your body is making.
- We will provide you with a glucose monitor (Freestyle Libre) to wear for 2 weeks before each visit. You can keep the monitor for use at home for the whole time of the study.
- You will be offered a small gift voucher for each visit you come to and your travel expenses will be paid.
- You can stop taking part in the study at any time and you do not have to give a reason why.

For peer review only - http://bmjopen.bmj.com/site/about/guidelines.xhtml

WHY IS THIS STUDY BEING DONE?

This study is being done to see if a medicine called **Ustekinumab** can help to "protect" the cells in the body that produce insulin in young people recently diagnosed with Type 1 Diabetes. It is caused by the body's own immune system damaging the cells in the pancreas that make insulin. Our aim is to develop a treatment that can slow this process by targeting the immune cells causing the damage.

At the time of diagnosis, most people your age have 10-20% of their insulin-producing cells still working. It usually takes between 1 and 5 years before they stop working completely. Sometimes these last few working cells can make enough insulin to make blood glucose levels stable and easier to control – this is called the "Honeymoon period". This period is only temporary and doesn't last. Ustekinumab, the study medicine, may make this period last longer by reducing the damaging effects of the immune system on the remaining insulin-producing cells in the pancreas.

Ustekinumab is currently given to adults and teenagers with particular skin and bowel problems and it is known to be safe to use and effective at treating those conditions.

WHY HAVE I BEEN ASKED TO TAKE PART?

You have been chosen because you are aged 12 - 18 years old and have recently been diagnosed with Type 1 diabetes.

DO I HAVE TO TAKE PART?

No. It is completely up to you whether or not you take part and you can always change your mind at any time. If you are interested in this study then:

- Let us know by calling one of the people listed at the end of this information sheet.
- A member of the research team will contact you to explain more about the study and answer any questions you have.
- If you agree, you will sign a consent form and be given a copy of your signed consent form and this information sheet to keep.

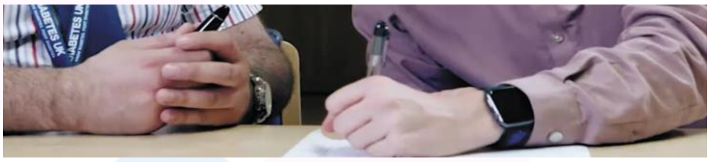

If you make a decision to take part, ***you are still free to withdraw from the study at any time without giving a reason***. This will not affect the medical care you get from your diabetes doctor in any way.

You do not need to decide if you want to take part straight away. You can take your time and talk about the study with your family, friends and the study team if you want to. You need to let us know within 6 weeks of being diagnosed with diabetes, so that we can start the treatment early enough.

WHAT HAPPENS ONCE I HAVE AGREED TO TAKE PART?

VISIT	WHAT WILL HAPPEN	WHERE AND HOW LONG WILL IT TAKE?
Screening visits	Before we can start the treatment, we need to check that you are eligible to take part in the study - this is called "screening". You will be asked to come to your local hospital or research centre to talk about the study. This is where you will be able to ask questions.<br>If you are happy to take part you will be asked to sign a consent form before we begin the screening tests which involve:<br>Screening visit 1:<br>• Doing a general health check (this includes a general examination and measuring your height, weight and blood pressure).<br>• Asking about any medicine you are taking and any illnesses you have had or still have.<br>• Taking some blood samples (between 0.5 - 2 tablespoons) from your arm to check your general health and diabetes, as well as testing for infections such as TB (tuberculosis), hepatitis and HIV. Blood volumes vary according to your hospital's local testing procedures.	Your local hospital or research centre.<br><br>Approximately 1 hour for the first visit and 3 hours for the second visit.

For peer review only - http://bmjopen.bmj.com/site/about/guidelines.xhtml

<table>
<tr>
<td></td>
<td>

- Taking a chest X-ray to test for TB. There will be one other test for TB which will either be a blood test or a Mantoux test (a skin reaction test), depending on what your hospital's local procedures are.
- Taking a urine sample to test for infection, kidney function and a routine pregnancy test for all girls.

If one of these tests tells us that it is not safe for you to take part, we will let you know straight away. If the tests are OK, then we will proceed with a second screening visit for a few more tests.

Screening visit 2:

- Doing a general health check.
- Asking about any medicine you are taking and any illnesses you have had or still have since your last visit.
- Taking a urine sample to test for infection, kidney function and a routine pregnancy test for all girls.
- You and your parent/carer will be asked to complete a short questionnaire about your diabetes and general health.
- You will be given a free blood glucose monitor (FreeStyle Libre) and sensors to use for the study. The sensor should be worn two weeks prior to every study visit but can be worn constantly if you find it helpful.
- A Mixed Meal Tolerance Test (or Milkshake test). This test tells us how much insulin your body is still making. During this test we also take additional blood samples to test the immune system and pancreas function.

On the day of the Mixed Meal Tolerance Test, you will need to make sure that you have not eaten or drunk anything except water from midnight the night before onwards. You will also be asked to not take your early morning short acting insulin because you will not be eating breakfast. You will need to tell us your blood glucose levels on waking so that we can make sure it's OK for you to be tested. It will need to be between 4.0 and 11.1mmol/L for the screening visit to happen. If it is lower, the test will be rescheduled but if it is higher, you may be advised to take short acting insulin so that the visit can go ahead.

.

At the hospital, you will have blood taken through a small plastic tube (cannula) which we will insert in your arm (using local anaesthetic cream/spray if you want it). This will stay in your arm during the test so that we can take blood samples more easily.

Then you will be given a milkshake to drink (various flavours available). The research doctor or nurse will take blood at fixed times over the next 2 hours. Over this time, less than 1 tablespoon (10ml) of blood will be taken from you in total. During this time, you can relax on a bed, play games, read or study. Once the test is completed we will give you something to eat and drink and you will receive insulin in whatever dose is needed.

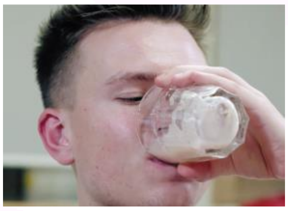

An additional 40mls (less than 3 tablespoons) will be taken at the same visit for testing in our laboratories.

Your blood samples will be analysed within 2 weeks and if the test shows that you are still making some of your own insulin, you will be contacted by the research team to tell you that everything is OK to be part of the study and to arrange the first injection.

If it is not convenient to have two separate visits for testing, we can arrange to combine the two sets of tests if you let us know beforehand. This is because the combined screening visit needs you to be fasted on arrival.

</td>
<td></td>
</tr>
<tr>
<td>

Study Visit 1

</td>
<td>

If you take part in this study you will be randomly allocated to either the study medicine group or the placebo (a "dummy" medicine that has no effect) group. This is decided by chance using a computer programme before the study visit and neither you nor the research team will know until the end of the study what treatment you have received.

2 out of every 3 people taking part will receive the study medicine compared with only 1 out of 3 getting the placebo. This is to give people a better chance of getting the study medicine.

</td>
<td>

Your local hospital or research centre.

Approximately 2 hours.

</td>
</tr>
</table>

For peer review only - http://bmjopen.bmj.com/site/about/guidelines.xhtml

	Treatment visits will be booked in so that you receive your injection at the required intervals. The second dose will be four weeks after the first. All other doses afterwards will be eight weeks apart. These will be booked in advance so that any issues with attendance can be identified as soon as possible (e.g. holidays, exams). Postponing a treatment visit may result in the treatment being stopped if too much time has passed because the levels of the study medicine in your body may go too low and won't work anymore.<br><br>You will have a physical examination and tests will be done on your urine and blood samples (up to 57.5ml which is about 3 and a half tablespoons) at this visit.<br><br>Then you will receive an injection of either the study medicine, Ustekinumab, or the placebo. Injections are given under the skin using a very small needle similar to the one that you already use for daily insulin injections.<br><br>You will be asked to stay in the hospital for 1 hour after you receive the injection so that the study team can make sure that there are no side effects and that you are safe to leave.	
**Study Visit 2**<br><br>*4 weeks after the 1st visit*	You will have a physical examination and be asked questions about your health. We will also need a urine sample and a blood sample (up to 50.5ml which is just under 3 tablespoons). Then you will receive an injection of either the study medicine, Ustekinumab or placebo, depending on which treatment group you are in.<br><br>We will also download the data stored on your blood glucose monitor.	Your local hospital or research centre.<br><br>Approximately 1 hour
**Study Visit 3**<br><br>*12 weeks into the study*	You will have a physical exam and be asked questions about your health. We will also need a urine sample and a blood sample (up to 59.5ml which is nearly 3 and a half tablespoons).<br><br>Next you will receive an injection of either the study medicine, Ustekinumab or placebo, depending on which treatment group you are in.<br><br>We will also download the data stored on your blood glucose monitor.	Your local hospital or research centre.<br><br>Approximately 1 hour
**Study Visit 4**<br><br>*20 weeks into the study*	At this visit you will receive an injection of either the study medicine or placebo. You will also have a urine test but no blood sample will be needed.<br><br>This appointment can be done at your home.	Your local hospital or research centre or your home.<br><br>Approximately 1 hour
**Study Visit 5**<br><br>*28 weeks into the study*	You will be asked to do a second milkshake test in exactly the same way as described earlier and will involve taking half a tablespoon (10ml) of blood over a 2 hour period. This means that you have to arrive fasted for this study visit.<br><br>You will also have a physical examination, and be asked questions about your health. We will also need a urine sample and another blood sample (up to 61.5ml which is 3 and a half tablespoons).<br><br>Then you will receive an injection of either the study medicine, Ustekinumab or placebo, depending on which treatment group you are in.<br><br>This visit will also include both you and your parent/carer completing a short questionnaire exactly like the one you did at the screening visit.<br><br>We will also download the data stored on your blood glucose monitor.	Your local hospital or research centre.<br><br>Approximately 3 hours
**Study Visit 6**<br><br>36 weeks into the study	At this visit you will receive an injection of either the study medicine or placebo. You will also have a urine test but no blood sample will be needed.<br><br>This appointment can be done at your home.	Your local hospital or research centre or your home.<br><br>Approximately 1 hour
**Study Visit 7**<br><br>44 weeks into the study	At this visit you will receive the FINAL injection of either the study medicine or placebo. You will also have a urine test but no blood sample will be needed.<br><br>This appointment can be done at your home.<br><br>We will also download the data stored on your blood glucose monitor.	Your local hospital or research centre or your home.<br><br>Approximately 1 hour
**Study Visit 8 – follow up**	At this final visit you will have a physical examination and be asked questions about your health. You will be asked to do a final milkshake test in exactly the same way as described earlier and will involve taking half a tablespoon (10ml) of blood over a 2 hour period.	Your local hospital or research centre.

For peer review only - http://bmjopen.bmj.com/site/about/guidelines.xhtml

52 weeks into the study	We will also need a urine sample and another blood sample (up to 61.5ml which is 3 and a half tablespoons).<br>This visit will also include both you and your parent/carer completing a short questionnaire exactly like the one you did at the screening visit.<br>We will also download the data stored on your blood glucose monitor.	Approximately 3 hours
**Remote follow up**<br>Weeks 78 & 104	We will check your hospital records to find out how you are doing. You do not need to come into the hospital for a visit. We may need to call you up at home to check that you are OK and in good health.	No visit needed

WHAT ELSE WILL I BE ASKED TO DO?

As well as coming to your local hospital or research centre for study visits there are a few other things we will ask you to do during the study:

- You will be asked to complete a diary between study visits to record:
  a) How much insulin you take during the study (for the two weeks before every study visit)
  b) If you feel or have been unwell or have to take any other medicines during the study.
  c) If you have any hypoglycaemic (low blood glucose levels) episodes that need treating.

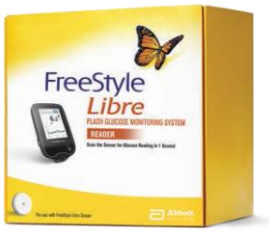

- We will ask you to test your blood glucose levels at home for at least 2 weeks before each visit using our FREE Abbott Freestyle Libre flash glucose monitoring system so you don't need to do extra finger prick tests for the trial. You will need to wear a sensor on your arm for the two weeks before our study visit if you want to be in the trial. We will show you how it works. You are free to use the monitor at home for the rest of the time of study if you want to. We will give you enough sensors to allow this for a year.

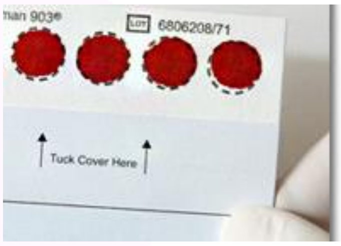

- We will ask you to give blood spot samples which you can do at home. You will need to do this once a week for 28 weeks, then every month for the next 6 months. The test involves pricking your finger like a normal finger prick blood glucose test and dabbing the blood spot onto a special card. You will need to do this before the first meal of the day and then 1 hour later. The card should be posted to our special laboratory for testing. We will show you how to do this and will provide envelopes and pay for the postage.
- You will be asked to make sure that you do not have certain vaccinations before, during and immediately after the study. If you need a vaccination, for example if you are travelling abroad, you must tell the study doctor or nurse immediately.
- Please be aware that a urine pregnancy test will be done for all females at each study visit. We need to do this because the law requires us to do this in clinical trials because the effects of the study medicine are not known in pregnancy and we would want the mother and baby to be safe. Any confirmed pregnancies will be monitored closely with your permission (including the female partners of male participants).
- Rather than asking if you are engaged in actions that may lead to pregnancy, we will ask everybody to agree to use adequate contraception (hormonal based contraception, barrier contraception, abstinence) until 4 months following the date of their final treatment. All participants will need to agree to this to take part. Your GP or a pharmacist can advise on suitable contraception if you don't want to discuss this with your parents/carers.
- Finally, we would like you to complete a short questionnaire about your health and diabetes. These questionnaires will be done at the second screening visit and study visits 5 and 8. Your parent/carer will also be asked to complete questionnaires at these time points so we will ask that they agree to attend those study visits with you.

WILL THE STUDY HELP ME?

If you have been allocated to the group that receives the study medicine, Ustekinumab, it is possible that it will help your pancreas make insulin for longer. However, we cannot say this for certain until we have completed this study.

For peer review only - http://bmjopen.bmj.com/site/about/guidelines.xhtml

During the study your diabetes will be very closely monitored. This will include regular check-ups with your local diabetes team including routine blood testing. You will have more time with the research team to discuss your diabetes and ask questions than at a normal clinic appointment.

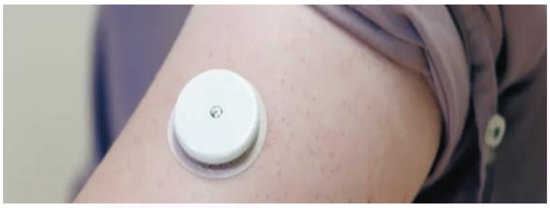

You will be provided with a FREE Abbott Freestyle Libre flash glucose monitoring system. You can use this to check your blood sugar levels while you are in the study, although you will still need to do some finger prick tests. The treatments will stop at week 44 and you will not receive any further injections of the study drug / placebo during the trial.

WHAT HAPPENS WHEN THE STUDY STOPS?

We will collect all the information together and we will decide if the study medicine can help people with Type 1 Diabetes make their own insulin for longer. If it does then we will carry out a bigger version of this study.

You will be informed which treatment you were given and your medical records will be updated with the treatment information.

WHAT IF NEW INFORMATION COMES ALONG?

Sometimes during research, we get new information about the treatment being studied. If this happens, we will tell you about it and discuss whether you want to continue in the study.

WHAT IF I DO NOT WANT TO TAKE PART ANYMORE?

Just let your study doctor or research nurse know about your decision. You will be asked whether you wish to withdraw from just having the study treatment or from the whole study (including the study visits and data collection).

You can withdraw from treatment but still come to study visits for sample and data collection. If you want to withdraw completely, we will make a note of this and we will make sure that you are transferred back to normal care as quickly as possible.

WHAT IF THERE IS A PROBLEM OR SOMETHING GOES WRONG?

If you feel unwell or suffer any unusual discomfort during the study it is important to inform the study doctor or nurse as soon as possible. If it is because of something in the study, we need to consider stopping your treatment. The diabetes care team will also be informed.

If you feel overwhelmed by your recent diagnosis, you can call the local [title] on [tel number / email] and they can talk through your concerns with you.

If you are unhappy about the conduct of the study and wish to complain, you can do this through:

(name and contact details of appropriate organisations – site specific).

WHAT ARE THE POSSIBLE SIDE EFFECTS FOR ME IN PARTICIPATING IN THE STUDY?

You may get a bruise or a little discomfort at the site of the blood tests.

The FreeStyle Libre sensor may cause a slight rash for some people who might be allergic to the adhesive on the sensor. The manufacturer are always improving their sensors to stop this happening but you may experience some discomfort from wearing the sensor. Please let the research team know if this happens.

During the Milkshake test, you may experience changes in blood glucose level because you will not have taken insulin immediately beforehand. The study nurses and doctors will be available to help you make any changes to your usual insulin doses after this test.

The medicine (Ustekinumab) being used in this study is currently used in patients with skin and bowel conditions safely. Because the medicine acts on the immune system, there is a possibility that it will increase the risk of infections and cancer, but so far this has not been found to be a problem with people treated with this medicine for others diseases. It is also possible that you may get an allergic reaction to the treatment injection. We will ask you to stay for one hour after your first injection to check for any reactions.

It is routine to check people who receive this medicine for tuberculosis (TB) as if you have this infection the study medicine may make it worse. If there is evidence of TB infection, you will not be allowed to take part.

If you take part in this study, you will have a chest X-ray to rule out TB which is additional to standard care. Chest x-rays involve using ionising radiation to form images of the body. Ionising radiation can cause cell damage in the

For peer review only - http://bmjopen.bmj.com/site/about/guidelines.xhtml

longer term which can sometimes lead to cancer developing. However, we only ask for one x-ray so taking part in this study will add only a very small chance of this happening to you when you are older. The risk is not much greater than that found with natural background radiation.

If you feel ill at any time during the trial and go to your GP or the hospital, please show them the membership card you will be given so that they can contact the research team to ask about possible side effects.

The research team will carefully monitor you throughout the study to check your health and to ensure that you are not experiencing any side effects. You must tell someone straight away if you feel unwell.

WILL I RECEIVE ANY PAYMENT FOR TAKING PART?

You will receive a £10.00 gift voucher for each treatment visit and we will give you £30.00 gift if you come to the final visit (visit 8) (that's £100 in total if you come to all visits).

You will be able to claim back your travel expenses for getting to the local hospital or research centre for all screening, treatment and follow up visits.

WHAT INFORMATION WILL YOU COLLECT AND HOW WILL IT BE KEPT PRIVATE?

We will ask for your name and contact details so that the research nurse can keep in touch and manage your visits. We will also need to collect data about your health that we get from your tests, your flash glucose monitor and your medical records. We will also ask to complete a questionnaire at three time points.

The people in our research team at the local hospital or research centre will know that you are taking part. The doctors looking after you when you come to hospital will also know that you are in the study. Your medical notes may be looked at by staff from Swansea and Cardiff Universities or NHS and regulatory auditors who will be checking that the study is being done correctly. If you agree, we will also tell your family doctor (GP) that you are in the study.

People at the research laboratories will not know who you are when they test your samples. You will be given a study number to replace your name so any study samples and data related to you will be anonymised. The questionnaires and the data from your flash glucose monitor will also use a study number instead of your name.

All information collected about you during the study will be kept by the research nurse in a locked cabinet and entered onto a secure database. Only people with the password can open up the database.

Cardiff University is the sponsor for this study based in the United Kingdom. Cardiff University will be using information from you and your medical records in order to undertake this study and will act as the data controller for this study. This means that we are responsible for looking after your information and using it properly. Cardiff University will keep identifiable information about you for 25 years after the study has finished.

Your rights to access, change or move your information are limited, as we need to manage your information in specific ways in order for the research to be reliable and accurate. If you withdraw from the study, we will keep the information about you that we have already obtained. To safeguard your rights, we will use the minimum personally-identifiable information possible.

You can find out more about how we use your information by contacting inforequest@cardiff.ac.uk.

WHAT WILL HAPPEN TO ANY SAMPLES I GIVE?

We want to test your blood and urine to better understand your diabetes and how the study medicine or placebo affects your diabetes. The blood samples will be used to test:

1. The amount of insulin your body still makes before and after taking part in the study.
2. Your average blood glucose levels
3. The antibodies to the insulin making cells
4. General health checks – anaemia, kidneys, liver etc
5. How much of the study medication is in your blood.
6. How your body's immune system is reacting to the study medication.

Scientists in laboratories around the UK will look at your anonymised blood samples. These samples will be stored in a safe place. Some of your blood samples will be sent to a laboratory in Europe and either America or Canada for special testing to find out how much of the study medicine is present.

We would like to keep any leftover blood samples in a special tissue repository permanently - we will ask for your permission to do this. The samples will only be accessed by scientists who have special permission to do so. The samples might be sent outside the UK to other research teams in Europe or countries such as America and Canada if you agree to this. These other teams must have permission from us to use your sample before we send it. If you

For peer review only - http://bmjopen.bmj.com/site/about/guidelines.xhtml

do not want to agree to this, your samples will be destroyed after they have been analysed.

WILL ANY GENETIC TESTS BE DONE?

We will use your blood samples to help us study the genes involved with diabetes and the immune system. These samples will not have your name on them and will not be used for any other reason without your permission.

WHAT WILL HAPPEN TO THE RESULTS OF THIS STUDY?

The full results of this study will not be known until the last patient has completed their tests, which may take more than 5 years. The research results will be reported in scientific publications and meetings but you will not be identified by name at all. If you are interested in receiving a summary of the research results, we can arrange this.

WHO IS ORGANISING AND FUNDING THE STUDY?

The study is being organised by researchers at Cardiff University and Swansea University.

It is being funded by a grant from the National Institute for Health and Research (NIHR).

WHERE IS THE STUDY BEING DONE?

The study is being done at hospitals and research centres across England, Wales and Scotland.

WHO HAS CHECKED THIS STUDY?

Before any research goes ahead it has to be checked by a Research Ethics Committee. This is a group of people who make sure that the research is OK to do and to make sure that the patient will be safe. This study has been looked at by Wales REC 3. As this study is looking at a medicine, it has also been approved by the government's Medicine and Healthcare products Regulatory Authority (MHRA) who check that the researchers carry out the study safely. It has also been checked by national and local NHS organisations to make sure that the study can be done using their site and staff.

WHAT SHOULD I DO NOW?

If you are interested in taking part, or have any questions please contact one of the following people:

**Name:**	**Name:**	**Name:** (only if needed)
**Role:** Principal Investigator	**Role:** Research nurse	**Role:**
**Tel. No:**	**Tel. No:**	**Tel. No:**
**Email:**	**Email:**	**Email:**

Alternatively, you may want to speak to someone at the USTEKID Trial Office who are managing the study, based at Swansea University. The Trial Manager's details are below:

Name: Dr Kym Thorne
Tel. No: 01792 606372 (direct) or 01792 606545 for Swansea Trials Unit
Email: ustekid@swansea.ac.uk
Address: Floor 2, Institute of Life Sciences 2, Swansea University Medical School, Singleton Park, Swansea SA2 8PP

Thank you for taking the time to read this information sheet and for considering taking part in this research study

[Insert local headers]

National Institute for Health Research

CARDIFF UNIVERSITY PRIFYSGOL CAERDYDD

Swansea University Prifysgol Abertawe Swansea Trials Unit Uned Dreialon Abertawe

A research study to see if the medicine Ustekinumab can make diabetes easier to manage

CONTACT DETAILS FOR STUDY TEAM:

NURSE:

DOCTOR:

EMERGENCIES:

FOR PARENTS OF YOUNG PEOPLE AGED 16-18 YEARS OLD

We would like to invite your child to help us with our research study. It is important for you to understand why the research is being done and what it will involve as we also need some information from you. Please take time to read the following information carefully and discuss it with friends and relatives if you wish. If anything is unclear or you need to know more, please ask us.

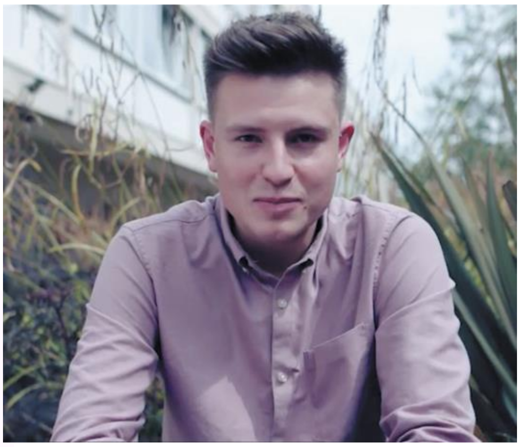

Please look at our video explaining the trial at www.type1diabetesresearch.org.uk/current-trials. The blue box below and the video contains the key points about the study. If you would like to know more, please read the rest of this leaflet.

KEY POINTS ABOUT THE STUDY:

- We want to see if the study medicine, **Ustekinumab**, can make Type 1 diabetes (the type your child has) easier to manage. The medicine works by "protecting" some of the cells in the pancreas that still produce insulin from attack by the immune system.
- The study will involve your child having an injection every 1-3 months with either the study medicine, Ustekinumab or a placebo (a "dummy medicine"). These injections are given under the skin just like insulin injections and will be done by the study doctor or nurse. Neither you nor the research team will know if your child is receiving the study medicine or the placebo.
- The medicine is already being used to treat other illnesses quite safely.
- Your child will come to your local hospital or research centre for 10 study visits over a 15 month period, but where possible this will be on the same day as their routine hospital visits. The first two will check if you are eligible to take part. Three of the visits can be sometimes done by a research nurse at home. You will be asked to come with them for three of these study visits to complete a questionnaire.
- We will ask your child to provide extra blood and urine samples for the study.
- We will provide your child with a flash blood glucose monitor (Freestyle Libre) to wear for 2 weeks before each visit. They can keep the monitor for use at home for the whole time of the study.
- Your child will be offered a small gift voucher for each visit.
- You can stop taking part in the study at any time and you do not have to give a reason why. It will not affect your child taking part in the study.

For peer review only - http://bmjopen.bmj.com/site/about/guidelines.xhtml

WHY IS THIS STUDY BEING DONE?

This study is being done to see if a medicine called **Ustekinumab** can help to "protect" the cells in the body that produce insulin in young people recently diagnosed with Type 1 Diabetes . It is caused by the body's own immune system damaging the cells in the pancreas that make insulin. Our aim is to develop a treatment that can slow this process by targeting the immune cells causing the damage.

At the time of diagnosis, most children have 10-20% of their insulin-producing cells still working. It usually takes between 1 and 5 years before they stop working completely. Sometimes these last few working cells can make enough insulin to make blood sugar levels stable and easier to control—this is called the "Honeymoon period". This period is only temporary and doesn't last. Ustekinumab, the study medicine, may make this period last longer by reducing the damaging effects of the immune system on the remaining insulin-producing cells in the pancreas.

Ustekinumab is currently given to adults and teenagers with particular skin and bowel problems and it is known to be safe and effective at treating those conditions.

WHY HAS MY CHILD BEEN ASKED TO TAKE PART?

Your child has been chosen because they are aged 12 - 18 years old and has recently been diagnosed with Type 1 diabetes. You are being told about the study because if they agree to take part, we need a parent/carer to complete three questionnaires for us.

DO I HAVE TO TAKE PART?

No. It is completely up to you whether or not you take part. It will not affect your child taking part in the study. Both you and your child are free to change your minds at any time.

If your child is interested in this study then they can let us know by calling one of the people listed at the end of their information sheet and a member of the research team will contact them and you to explain more about the study and answer any questions you may both have.

WHAT HAPPENS ONCE MY CHILD AGREEDS TO TAKE PART?

Before we can start the treatment, we need to check that your child is eligible to take part in the study - this is called "screening". Your child will be asked to come to your local hospital or research centre to talk about the study. This is where you will both be able to ask questions.

You will be invited to attend the screening visits and asked to consider whether you would be happy to consent to completing three questionnaires, one at the second screening visit, one 28 weeks after their first study treatment and one 52 weeks after their first study treatment. The person consenting should be the person who will complete all three study questionnaires. If you are happy to do so, we will ask you to sign a consent form and begin completing the first questionnaire at the second screening visit.

If you would like more details on your child's role in the study, please ask to see their information sheet which details the visits and tests being done.

WHAT WILL I BE ASKED TO DO?

As well as attending with your child to your local hospital or research centre for study visits that require you to complete questionnaires, there are a few things we will ask your child to do during the study which may involve your help:

- Your child will be asked to complete a diary between study visits to record:
  - a) How much insulin they take during the study (for the two weeks before every study visit)
  - b) If they feel unwell or have to take any medicines during the study.
  - c) If they have any hypoglycaemic (low blood glucose level) episodes that need treating.
- We will ask them to test their blood glucose levels at home for at least 2 weeks before each visit using our FREE Abbott Freestyle Libre glucose monitoring system so they don't need to do extra finger prick tests for the trial. They will need to wear a sensor on their arm. We will show you both how it works. They are free to use the monitor at home for the rest of the time of study if they want to. We will give them enough sensors to allow this for a year.

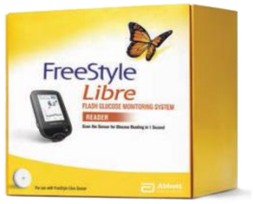

For peer review only - http://bmjopen.bmj.com/site/about/guidelines.xhtml

- We will ask them to give a blood spot sample which they can do at home. They will need to do this once a week for 28 weekly then every month for the next 6 months. We will show you both how to do this and will provide envelopes and pay for the postage.
- You will be asked to make sure that your child does not have certain vaccinations before, during and immediately after the study. If they need a vaccination, for example if they are travelling abroad, you must tell the study doctor or nurse immediately.

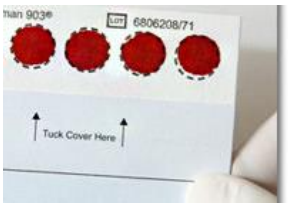

WILL THE STUDY HELP MY CHILD?

Your child has been allocated to the group that receives the study medicine Ustekinumab, it is possible that it will help their pancreas make insulin for longer. However we cannot say this for certain until we have completed this study. During the study your child's diabetes will be very closely monitored. This will include regular check-ups with your local diabetes team including routine blood testing. You and your child will have more time with the research team to discuss their diabetes and ask questions than at a normal clinic appointment.

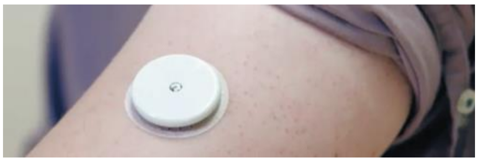

Your child will be provided with a FREE Abbott Freestyle Libre blood glucose monitoring system. They can use this to check their blood glucose levels while they are in the study.

Injections with the study medicine or the placebo will be done at weeks 0, 4, 12, 20, 28, 36 and 44. After this, no more treatments will be given to your child.

WHAT HAPPENS WHEN THE STUDY STOPS?

We will collect all the information together and we will decide if the study medicine can help people with Type 1 Diabetes make their own insulin for longer. If it does then we will carry out a bigger version of this study.

WHAT IF NEW INFORMATION COMES ALONG?

Sometimes during research, we get new information about the treatment being studied. If this happens, we will tell you about it and discuss whether you want to continue in the study.

WHAT IF I DO NOT WANT TO TAKE PART ANYMORE?

Just let the study doctor or research nurse know about your decision and we will make sure that you are not asked to complete further questionnaires. If you withdraw, this will not affect your child taking part in the study.

WHAT IF THERE IS A PROBLEM OR SOMETHING GOES WRONG?

If there are any problems, please contact the study team using the details at the end of this form.

If you feel overwhelmed by your child's recent diagnosis, you can call the local [title] on [tel number / email] and they can talk through your concerns with you.

If you are unhappy about the conduct of the study and wish to complain, you can do this through: (name and contact details of appropriate organisations – site specific).

WHAT ARE THE POSSIBLE SIDE EFFECTS FOR ME IN PARTICIPATING IN THE STUDY?

You are only being asked to complete three short questionnaires. We do not anticipate any risk being involved.

The risks for your child for taking part in the study are fully explained in their information sheet.

WILL I RECEIVE ANY PAYMENT FOR TAKING PART?

You will be able to claim back your travel expenses getting your child to the local hospital or research centre.

WHAT INFORMATION WILL YOU COLLECT AND HOW WILL IT BE KEPT PRIVATE?

We will ask for your name and contact details as well as your child's name so that the research nurse can keep in touch and manage your visits. We will also collect the information you record on your questionnaire and enter it into a database.

The people in our research team at the local hospital or research centre will know that your child is taking part. The doctors looking after your child when you come to hospital will also know that they are in the study. Their medical

For peer review only - http://bmjopen.bmj.com/site/about/guidelines.xhtml

notes may be looked at by staff from Swansea and Cardiff Universities who will be checking that the study is being done correctly. If your child has agreed, we will also tell your family doctor (GP) that they are in the study.

You and your child will be given a study number to replace their name so any study samples and information related to you both will be anonymised.

All information collected about you and your child during the study will be kept by the research nurse in a locked cabinet and entered onto a secure database. Only people with the password can open up the database.

Cardiff University is the sponsor for this study based in the United Kingdom. Cardiff University will be using information from you and your medical records in order to undertake this study and will act as the data controller for this study. This means that we are responsible for looking after your information and using it properly. Cardiff University will keep identifiable information about you for 25 years after the study has finished.

Your rights to access, change or move your information are limited, as we need to manage your information in specific ways in order for the research to be reliable and accurate. If you withdraw from the study, we will keep the information about you that we have already obtained. To safeguard your rights, we will use the minimum personally-identifiable information possible. You can find out more about how we use your information by contacting inforequest@cardiff.ac.uk.

WHAT WILL HAPPEN TO THE RESULTS OF THIS STUDY?

The full results of this study will not be known until the last patient has completed their tests, which may take more than 5 years. The research results will be reported in scientific publications and meetings but you will not be identified by name at all. If you are interested in receiving a summary of the research results, we can arrange this.

WHO IS ORGANISING AND FUNDING THE STUDY?

The study is being organised by researchers at Cardiff University and Swansea University. It is being funded by a grant from the National Institute for Health and Research (NIHR).

WHERE IS THE STUDY BEING DONE?

The study is being done at hospitals and research centres across England, Wales and Scotland.

WHO HAS CHECKED THIS STUDY?

Before any research goes ahead it has to be checked by a Research Ethics Committee. This is a group of people who make sure that the research is OK to do and to make sure that the patient will be safe. This study has been looked at by Wales REC 3. As this study is looking at a medicine, it has also been approved by the government's Medicine and Healthcare products Regulatory Authority (MHRA) who check that the researchers carry out the study safely. It has also been checked by national and local NHS organisations to make sure that the study can be done using their site and staff.

WHAT SHOULD I DO NOW?

If you are interested in taking part, or have any questions please contact one of the following people:

**Name:**	**Name:**	**Name:** (only if needed)
**Role:** Principal Investigator	**Role:** Research nurse	**Role:**
**Tel. No:**	**Tel. No:**	**Tel. No:**
**Email:**	**Email:**	**Email:**

Alternatively, you may want to speak to someone at the USTEKID Trial Office who are managing the study, based at Swansea University. The Trial Manager's details are below:

Name: Dr Kym Thorne
Tel. No: 01792 606372 (direct) or 01792 606545 for Swansea Trials Unit
Email: ustekid@swansea.ac.uk
Address: Floor 2, Institute of Life Sciences 2, Swansea University Medical School, Singleton Park, Swansea SA2 8PP

Thank you for taking the time to read this information sheet and for considering taking part in this research study

For peer review only - http://bmjopen.bmj.com/site/about/guidelines.xhtml

USTEKID

Phase II multi-centre, double-blind, randomised trial of Ustekinumab in adolescents with new-onset type 1 diabetes (USTEKID)

**Chief Investigator:**	Prof Colin Dayan	**Principal Investigator:**	
**Site ID:**		**Participant study number:**	
**Participant name (in capitals):**			

ASSENT FORM FOR YOUNG ADOLESCENTS (AGED 12-15y)

(to be completed by the child with help from and their parent/carer)

Circle either Yes or No to the following

Have you read the information sheet about the study (or has someone read it to you)?	YES	NO
Has somebody explained this study to you?	YES	NO
Do you understand what this study is about?	YES	NO
Have you asked all the questions you want?	YES	NO
Has someone answered your questions in a way that you understand?	YES	NO
Do you understand that it is OK to stop taking part at any time?	YES	NO
Are you happy to give samples of blood and wee (urine)?	YES	NO
Are you happy to take part?	YES	NO

If you do not want to take part, do not sign your name.
If you are not happy with what you have circled YES to, do not sign your name.

If you do want to take part, you should write your name clearly below.

Your name ____

Please write the date ____________________

The person who explained the study to you also needs to sign this form:

______________________ ______________________ ______________________
Researcher Name Signature Date

For peer review only

For peer review only - http://bmjopen.bmj.com/site/about/guidelines.xhtml

Insert logos

Phase II multi-centre, double-blind, randomised trial of Ustekinumab in adolescents with new-onset type 1 diabetes (USTEKID)

**Chief Investigator:**	Prof Colin Dayan	**Principal Investigator:**	
**Site ID:**		**Participant study number:**	

CONSENT FORM FOR PARENTS/CARERS OF YOUNG ADOLESCENTS (AGED 12-15y)

Please initial boxes

1. I confirm that I have read and understand the 12-15y Parent Information Sheet dated (………………) (version......) for the above study. I have had the opportunity to consider the information, ask questions and have had these answered satisfactorily.
2. I understand that my child's participation is voluntary and that we are free to withdraw at any time without giving any reason, without his/her medical care or legal rights being affected.
3. I agree to my child attending screening and study visits and to being randomised to receive either the study medicine or the placebo.
4. I agree that my child can provide urine and blood samples for the study.
5. I agree that my child can do dried blood spot testing at home for the study.
6. I agree that my child can wear the FreeStyle Libre glucose monitor at least two weeks prior to each study visit.
7. I agree that my child can complete diaries and questionnaires for the study.
8. I agree to complete study questionnaires myself.
9. I understand that the information collected from me and my child in study questionnaires will be viewed by the research team and will be stored securely.
10. I agree that if my child is involved in actions which may lead to pregnancy, they will take adequate contraception (hormonal based contraception, barrier contraception, abstinence) until 4 months following the date of final treatment.
11. I give permission for relevant sections of my child's medical notes and data collected during the study to be looked at by responsible individuals from the USTEKID research team, from regulatory authorities or from Cardiff University (as Sponsor), where it is relevant to my child taking part in this research.
12. I understand that the information collected about my child may be used to support other research in the future, and may be shared anonymously with other researchers.
13. I understand that the information held and maintained in local hospital records and other central UK NHS bodies may be used to help contact myself or my child, or to provide information about my child's health status during the study follow up.
14. I understand and agree that my child's anonymised blood samples may be used for analysis by the study or other relevant studies if they obtain the relevant permissions.
15. I agree to my child's anonymised blood samples being stored in a Human Tissue Authority (HTA) repository for future ethically approved studies.
16. I agree that my child's anonymised blood samples may be transported within and outside the European Union for analysis in specialist laboratories.
17. I agree to my child's GP being notified of his/her involvement in the study, including any necessary exchange of information about my child between my GP and the research team.
18. I agree to my child taking part in the above study.

For the participant's parent/carer

NAME__________________________ SIGNATURE_______________________ DATE_______________

For the person taking consent

NAME__________________________ SIGNATURE_______________________ DATE_______________

For peer review only - http://bmjopen.bmj.com/site/about/guidelines.xhtml

Insert logos

Phase II multi-centre, double-blind, randomised trial of Ustekinumab in adolescents with new-onset type 1 diabetes (USTEKID)

Chief Investigator: Prof Colin Dayan **Principal Investigator:** ____

Site ID: ____ **Participant study number:** ____

CONSENT FORM FOR ADOLESCENTS (AGED 16-18y)

Please initial boxes

1. I confirm that I have read and understand the 16-18y Patient Information Sheet dated (………………) (version......) for the above study. I have had the opportunity to consider the information, ask questions and have had these answered satisfactorily. ☐
2. I understand that my participation is voluntary and that I am free to withdraw at any time without giving any reason, without my medical care or legal rights being affected. ☐
3. I agree to attend screening and study visits and to being randomised to receive either the study medicine or the placebo. ☐
4. I agree to provide urine and blood samples for the study. ☐
5. I agree to do dried blood spot testing at home for the study. ☐
6. I agree to wear the FreeStyle Libre glucose monitor at least two weeks prior to each study visit. ☐
7. I agree to complete diaries and questionnaires for the study. ☐
8. I agree that if I am involved in actions that may lead to pregnancy, I will take adequate contraception (hormonal based contraception, barrier contraception, abstinence) until 4 months following the date of final treatment. ☐
9. I give permission for relevant sections of my medical notes and data collected during the study to be looked at by responsible individuals from the USTEKID research team, from regulatory authorities or from Cardiff University (as Sponsor), where it is relevant to my taking part in this research. ☐
10. I understand that the information collected about me may be used to support other research in the future, and may be shared anonymously with other researchers. ☐
11. I understand that the information held and maintained in local hospital records and other central UK NHS bodies may be used to help contact me or provide information about my health status during the study follow up. ☐
12. I understand and agree that my anonymised blood samples may be used for analysis by the study or other relevant studies if they obtain the relevant permissions. ☐
13. I agree to my anonymised blood samples being stored in a Human Tissue Authority (HTA) repository for future ethically approved studies. ☐
14. I agree that my anonymised blood samples may be transported within and outside the European Union for analysis in specialist laboratories. ☐
15. I agree to my GP being notified of my involvement in the study, including any necessary exchange of information about me between my GP and the research team. ☐
16. I agree to take part in the above study. ☐

For the participant

NAME__________________ SIGNATURE__________________ DATE__________________

For the person taking consent

NAME__________________ SIGNATURE__________________ DATE__________________

For peer review only

USTEKID | Noah's Ark Children's Hospital for Wales / Ysbyty Plant Cymru | GYARU | GIG CYMRU NHS WALES Bwrdd Iechyd Prifysgol Caerdydd a'r Fro / Cardiff and Vale University Health Board

Phase II multi-centre, double-blind, randomised trial of Ustekinumab in adolescents with new-onset type 1 diabetes (USTEKID)

**Chief Investigator:**	Prof Colin Dayan	**Principal Investigator:**	Prof John Gregory
**Site ID:**		**Participant study number:**	
**Participant name (in capitals):**			

CONSENT FORM FOR PARENT/CARER OF ADOLESCENTS (AGED 16-18y)

Please initial the boxes

1. I confirm that I have read and understand the 16-18y Parent Information Sheet dated 2nd July 2019, version 3 for the above study. I have had the opportunity to consider the information, ask questions and have had these answered satisfactorily. ☐
2. I understand that my participation is voluntary and that I am free to withdraw at any time without giving any reason, without my child's medical care or legal rights being affected. ☐
3. I agree to complete study questionnaires. ☐
4. I understand that the information collected from me in study questionnaires will be viewed by the research team and will be stored securely. ☐
5. I agree to take part in the above study. ☐

For the participant's parent/carer

NAME______________________________SIGNATURE___________________________ DATE_________________

For the person taking consent

NAME______________________________SIGNATURE___________________________ DATE_________________

For peer review only

For peer review only - http://bmjopen.bmj.com/site/about/guidelines.xhtml

Reporting checklist for protocol of a clinical trial.

Based on the SPIRIT guidelines.

Instructions to authors

Complete this checklist by entering the page numbers from your manuscript where readers will find each of the items listed below.

Your article may not currently address all the items on the checklist. Please modify your text to include the missing information. If you are certain that an item does not apply, please write "n/a" and provide a short explanation.

Upload your completed checklist as an extra file when you submit to a journal.

In your methods section, say that you used the SPIRIT reporting guidelines, and cite them as:

Chan A-W, Tetzlaff JM, Gøtzsche PC, Altman DG, Mann H, Berlin J, Dickersin K, Hróbjartsson A, Schulz KF, Parulekar WR, Krleža-Jerić K, Laupacis A, Moher D. SPIRIT 2013 Explanation and Elaboration: Guidance for protocols of clinical trials. BMJ. 2013;346:e7586

For peer review only

		Reporting Item	Page Number
**Administrative information**			
Title	#1	Descriptive title identifying the study design, population, interventions, and, if applicable, trial acronym	1

Trial registration	#2a	Trial identifier and registry name. If not yet registered, name of intended registry	2
Trial registration: data set	#2b	All items from the World Health Organization Trial Registration Data Set	Not included – repository agreement not currently in place
Protocol version	#3	Date and version identifier	2
Funding	#4	Sources and types of financial, material, and other support	14
Roles and responsibilities: contributorship	#5a	Names, affiliations, and roles of protocol contributors	1 & 15
Roles and responsibilities: sponsor contact information	#5b	Name and contact information for the trial sponsor	14
Roles and responsibilities: sponsor and funder	#5c	Role of study sponsor and funders, if any, in study design; collection, management, analysis, and interpretation of data; writing of the report; and the decision to submit the report for publication, including whether they will have ultimate authority over any of these activities	14

For peer review only

Roles and responsibilities: committees	#5d	Composition, roles, and responsibilities of the coordinating centre, steering committee, endpoint adjudication committee, data management team, and other individuals or groups overseeing the trial, if applicable (see Item 21a for data monitoring committee)	14
**Introduction**			
Background and rationale	#6a	Description of research question and justification for undertaking the trial, including summary of relevant studies (published and unpublished) examining benefits and harms for each intervention	3
Background and rationale: choice of comparators	#6b	Explanation for choice of comparators	3
Objectives	#7	Specific objectives or hypotheses	3 and Table 1
Trial design	#8	Description of trial design including type of trial (eg, parallel group, crossover, factorial, single group), allocation ratio, and framework (eg, superiority, equivalence, non-inferiority, exploratory)	3
**Methods: Participants, interventions, and outcomes**			

For peer review only

Study setting	#9	Description of study settings (eg, community clinic, academic hospital) and list of countries where data will be collected. Reference to where list of study sites can be obtained	3
Eligibility criteria	#10	Inclusion and exclusion criteria for participants. If applicable, eligibility criteria for study centres and individuals who will perform the interventions (eg, surgeons, psychotherapists)	5 and Table 2
Interventions: description	#11a	Interventions for each group with sufficient detail to allow replication, including how and when they will be administered	10
Interventions: modifications	#11b	Criteria for discontinuing or modifying allocated interventions for a given trial participant (eg, drug dose change in response to harms, participant request, or improving / worsening disease)	11
Interventions: adherance	#11c	Strategies to improve adherence to intervention protocols, and any procedures for monitoring adherence (eg, drug tablet return; laboratory tests)	Not applicable. Intervention given at site
Interventions: concomitant care	#11d	Relevant concomitant care and interventions that are permitted or prohibited during the trial	Table 2
Outcomes	#12	Primary, secondary, and other outcomes, including the specific measurement variable (eg, systolic	Table 1

For peer review only

		blood pressure), analysis metric (eg, change from baseline, final value, time to event), method of aggregation (eg, median, proportion), and time point for each outcome. Explanation of the clinical relevance of chosen efficacy and harm outcomes is strongly recommended	
Participant timeline	#13	Time schedule of enrolment, interventions (including any run-ins and washouts), assessments, and visits for participants. A schematic diagram is highly recommended (see Figure)	Table 3, Figure 1
Sample size	#14	Estimated number of participants needed to achieve study objectives and how it was determined, including clinical and statistical assumptions supporting any sample size calculations	12
Recruitment	#15	Strategies for achieving adequate participant enrolment to reach target sample size	5
**Methods: Assignment of interventions (for controlled trials)**			
Allocation: sequence generation	#16a	Method of generating the allocation sequence (eg, computer-generated random numbers), and list of any factors for stratification. To reduce predictability of a random sequence, details of any planned	7

For peer review only

		restriction (eg, blocking) should be provided in a separate document that is unavailable to those who enrol participants or assign interventions	
Allocation concealment mechanism	#16b	Mechanism of implementing the allocation sequence (eg, central telephone; sequentially numbered, opaque, sealed envelopes), describing any steps to conceal the sequence until interventions are assigned	7
Allocation: implementation	#16c	Who will generate the allocation sequence, who will enrol participants, and who will assign participants to interventions	7
Blinding (masking)	#17a	Who will be blinded after assignment to interventions (eg, trial participants, care providers, outcome assessors, data analysts), and how	7
Blinding (masking): emergency unblinding	#17b	If blinded, circumstances under which unblinding is permissible, and procedure for revealing a participant's allocated intervention during the trial	7
**Methods: Data collection, management, and analysis**			
Data collection plan	#18a	Plans for assessment and collection of outcome, baseline, and other trial data, including any related processes to promote data quality (eg, duplicate	8-10

For peer review only

		measurements, training of assessors) and a description of study instruments (eg, questionnaires, laboratory tests) along with their reliability and validity, if known. Reference to where data collection forms can be found, if not in the protocol	
Data collection plan: retention	#18b	Plans to promote participant retention and complete follow-up, including list of any outcome data to be collected for participants who discontinue or deviate from intervention protocols	10
Data management	#19	Plans for data entry, coding, security, and storage, including any related processes to promote data quality (eg, double data entry; range checks for data values). Reference to where details of data management procedures can be found, if not in the protocol	13-4
Statistics: outcomes	#20a	Statistical methods for analysing primary and secondary outcomes. Reference to where other details of the statistical analysis plan can be found, if not in the protocol	12 and Table 1
Statistics: additional analyses	#20b	Methods for any additional analyses (eg, subgroup and adjusted analyses)	12
Statistics: analysis population and missing data	#20c	Definition of analysis population relating to protocol non-adherence (eg, as randomised analysis), and	12

For peer review only

		any statistical methods to handle missing data (eg, multiple imputation)	
**Methods: Monitoring**			
Data monitoring: formal committee	#21a	Composition of data monitoring committee (DMC); summary of its role and reporting structure; statement of whether it is independent from the sponsor and competing interests; and reference to where further details about its charter can be found, if not in the protocol. Alternatively, an explanation of why a DMC is not needed	14
Data monitoring: interim analysis	#21b	Description of any interim analyses and stopping guidelines, including who will have access to these interim results and make the final decision to terminate the trial	12
Harms	#22	Plans for collecting, assessing, reporting, and managing solicited and spontaneously reported adverse events and other unintended effects of trial interventions or trial conduct	11
Auditing	#23	Frequency and procedures for auditing trial conduct, if any, and whether the process will be independent from investigators and the sponsor	13
**Ethics and dissemination**			

For peer review only

Research ethics approval	#24	Plans for seeking research ethics committee / institutional review board (REC / IRB) approval	14
Protocol amendments	#25	Plans for communicating important protocol modifications (eg, changes to eligibility criteria, outcomes, analyses) to relevant parties (eg, investigators, REC / IRBs, trial participants, trial registries, journals, regulators)	14
Consent or assent	#26a	Who will obtain informed consent or assent from potential trial participants or authorised surrogates, and how (see Item 32)	5
Consent or assent: ancillary studies	#26b	Additional consent provisions for collection and use of participant data and biological specimens in ancillary studies, if applicable	Not applicable
Confidentiality	#27	How personal information about potential and enrolled participants will be collected, shared, and maintained in order to protect confidentiality before, during, and after the trial	11
Declaration of interests	#28	Financial and other competing interests for principal investigators for the overall trial and each study site	14
Data access	#29	Statement of who will have access to the final trial dataset, and disclosure of contractual agreements that limit such access for investigators	13

For peer review only

Ancillary and post trial care	#30	Provisions, if any, for ancillary and post-trial care, and for compensation to those who suffer harm from trial participation	11
Dissemination policy: trial results	#31a	Plans for investigators and sponsor to communicate trial results to participants, healthcare professionals, the public, and other relevant groups (eg, via publication, reporting in results databases, or other data sharing arrangements), including any publication restrictions	13
Dissemination policy: authorship	#31b	Authorship eligibility guidelines and any intended use of professional writers	13
Dissemination policy: reproducible research	#31c	Plans, if any, for granting public access to the full protocol, participant-level dataset, and statistical code	Not yet agreed.
**Appendices**			
Informed consent materials	#32	Model consent form and other related documentation given to participants and authorised surrogates	All 8 can be provided if requested
Biological specimens	#33	Plans for collection, laboratory evaluation, and storage of biological specimens for genetic or molecular analysis in the current trial and for future use in ancillary studies, if applicable	8-10

For peer review only

None The SPIRIT checklist is distributed under the terms of the Creative Commons Attribution License CC-BY-ND 3.0. This checklist can be completed online using https://www.goodreports.org/, a tool made by the EQUATOR Network in collaboration with Penelope.ai

For peer review only
